# Casz1 and Znf101/Zfp961 differentially regulate apolipoproteins A1 and B, alter plasma lipoproteins, and reduce atherosclerosis

**DOI:** 10.1172/jci.insight.182260

**Published:** 2025-01-09

**Authors:** Abulaish Ansari, Pradeep Kumar Yadav, Liye Zhou, Binu Prakash, Laraib Ijaz, Amanda Christiano, Sameer Ahmad, Antoine Rimbert, M. Mahmood Hussain

**Affiliations:** 1 Department of Foundations of Medicine, NYU Grossman Long Island School of Medicine, Mineola, New York, USA.; 2 Department of Cell Biology, SUNY Downstate Medical Center, Brooklyn, New York, USA.; 3 Nantes Université, CNRS, INSERM, l’institut du thorax, F-44000 Nantes, France.; 4 VA New York Harbor Healthcare System, Brooklyn, New York, USA.

**Keywords:** Vascular biology, Lipoproteins

## Abstract

High apolipoprotein B–containing (apoB-containing) low-density lipoproteins (LDLs) and low apoA1–containing high-density lipoproteins (HDLs) are associated with atherosclerotic cardiovascular diseases. In search of a molecular regulator that could simultaneously and reciprocally control both LDL and HDL levels, we screened a microRNA (miR) library using human hepatoma Huh-7 cells. We identified miR-541-3p that both significantly decreases apoB and increases apoA1 expression by inducing mRNA degradation of 2 different transcription factors, Znf101 and Casz1. We found that Znf101 enhances apoB expression, while Casz1 represses apoA1 expression. The hepatic knockdown of Casz1 in mice increased plasma apoA1, HDL, and cholesterol efflux capacity. The hepatic knockdown of Zfp961, an ortholog of Znf101, reduced lipogenesis and production of triglyceride-rich lipoproteins and atherosclerosis, without causing hepatic lipid accumulation. This study identifies hepatic Znf101/Zfp961 and Casz1 as potential therapeutic targets to alter plasma lipoproteins and reduce atherosclerosis without causing liver steatosis.

## Abstract 2

We identified two transcription factors that differentially regulate LDL and HDL. Their hepatic knockdown in mice alters plasma lipoprotein profile and reduces atherosclerosis.

## Graphical abstract

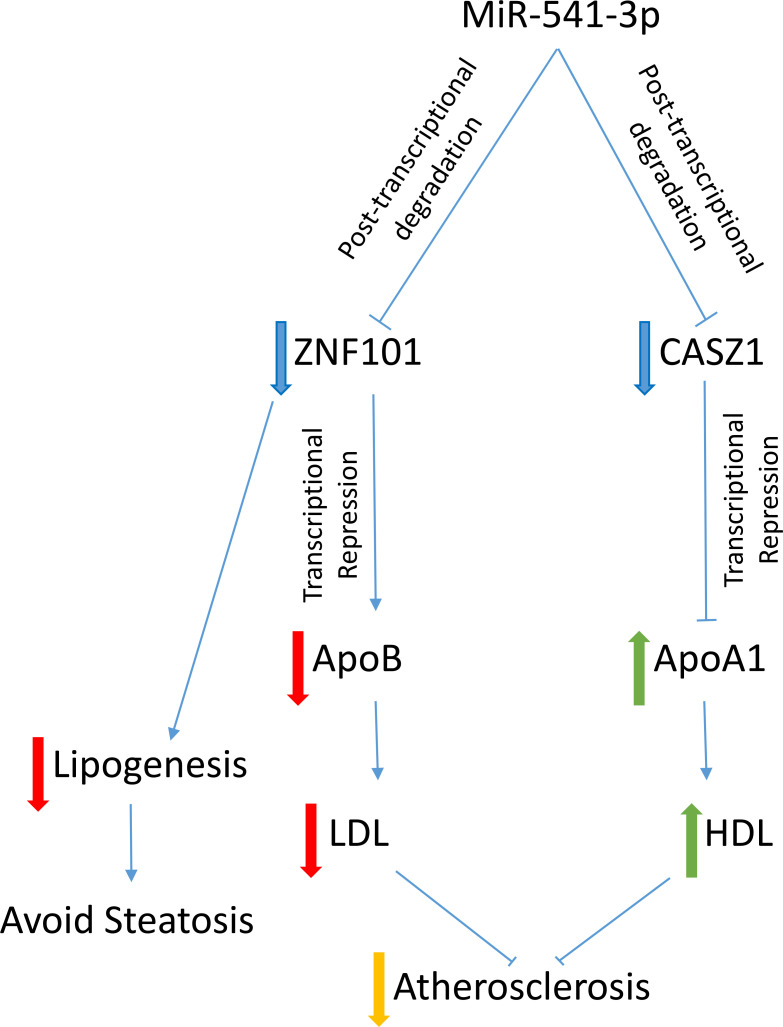

## Introduction

High plasma cholesterol levels are risk factors for atherosclerotic cardiovascular disease (ASCVD) (1, 2). Cholesterol is carried in the blood by apolipoprotein B–containing low-density lipoproteins (apoB-Lps) and apoA1-containing high-density lipoproteins (HDLs). ApoB-Lps are synthesized and secreted by the liver and the intestine to deliver endogenous and dietary lipids to peripheral tissues. Excess accumulation of modified apoB-Lps in the plasma, and their uptake by macrophages, contributes to ASCVD (3, 4). HDL extracts and transports free cholesterol from macrophages and other peripheral cells to the liver for excretion from the body (5, 6). Hence, low HDL is also associated with ASCVD. Therefore, understanding physiological and molecular mechanisms controlling plasma low-density lipoprotein (LDL) and HDL levels will aid in developing new therapeutics against ASCVD. To this end, LDL-lowering therapies are widely used to reduce heart disease. Yet, a significant portion of the population does not respond and/or is intolerant to these drugs (7, 8). Therefore, drugs have been developed to lower LDL by inhibiting hepatic production of apoB-Lps (9). However, these approaches are associated with hepatosteatosis (10). Furthermore, increasing plasma HDL therapeutically has not been effective in lowering ASCVD (6, 11). Hence, there is a need to develop new LDL-lowering therapies and to increase functional HDL levels. Notably, agents that concurrently decrease LDL and increase HDL have not been identified.

MicroRNAs (miRs), small (~22 nucleotides) noncoding RNAs, bind via their seed sequences to the 3′-untranslated regions (3′-UTRs) of target mRNAs to either degrade them or to decrease translation (12, 13). MiRs regulate many genes; therefore, miR mimics, or their inhibitors, antimiRs, can be used to understand regulation of different interrelated biological pathways. MiRs are products of endogenous genes that have evolved to regulate multiple pathways presumably to fine tune physiological processes and prevent metabolic disruptions. We hypothesized that miRs may exist in nature that could modulate plasma lipoproteins. To test this, we screened a miR library to identify miRs that reduce apoB and increase apoA1 and identified a primate-specific miR, miR-541-3p, that reduces apoB and increases apoA1 secretion in human hepatoma cells. Mechanistic studies led to identification of transcription factors (TFs), Znf101/Zfp961 and Casz1. These TFs act as an enhancer and a repressor of apoB and apoA1, respectively. Furthermore, we show that hepatic knockdown (KD) of these TFs alters the plasma lipoprotein profile and reduces atherosclerosis in mice.

## Results

### MiR-541-3p reciprocally regulates apoB and apoA1 production.

**Figure 1:**
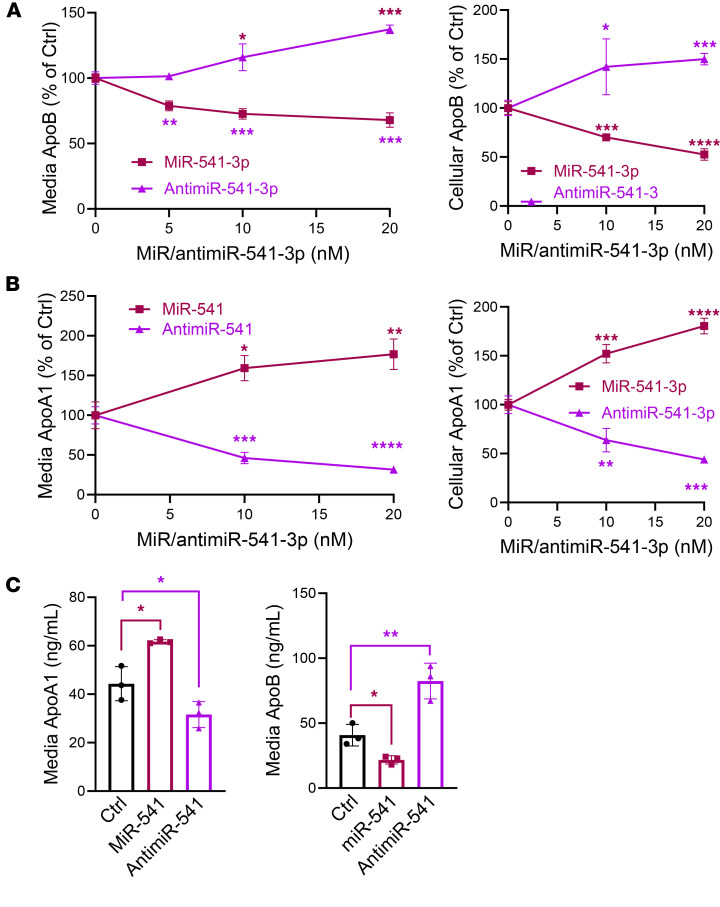
MiR-541-3p reciprocally regulates apoB and apoA1 secretion in human liver cells. (**A** and **B**) Huh-7 cells in 6-well plates were reverse transfected in triplicate with different amounts of miR-541-3p mimics or antimiR-541-3p. Control cells were transfected with a control miR (20 nM) and were used as 100% control. After 48 hours, media were changed and collected after overnight incubation. ApoB (**A**) and apoA1 (**B**) levels were quantified in media and in cell lysates in triplicate by ELISA and normalized to cellular protein levels. Data are representative of 3 experiments. (**C**) Human primary hepatocytes were seeded in 24-well plates. After 1 day, they were transfected in triplicate with miR-541-3p mimics or antimiR-541-3p (10 nM, *n* = 3). After 16 hours, media were replaced with fresh media. After 24 hours, media were used to quantify apoB and apoA1 by ELISA in triplicate. *P < 0.05; **P < 0.01; ***P < 0.001; ****P < 0.0001 by ordinary 1-way ANOVA pairwise multiple comparisons, where different groups were compared with control (**A** and **B**) or unpaired *t* test (**C**).

We transfected a human miR–mimic library into human hepatoma Huh-7 cells and quantified secreted apoB and apoA1 (Supplemental Figure 1A and Supplemental Table 1; supplemental material available online with this article; https://doi.org/10.1172/jci.insight.182260DS1). The screening performed in duplicate plates showed high correlation (Supplemental Figure 1B) and good internal reproducibility, as different members of the same miR families that share the same seed sequence similarly reduced apoB and apoA1 secretion (Supplemental Figure 1C). Additionally, miR-30c-5p and miR-33a-5p reduced apoB and apoA1, respectively, (Supplemental Table 1) consistent with earlier studies (14, 15). In this study, we selected miR-541-3p for detailed studies because it decreased apoB secretion by 58% ± 9%, and increased apoA1 secretion by 95% ± 3%. Increasing concentrations of miR-541-3p and antimiR-541-3p significantly decreased and increased, respectively, media and cellular apoB in a dose-dependent manner, as measured by ELISA and Western blotting (Figure 1A and Supplemental Figure 2, A–C). In contrast, miR-541-3p significantly increased secreted and cellular apoA1 in a dose-dependent manner, whereas antimiR-541-3p decreased apoA1 protein (Figure 1B and Supplemental Figure 2, D and F). Similar reciprocal modulation of apoB and apoA1 secretion was observed in human primary hepatocytes (Figure 1C). MiR-541-3p had no effect on *MTTP* or *ABCA1* mRNA levels, proteins critical for apoB-Lps and HDL biosynthesis (Supplemental Figure 2G). These studies suggested that miR-541-3p simultaneously and reciprocally regulates apoB and apoA1 production.

### MiR-541-3p mimics decrease apoB production by reducing Znf101 expression.

**Figure 2:**
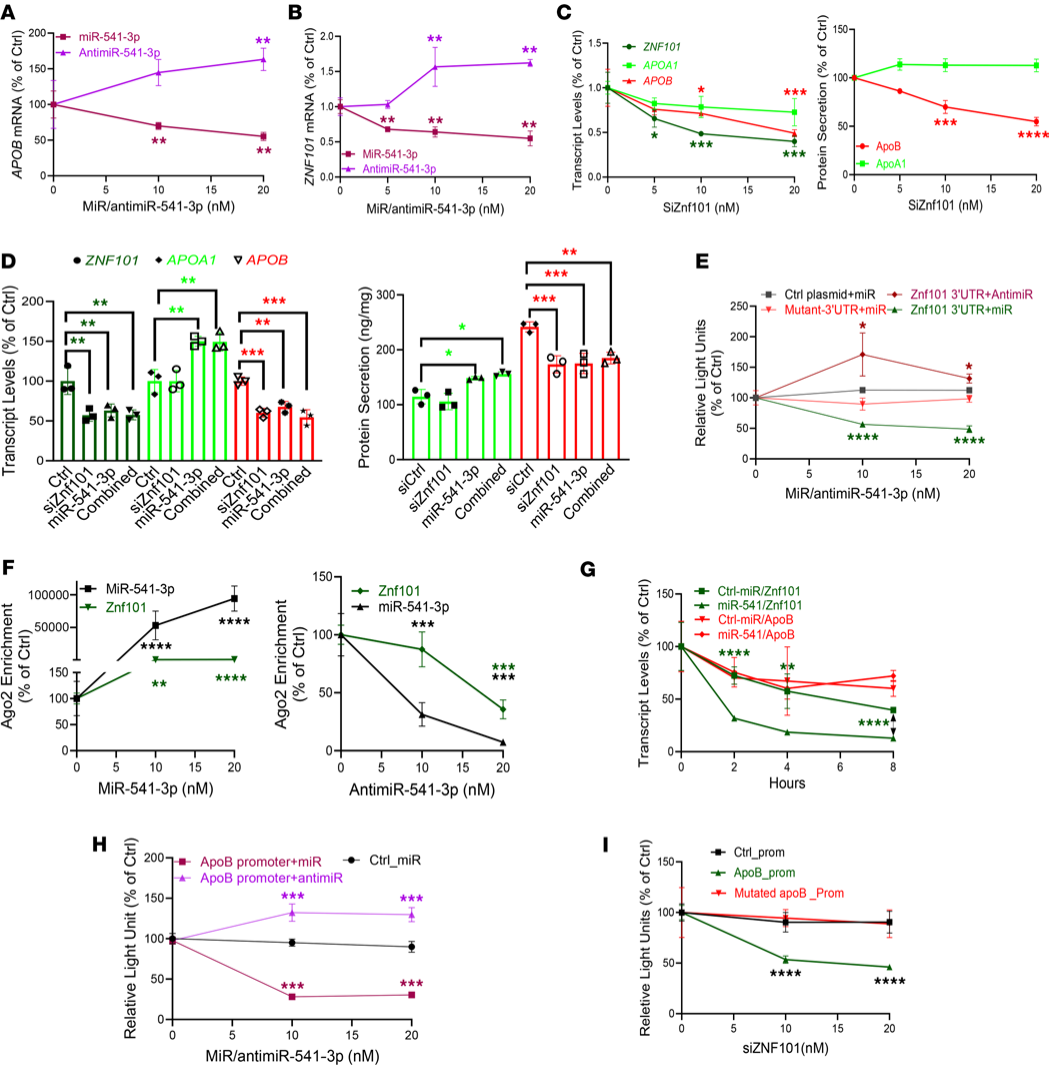
Regulation of apoB by miR-541-3p. (**A** and **B**) Huh-7 cells were transfected (*n* = 3) with different amounts of miR-541-3p or antimiR-541-3p, and different mRNA levels were quantified in triplicate. (**C**) Cells were transfected (*n* = 3) with different concentrations of siZnf101. After 48 hours, mRNA levels were quantified (left). Media were used to quantify apoB/apoA1 protein levels (right). (**D**) Huh-7 cells were transfected (*n* = 3) with siZnf101 (21 nM) or miR-541-3p mimics (10 nM), alone or in combination. Changes in mRNA were quantified in cell lysates (left), and proteins in media (right). (**E**) Cells were forward transfected (*n* = 3) with plasmids (5 μg) expressing the dual reporter *Gaussia* luciferase/secreted alkaline phosphatase under control of the Znf101 3′-UTR or a control plasmid without the Znf101 3′-UTR. The next day, cells were reverse transfected (*n* = 3) with miR-541-3p mimics or antimiR-541-3p. After 48 hours, media were assayed for luciferase and alkaline phosphatase activities. (**F**) Cells transfected (*n* = 3) with miR-541-3p mimics (left) or antimiR-541-3p (right) were used for Ago2 immunoprecipitation and measurements of miR-541-3p and Znf101 mRNA. (**G**) Cells were transfected (*n* = 3) with 20 nM miR-541-3p mimics or a control miR. After 18 hours, cells were treated with actinomycin D (10 μg/mL). At indicated times, Znf101 and apoB mRNA levels were quantified. (**H**) Cells were transfected (*n* = 3) with plasmids expressing luciferase under the control of the apoB or cytomegalovirus promoter. The next day, cells were distributed and transfected (*n* = 3) with different amounts of miR-541-3p mimics or antimiR-541-3p. After 48 hours, luciferase activity was measured in conditioned media. (**I**) Cells were transfected (*n* = 3) with plasmids expressing luciferase under control of the wild-type or mutated apoB promoter. The next day, cells were transfected with different amounts of siZnf101 (*n* = 3). After 48 hours, conditioned media were used to measure luciferase activity. *P < 0.05; **P < 0.01, ***P < 0.001; ****P < 0.0001 by ordinary 1-way ANOVA pairwise multiple comparisons, where different groups were compared with control.

To understand the mechanisms underlying how miR-541-3p regulates apoB expression, we transfected Huh-7 cells with miR-541-3p mimics and antimiR-541-3p and quantified mRNA levels. MiR-541-3p mimics decreased *APOB* mRNA, while antimiR-541-3p had the opposite effect (Figure 2A). Using several online algorithms, we found that miR-541-3p is not predicted to pair with the 3′-UTR of *APOB* mRNA or *APOB* promoter sequences. Therefore, we hypothesized that miR-541-3p might indirectly modulate mRNA by targeting TFs regulating *APOB* expression. Using online tools, we identified 7 TFs that could regulate apoB expression, are targets of miR-541-3p, and are expressed in Huh-7 cells (Supplemental Figure 3, A and B). Notably, miR-541-3p mimics significantly decreased the expression of *ZNF101* mRNA, while antimiR-541-3p increased it (Figure 2B and Supplemental Figure 3C). Reducing Znf101 with small interfering RNA (siZNF101) significantly decreased *APOB* transcript levels and protein secretion without affecting apoA1 (Figure 2C). The expression of miR-541-3p mimics, independently or combined with siZNF101, revealed that both miR-541-3p and Znf101 regulate apoB through the same pathway (Figure 2D and Supplemental Figure 3D). These studies indicated that miR-541-3p regulates Znf101 to decrease apoB expression.

To learn how miR-541-3p regulates Znf101, we hypothesized that miR-541-3p interacts with the 3′-UTR of *ZNF101* mRNA and induces its degradation. To test this, we used 4 approaches. First, we observed that the *ZNF101* mRNA 3′-UTR contains a miR-541-3p–interacting site conserved in primates (Supplemental Figure 3E). Second, we asked whether miR-541-3p interacts with the Znf101 3′-UTR. To answer this, we obtained a plasmid for the expression of luciferase with the 3′-UTR from *ZNF101* mRNA. *ZNF101* 3′-UTR activity was decreased and increased with increasing concentrations of miR-541-3p and anitmiR, respectively (Figure 2E). When we mutated the 3′-UTR of *ZNF101,* we found that miR-541-3p mimics were unable to decrease luciferase expression (Figure 2E and Supplemental Figure 3F). Third, we determined whether miR-541-3p interacts with *ZNF101* mRNA in Ago2 complexes. Expression of miR-541-3p mimics enriched miR-541-3p and *ZNF101* mRNA, while expression of antimiR-541-3p depleted their levels in Ago2 complexes (Figure 2F). Fourth, we treated cells with actinomycin D to inhibit gene transcription and studied posttranscriptional mRNA degradation in miR-541-3p mimic–treated cells. Posttranscriptional degradation of *ZNF101* mRNA was faster in cells transfected with miR-541-3p mimics compared with controls, while *APOB* mRNA decay was similar in cells transfected with either miR-541-3p mimics or the control miR mimics (Figure 2G). These studies indicate that miR-541-3p enhances posttranscriptional degradation of *ZNF101* mRNA, and that there is a temporal delay in the regulation of *APOB* mRNA. Thus, miR-541-3p likely interacts with the 3′-UTR of *ZNF101* mRNA in Ago2 complexes to induce posttranscriptional degradation.

Next, we asked how Znf101 regulates apoB. A potential Znf101 binding site in the *APOB* promoter exists at +418 bp after the transcription start site (Supplemental Figure 3G). Luciferase activity under the control of the *APOB* promoter decreased after the expression of miR-541-3p mimics, but increased with antimiR-541-3p expression (Figure 2H). Furthermore, siZnf101 reduced *APOB* promoter luciferase activity (Figure 2I), but not when the Znf101 binding site was mutated, indicating that Znf101 is an enhancer of *APOB*.

### MiR-541-3p mimics increase apoA1 production by reducing Casz1 expression.

**Figure 3:**
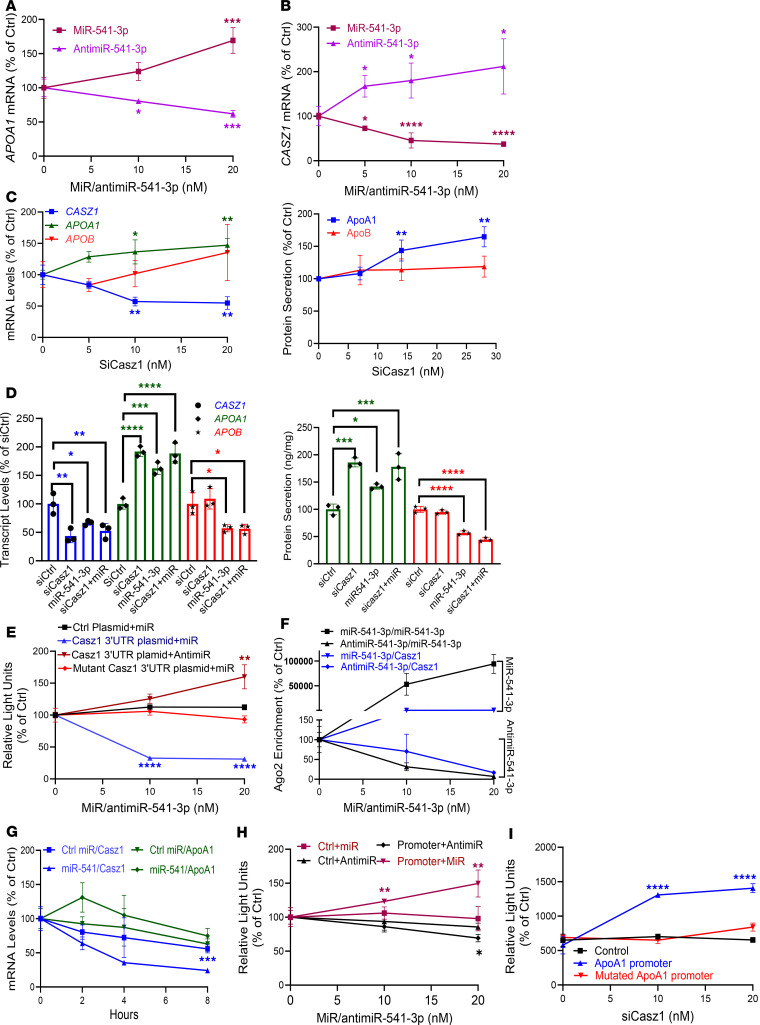
Regulation of apoA1 by miR-541-3p. (**A** and **B**) Huh-7 cells were forward transfected (*n* = 3) with miR-541-3p mimics or antimiR-541-3p. After 48 hours, mRNA levels of *APOA1* and *CASZ1* were quantified in triplicate. (**C**) SiCasz1-transfected cells (*n* = 3) were collected to measure mRNA (left) and media to quantify apoB and apoA1 protein levels (right) in triplicate. (**D**) Cells were transfected (*n* = 3) with 10 nM siCtrl or siCasz1 or 21 nM miR-541-3p mimics, individually or in combination. Cells were used to measure different mRNAs (left) and media to measure apoB and apoA1 protein (right) in triplicate. (**E**) Plasmids for the expression of luciferase with or without the 3′-UTR of Casz1 were transfected (*n* = 3). After 24 hours, cells were reversed transfected (*n* = 3) with different amounts of miR-541-3p mimics or antimiR-541-3p. After 24 hours, luciferase activity was measured in triplicate in media. (**F**) Cells were transfected (*n* = 3) with increasing amounts of miR-541-3p mimics or antimiR-541-3p. After 48 hours, Ago2 immunoprecipitates were used to quantify miR-541-3p and *CASZ1* mRNA levels in triplicate and expressed as a percentage of control miR–transfected cells. (**G**) Transfected cells were treated with actinomycin D (10 μg/mL) in triplicate and collected at different intervals to measure *CASZ1* and *APOA1* mRNA levels. (**H**) Cells were transfected (*n* = 3) with apoA1 promoter–luciferase constructs. The next day, they were reverse transfected (*n* = 3) with increasing concentrations of miR-541-3p mimics or antimiR-541-3p. After 48 hours, luciferase activity was measured in triplicate. (**I**) Cells were transfected in triplicate with plasmids expressing luciferase under the control of the wild-type or mutated apoA1 promoter. The next day, cells were equally distributed and transfected (*n* = 3) with different amounts of siCasz1. After 48 hours, luciferase activity was measured (*n* = 3) in conditioned media. *P < 0.05; **P < 0.01, ***P < 0.001; ****P < 0.0001 by ordinary 1-way ANOVA pairwise multiple comparisons, where different groups were compared with control.

To understand regulation of apoA1 by miR-541-3p, we used a similar approach to that outlined above for apoB. Transfection of increasing concentrations of miR-541-3p mimics enhanced, while antimiR-541-3p decreased, *APOA1* mRNA levels, respectively (Figure 3A). *APOA1* mRNA is not a target of miR-541-3p and BLAST revealed no complementarity between miR-541-3p and the *APOA1* mRNA and promoter. Thus, Watson-Crick base pairing between miR-541-3p and *APOA1* mRNA is unlikely. Subsequently, we identified TFs that might regulate *APOA1*, contain miR-541-3p target sites in their 3′-UTRs, and are expressed in Huh-7 cells (Supplemental Figure 4, A and B). Our studies found that transcript levels of *CASZ1* were significantly decreased following overexpression of miR-541-3p mimics, while antimiR-541-3p had the opposite effect on *CASZ1* mRNA (Figure 3B and Supplemental Figure 4C). Transfection of siCASZ1 significantly reduced *CASZ1* and increased *APOA1* mRNA expression and protein secretion, without affecting *APOB* transcript or protein levels (Figure 3C). Thus, miR-541-3p may reduce Casz1 to enhance apoA1 expression.

We then attempted to learn how miR-541-3p regulates Casz1. MiR-541-3p mimics and siCASZ1, either individually or combined, decreased *CASZ1* and increased *APOA1* mRNA levels and protein secretion, to similar extents (Figure 3D and Supplemental Figure 4D), indicating they are in the same pathway. TargetScan predicted that *CASZ1* mRNA contains a miR-541-3p–interacting site in its 3′-UTR that is conserved in primates (Supplemental Figure 4E). MiR-541-3p mimics decreased luciferase activity when expressed with the 3′-UTR of *CASZ1* mRNA, whereas antimiR-541-3p increased luciferase activity (Figure 3E). These effects were abolished after mutagenesis of the 3′-UTR of *CASZ1* mRNA (Figure 3E and Supplemental Figure 4F). Higher miR-541-3p and *CASZ1* mRNA levels were found in Ago2 precipitates from miR-541-3p mimic–transfected cells, whereas cells transfected with antimiR-541-3p had lower miR-541-3p and *CASZ1* mRNA (Figure 3F). *CASZ1*, but not *APOA1*, mRNA was degraded faster in miR-541-3p mimic–expressing cells compared with control cells (Figure 3G). These studies indicated that miR-541-3p likely interacts with the 3′-UTR in Ago2 complexes and induces posttranscriptional degradation of *CASZ1* mRNA.

We then investigated how Casz1 increases apoA1 expression. A potential Casz1 binding site is located at –789 bp from the transcription start site in the *APOA1* promoter (Supplemental Figure 4G). MiR-541-3p mimics increased, while antimiR-541-3p decreased, luciferase activity under the control of the *APOA1* promoter (Figure 3H). Furthermore, siCASZ1 significantly increased *APOA1* promoter activity, and this increased activity was abolished following mutagenesis of the Casz1 binding site in the *APOA1* promoter (Figure 3I). These studies indicate that Casz1 is a repressor of apoA1 expression.

The above studies identified *MIR541*, *ZNF101*, and *CASZ1* genes as modulators of apoB and apoA1 in human liver cells. To extend the relevance of these genes to humans, we screened for genetic associations of variants in these gene loci with plasma lipids in the UK Biobank (https://www.ukbiobank.ac.uk/). We found significant associations between rs7161194, located approximately 1.8 kb upstream of *MIR541*, and higher plasma HDL-cholesterol (HDL-C) levels; rs34071855, located in the second intron of *CASZ1*, and reduced plasma cholesterol, LDL-C, and apoB levels; and rs2304130, located in the second intron of *ZNF101*, and reduced plasma cholesterol, LDL-C, apoB, and triglyceride levels (Supplemental Figure 5, A–C). These associations were further replicated in the Global Lipid Genetic Consortium (GLGC) dataset (16) (Supplemental Figure 5, D and E). The observed significant associations suggest a possible role for these genes in regulating human plasma lipid and lipoprotein levels.

### Mouse orthologs of human ZNF101 and CASZ1 genes.

To evaluate in vivo roles of *MIR541*, *ZNF101*, and *CASZ1*, we searched for their mouse orthologs. Comparison of human and mouse miR-541-3p sequences revealed 3 differences each in seed and non-seed sequences (Supplemental Figure 6A). Furthermore, predicted mRNA targets for mouse and human miR-541-3p are different (not shown). Thus, hsa-miR-541-3p and mmu-miR-541-3p have different seed sequences and are not functional orthologs.

Mouse *Casz1* is an ortholog of human *CASZ1* (GeneCards). KD of Casz1 in mouse AML12 hepatocytes significantly increased *Apoa1* expression (Supplemental Figure 5B). Mice have 2 orthologs, *Zfp101* and *Zfp961*, of human *ZNF101*. The KD of *Zfp101* had no effect on *Apob* mRNA. However, siZfp961 significantly reduced *Zfp961* and *Apob* mRNA levels (Supplemental Figure 6B). Thus, mouse *Casz1* and *Zfp961* genes are functional orthologs of human *CASZ1* and *ZNF101* with respect to *Apoa1* and *Apob* gene regulation. Since high-fat diets affect plasma lipids and atherosclerosis, we next asked whether hepatic Casz1 and Zfp961 are regulated in vivo by these diets. C57BL/6J mice were fed chow, Western, or obesogenic diets. High-fat diets increased *Zfp961* mRNA levels, but had no effect on *Casz1* expression (Supplemental Figure 6C).

### Hepatic KD of Casz1 and Zfp961 alters plasma apoB-Lps and HDL.

**Figure 4:**
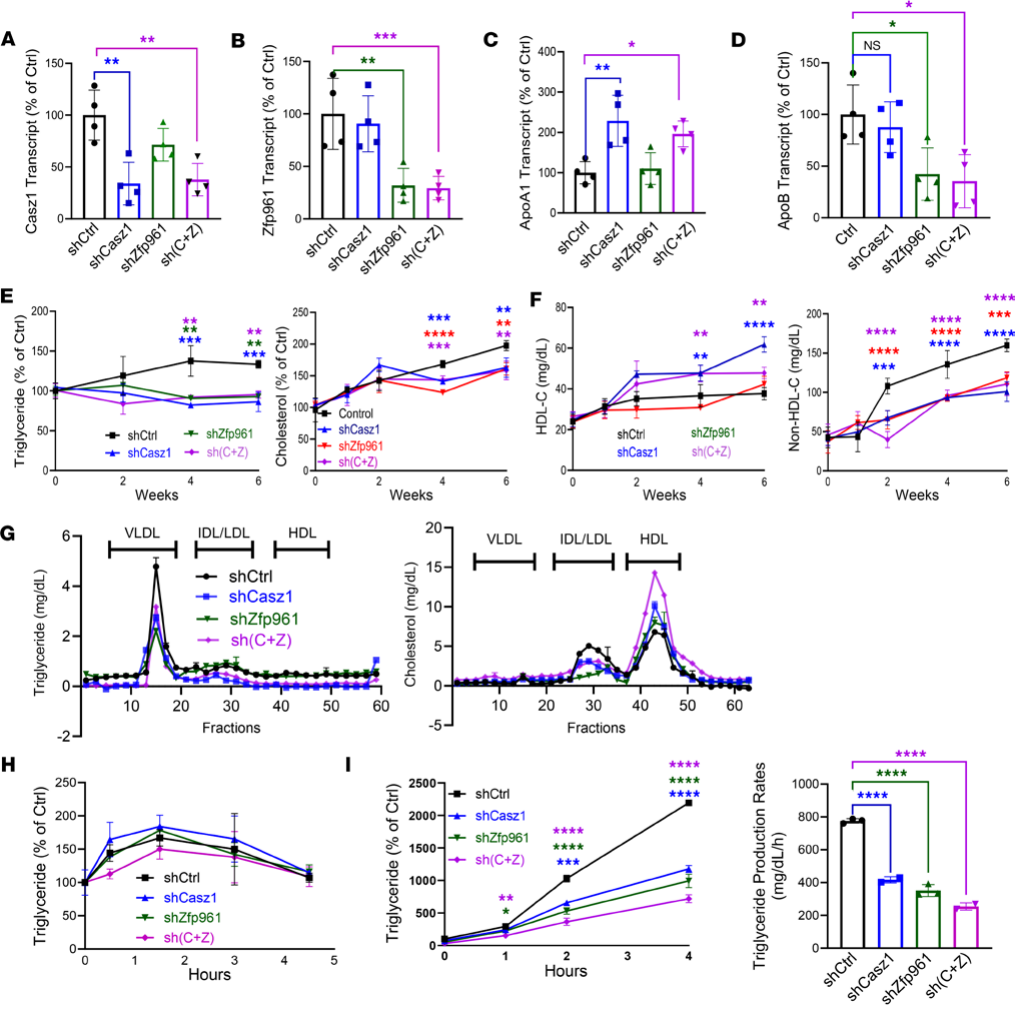
Hepatic KD of Casz1 and Zfp961 alters plasma lipoproteins. Mice (C57BL/6J, female, 2.5 months old) were transduced with AAV8 (2.5 × 10^11^ gc/mouse) expressing shControl (shCtrl, *n* = 4), shCasz1 (*n* = 4), shZfp961 (*n* = 4), or [sh(C+Z), *n* = 5], and fed a Western diet. (**A**–**D**) After 6 weeks, livers were collected to measure mRNA levels in triplicate. (**E** and **F**) Plasma was collected from fasting mice to measure triglyceride and cholesterol (**E**) in technical duplicate, and subjected to precipitation using polyethylene glycol to measure cholesterol in HDL and non-HDL fractions (**F**) in triplicate. (**G**) Plasma (200 μL) obtained at the end of the study was subjected to FPLC, and triglyceride and cholesterol were measured in different fractions. (**H**) Mice were gavaged with olive oil (100 μL) after overnight fasting. Plasma was collected at different times to measure triglycerides in duplicate. Increases in plasma triglyceride were plotted as percentage of initial values before gavage. (**I**) Mice were fasted overnight prior to blood collection. Mice were injected intraperitoneally with poloxamer 407 (1.5 mg/g). Blood was collected to measure triglycerides (left) in triplicate. Data between 2 and 4 hours were used to calculate production rates (right). *P < 0.05; **P < 0.01, ***P < 0.001; ****P < 0.0001 by ordinary 1-way ANOVA pairwise multiple comparisons, where different groups were compared with control.

To test whether mouse Casz1 or Zfp961 regulate hepatic apoB and apoA1 levels, we transduced adult C57BL/6J mice with adeno-associated viruses (AAVs) expressing shRNAs against both TFs, either alone or together, and fed them a high-fat, high-cholesterol Western diet. Both shCasz1 and shZfp961 specifically reduced hepatic *Casz1* and *Zfp961* mRNA, respectively, establishing their specificities (Figure 4, A and B). Hepatic *Apoa1* mRNA levels increased (~2.3-fold) in shCasz1-transduced mice, but remained undisturbed in shZfp961-transduced mice (Figure 4C). *Apob* transcript levels did not change in shCasz1-transduced mice, but were significantly reduced (~60%) in shZfp961-transduced mice (Figure 4D). KD of Casz1 and Zfp961 had no effect on hepatic *Ppara*, *Pgc1a*, *Cebp*, *Mttp*, *Abca1*, and *Ldlr* mRNA, hepatic triglyceride/cholesterol, and plasma alanine transaminase (ALT) and aspartate transaminase (AST) levels compared with control mice (Supplemental Figure 7, A–C). Similarly, these KDs had no effect on total weight gain, or on other physical and physiological parameters, but they reduced fat mass (Supplemental Figure 7, D and E, and Supplemental Table 2). Thus, reduction of hepatic *Casz1* increases *Apoa1*, whereas *Zfp961* KD decreases *Apob* expression.

We also studied the effects of their hepatic KDs on plasma lipids and lipoproteins. The rise in plasma triglyceride and cholesterol over time was significantly lower in shCasz1- and shZfp961-transduced mice compared with that in shCtrl mice (Figure 4E). HDL-C significantly increased (~63%) in shCasz1- and double shCasz1+shZfp961-transduced mice, but were unaffected by shZfp961 KD alone (Figure 4F). KD of either Zfp961 and Casz1, or both together, however, significantly reduced non–HDL-C (Figure 4F). Most of the triglycerides were in very low-density lipoprotein (VLDL) fractions in control mice, but the levels were reduced in all KD mice compared with controls (Figure 4G). Fast performance liquid chromatography (FPLC) studies further showed that cholesterol was mainly in HDL of control mice. HDL-C was unaffected by shZfp961, but increased in mice transduced with shCasz1 and shCasz1+shZfp961 (Figure 4G), indicating that increases in plasma cholesterol in shCasz1 mice are due to increases in HDL-C. These studies showed that hepatic KD of Casz1 and Zfp961, either separately or together, reduces triglyceride and non–HDL-C, whereas only Casz1 KD increases HDL-C. Importantly, KD of these TFs had no effect on intestinal lipid absorption (Figure 4H), while hepatic triglyceride production rates were significantly reduced (Figure 4I). Our findings suggest that hepatic inhibition of both Casz1 and Zfp961 may reduce plasma lipids and apoB-Lps, as a result of impaired hepatic lipoprotein production.

### Atherosclerosis is reduced following hepatic KD of Casz1 and Zfp961.

**Figure 5:**
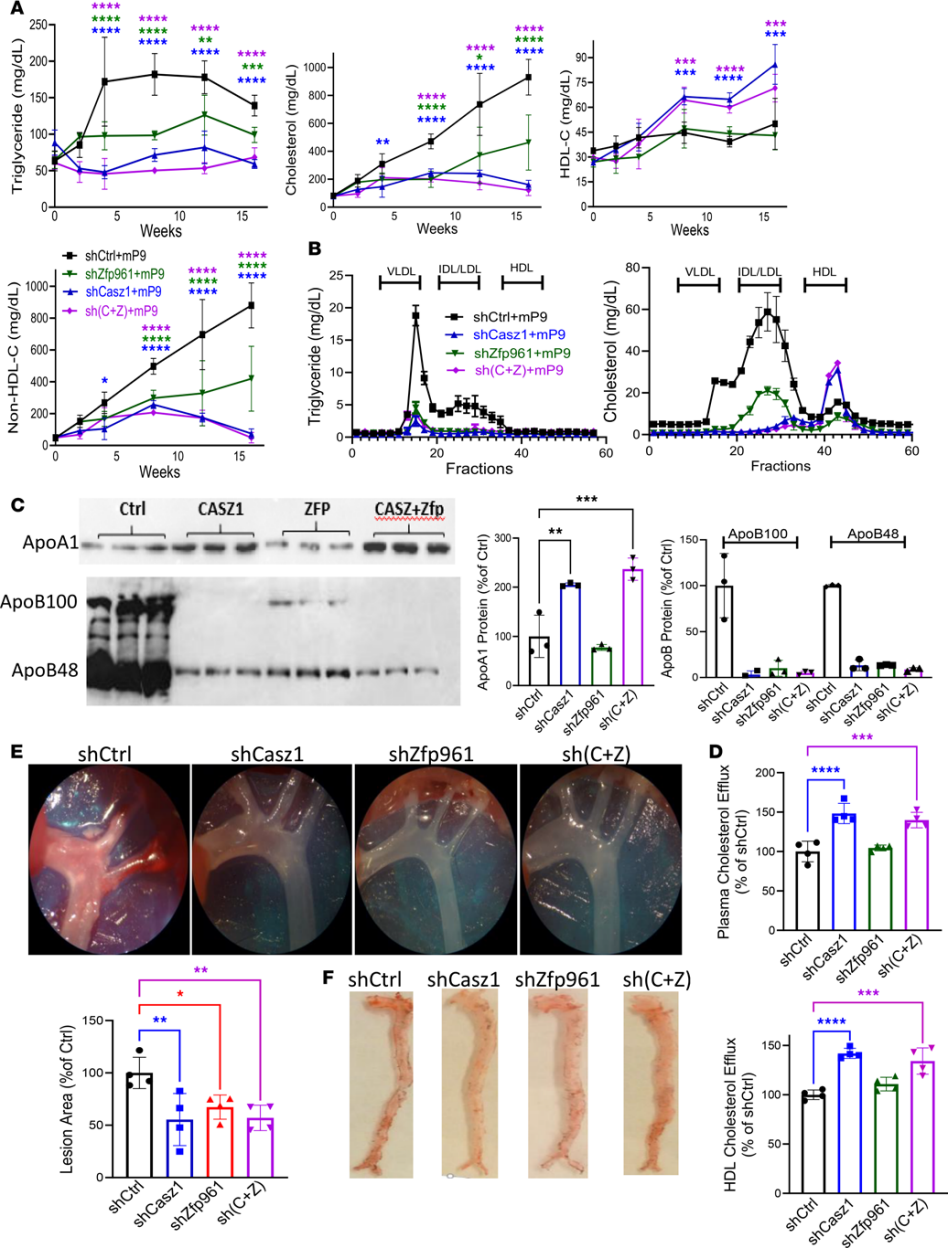
Hepatic KD of Casz1 and Zfp961 in mutant Pcsk9–transduced mice reduces atherosclerosis. All mice (C57BL/6J, male, 2.5 months old) received AAV8 (5 × 10^11^ gc) for the expression of mutant mouse gain-of-function Pcsk9 (mPcsk9). They were divided into 4 groups and received AAV8 for the expression of either shCtrl (5 × 10^11^, *n* = 4), shCasz1 (shCasz1 2.5 × 10^11^ + 2.5 × 10^11^ shCtrl, *n* = 4), shZnf101 (shZnf101 2.5 × 10^11^ + shCtrl, 2.5 × 10^11^, *n* = 4), or shCasz1+shZnf101 (shCasz1, 2.5 × 10^11^ + shZnf101, 2.5 × 10^11^, *n* = 5) at the same time. Mice were fed an atherogenic Western diet. (**A** and **B**) Plasma was collected from overnight fasted mice to measure triglyceride, cholesterol, HDL-C, and non–HDL-C (**A**) in duplicate, and lipoprotein characterization using FPLC (**B**). IDL, intermediate-density lipoprotein. (**C**) Plasma (1 μL) was resolved in gels and probed for apoA1 (top) and apoB (bottom) using specific antibodies. Images were quantified using ImageJ and plotted as percentage of Ctrl. (**D**) Total plasma (10 μL, left) and HDL (20 μg/mL, right) were used in triplicate to study efflux of [^3^H]-cholesterol from J774 macrophages. (**E** and **F**) After 4 months, mice were dissected to visualize plaques in the aortas (**E**). Total aortas were opened and stained with Oil Red O to visualize lipid deposition (**F**). *P < 0.05; **P < 0.01; ***P < 0.001; ****P < 0.0001 by ordinary 1-way ANOVA pairwise multiple comparisons, where different groups were compared with control. Representative of 2 experiments.

To test whether KD of Casz1 and/or Zfp961 could reduce atherosclerosis, we used a mouse atherosclerosis model, in which all adult mice were transduced with mutant mouse gain-of-function Pcsk9 (mPcsk9) (17). mPcsk9 augments degradation of hepatic LDL receptors, increases plasma cholesterol levels, and induces atherosclerosis. ShZfp961 and shCasz1 both specifically reduced their own target mRNAs; while shZfp961 reduced *Apob* mRNA levels without affecting *Apoa1*, shCasz1 increased *Apoa1* without affecting *Apob* mRNA levels (Supplemental Figure 8A). Control mice transduced with mPcsk9+shCtrl showed increased plasma triglyceride, cholesterol, and non–HDL-C (Figure 5A). Mice transduced with shCasz1 and shZfp961, on the other hand, had significantly reduced plasma levels of triglyceride, cholesterol, and non–HDL-C, while the shCasz1-transduced mice also had higher HDL-C levels compared with shCtrl (Figure 5A). Triglyceride and cholesterol in VLDL and intermediate-density lipoprotein/LDL fractions were significantly reduced in all KD mice compared with controls (Figure 5B). In contrast, KD of Casz1 significantly increased plasma HDL-C levels. Plasma apoA1 protein levels increased in shCasz1-transduced mice, but they were not affected by shZfp961 (Figure 5C and Supplemental Figure 8B). ApoB100 and apoB48 levels were significantly reduced in all KD mice (Figure 5C and Supplemental Figure 8C). ShCasz1 and shZfp961 had no significant effect on other TFs, lipoprotein genes, hepatic lipids, and plasma transaminases (Supplemental Figure 9, A–C). Thus, KD of Casz1 and Zfp961 reduces plasma cholesterol and triglyceride in apoB-Lps without affecting hepatic lipids, whereas Casz1 KD, in addition, increases HDL-C.

We asked whether increased HDL in Casz1-KD mice is functional and tested its ability to efflux cholesterol from macrophages. Plasma and HDL from shCasz1-transduced mice showed increased cholesterol efflux from J774 macrophages compared with shCtrl (Figure 5D), indicating increased cholesterol efflux capacity.

We then studied the effects of these KDs on physical and physiological parameters by dual-energy x-ray absorptiometry (DEXA) and comprehensive laboratory animal monitoring system (CLAMS), respectively. ShCasz1- and shZfp961-transduced mPcsk9 mice gained less weight and had significantly less fat than control mPcsk9 mice (Supplemental Figure 9D and Supplemental Table 3). ShCasz1 increased energy expenditure and reduced food intake in the dark, but shZfp961 had no effect (Supplemental Figure 9E). In addition, we visualized atherosclerotic plaques at the end. Atherosclerotic plaques were significantly reduced by approximately 40%–50% in all TF-KD mPcsk9 mice compared with controls (Figure 5, E and F, and Supplemental Figure 10, A and B). These studies show that KD of these TFs reduces atherosclerosis.

**Figure 6:**
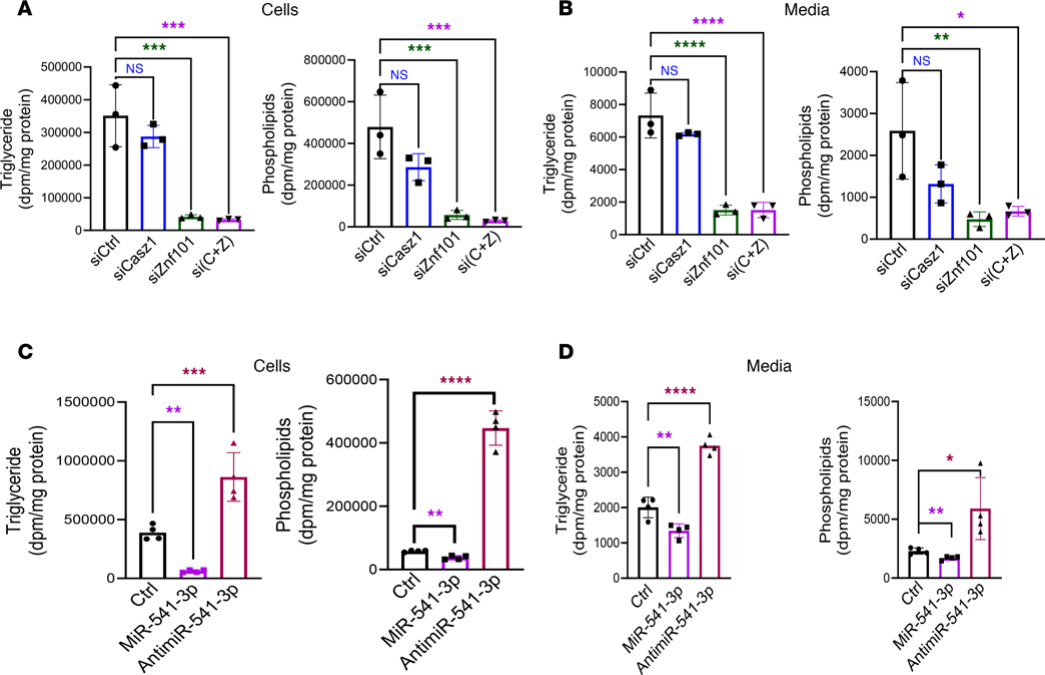
Triglyceride and phospholipid syntheses are reduced in Huh-7 cells overexpressing miR-541-3p and siZnf101. Huh-7 cells were transfected with (**A** and **B**) different siRNAs (10 nM) or (**C** and **D**) miR-541-p or antimiR-541-3p (20 nM) in triplicate. After 24 hours, cells were washed and incubated with [^3^H]-glycerol (5 μCi) for 16 hours. Lipids were extracted from media using CHCl_3_ and CH_3_OH. Cells were washed and incubated with isopropanol for 16 hours at 4°C. Lipids were evaporated, resuspended in isopropanol, and separated on thin-layer chromatography plates. Bands corresponding to triglycerides and phospholipids were excised and counted. *P < 0.05; **P < 0.01; ***P < 0.001; ****P < 0.0001 by ordinary 1-way ANOVA pairwise multiple comparisons, where different groups were compared with control.

The above studies showed that shCasz1 and shZfp961 significantly reduced plasma apoB, but had no significant effect on hepatic lipids and plasma AST/ALT markers that are usually increased after reductions in apoB-Lp assembly. To identify reasons for the absence of hepatosteatosis, we quantified mRNA levels in different pathways. We measured genes in fatty acid oxidation (*Ppara*, *Pgc1a*) and lipoprotein assembly (*Mttp* and *Abca1*) and uptake (LDL receptor). However, we did not see significant differences in their expression (Supplemental Figure 9A). Moreover, we did not observe changes in fatty acid oxidation gene expression (Supplemental Figure 11, A and B); however, mRNA levels of the lipogenic genes *Srebp1c*, *Acc1*, *Scd1*, and glucokinase were significantly reduced (Supplemental Figure 11, C and D). These studies indicated that these genes may play a role in lipid synthesis. This was further tested in Huh-7 cells. KD of ZNF101 significantly reduced synthesis and secretion of triglycerides and phospholipids (Figure 6, A and B). Next we asked whether miR-541-3p also affects lipid synthesis. Expression of miR-541-3p reduced, while antimiR-541-3p enhanced, newly synthesized cellular and secreted triglyceride and phospholipids in Huh-7 cells (Figure 6, C and D). SiCASZ1-treated cells tended to reduce lipid synthesis. These studies showed that KD of these TFs reduces lipid synthesis and this might have prevented hepatosteatosis.

## Discussion

We provide evidence that miR-541-3p concurrently reduces apoB and increases apoA1 in human hepatoma cells by regulating 2 different TFs, Casz1 and Znf101. Mechanistic studies show that miR-541-3p interacts with the 3′-UTR of *CASZ1* and *ZNF101* mRNAs in Ago2 complexes and enhances their degradation. Furthermore, we demonstrate that hepatic KD of orthologous TFs in mice alters the plasma lipoprotein profile and reduces atherosclerosis. Physiological studies showed that hepatic KD of Casz1 augments cholesterol efflux capacity by increasing apoA1 and HDL, whereas hepatic KD of Zfp961 reduces triglyceride production and plasma apoB-Lps. Based on these studies, we speculate that Zfp961/Znf101 and Casz1 can be targeted to modulate plasma LDL and HDL and to reduce atherosclerosis.

It is generally believed that apoB transcription occurs constitutively and that the assembly and secretion of apoB-Lps is controlled by posttranslational mechanisms (18). Here, we show that Znf101/Zfp961 KD reduces apoB mRNA levels in cultured cells and in mice, indicating regulation at the transcriptional level. We found that Znf101 interacts with the *APOB* promoter at +418 to enhance transcription. This site is within the DNase 1–hypersensitive promoter region responsible for apoB expression in liver cells, but not in HeLa cells (19). Furthermore, hepatic KD of Zfp961 reduces apoB mRNA and plasma apoB-Lps, indicating that transcriptional mechanisms can alter plasma lipoproteins. In contrast with Znf101/Zfp961, KD of Casz1 had no significant effect on apoB mRNA levels, but significantly reduced hepatic triglyceride production and plasma apoB levels in mice. These studies suggest that Casz1 might regulate production of apoB-Lps in hepatocytes, involving as of yet unknown posttranscriptional mechanisms.

Several attempts have been made to inhibit hepatic apoB-Lp production to lower plasma lipids and atherosclerosis; however, these ventures have been associated with increased lipid storage in the liver. In this study, reductions in plasma lipoproteins in shZfp961-treated mice were not associated with increases in hepatic lipids. Our studies showed significant decreases in the expression of genes involved in triglyceride and phospholipid syntheses. Furthermore, KD of Znf101 in human liver cells significantly reduced lipid synthesis. These studies suggest that Znf101 might reduce both apoB and lipid synthesis in liver cells to reduce plasma and hepatic lipids.

Although several studies suggest that inhibition of apoB-Lp production causes tissue lipid accumulation (9, 20), there are few examples that show that reductions in apoB-Lp assembly and secretion are not associated with tissue lipid accumulations. Previously, we showed that miR-30c reduces microsomal triglyceride transfer protein (MTP) expression and lipid synthesis to reduce plasma lipoproteins and atherosclerosis without causing hepatic steatosis (14). Differential inhibition of triglyceride and phospholipid transfer activities of MTP reduces hepatic lipoprotein production without causing hepatic steatosis (21). Inhibition of apoB diminishes plasma lipoproteins and increases hepatic degradation of lipids by lipophagy to avoid hepatic lipid accumulation (22, 23). It has been shown that inhibition of DGTA2 and concomitant suppression of de novo lipogenesis helps avoid hepatic lipid accretion (24). Regulated mobilization of fatty acids from adipose tissue (25) and regeneration of adipose tissue in lipodystrophic mice (26) have also been shown to avoid hepatic steatosis. Thus, it is not obligatory that reductions in apoB-Lp production have to be always associated with tissue lipid accumulation. Such knowledge may lead to novel therapeutics that can be combined with commonly used strategies to lower lipids by enhancing catabolism.

A caveat of our studies is that changes in *ZNF101* and *CASZ1* levels were documented via measuring mRNA levels. We have not been able to document changes in their protein levels, as commercially procured antibodies have not yielded specific bands. Another limitation of this study is that we used transient KD approaches to address the roles of Casz1 and Zfp961 in the regulation of plasma lipoproteins and atherosclerosis. Future lipid metabolism and atherosclerosis studies in larger cohorts of liver-specific knockout mouse models will advance knowledge about their role in the regulation of plasma lipoproteins.

In short, we provide evidence that miR-541-3p mimics decrease human apoB and increase apoA1 expression by degrading *ZNF101* and *CASZ1* mRNA in human liver cells. In mouse liver cells, Zfp961 enhances the expression of apoB and plasma LDL, whereas Casz1 reduces the expression of apoA1 and plasma HDL levels. KD of these TFs decreases plasma apoB-Lps, lipogenesis, and atherosclerosis, without causing hepatosteatosis or increasing plasma AST/ALT in mice. Thus, it is likely that Casz1 and Znf101/Zfp961 are potential targets to modulate plasma lipoproteins and reduce atherosclerosis.

## Methods

Further details on reagents, diets used, antibodies, and primers can be found in the Supplemental Methods and Supplemental Table 4.

### Sex as a biological variable.

We have used both male and female C57BL/6J mice from The Jackson Laboratory.

### Cell culture.

Human hepatoma Huh-7 cells (JCRB0403, JCRB Cell Bank), mouse AML12 hepatocytes (CRL-2254, ATCC), and J774 mouse macrophages (TIB-67, ATCC) were cultured in Dulbecco’s modified Eagle medium (DMEM) containing 10% fetal bovine serum (FBS) and 1% L-glutamine in T75 flasks at 37°C in humidified 5% CO_2_ incubators (27, 28).

### Screening.

MiR mimics were suspended in RNase-free water to obtain 2 μM stocks and 3 μL of each miR mimic stock was added in duplicate wells to obtain a final concentration of 50 nM. To each well, we added 7 μL of Opti-MEM and 10 μL of lipofectamine RNAiMAX (Life Technologies) diluted 1:20 in Serum-Reduced Opti-MEM. After 20 to 30 minutes, 25,000 cells in 100 μL of Opti-MEM were added to each well. After an additional 24 hours, culture media were replaced with fresh DMEM containing 10% FBS. Media were changed 24 hours later and cells were incubated with DMEM containing oleic acid/BSA complex (0.4 mM oleic acid/1.5% BSA) for 2 hours. ApoB and apoAI concentrations in medium were measured by ELISA (60). Cells were used for protein measurements.

### Transfection in hepatoma cells.

Huh-7 or AML12 cells (2.5 × 10^5^) were reverse transfected in triplicate in 6-well plates using Endofectin (Genecopoeia) with the indicated doses of miRIDIAN miRNA mimics (miR-541-3p), miRIDIAN miRNA inhibitors (antimiR-541-3p), siRNAs (Origene), scramble control mimic (20 nM, Thermo Fisher Scientific), or siControl as noted in the figure legends. Media were replaced with fresh DMEM containing 10% FBS and 1% L-glutamine after 16 hours of transfection. For long-term cultures, media were changed every 24 hours. In each experiment, cells were transfected with a control miR or miR-541-3p. Similarly, cells treated with antimiR had their own control. In each experiment, control values were normalized to 100%. This allowed us to plot both effects of miRs and antimiRs in a single graph.

### ApoA1 and apoB protein quantifications in Huh-7 cells by ELISA.

One day after transfections, media were replaced with 1 mL of fresh DMEM containing 10% FBS. Cells and media (last 24 hours) were collected 70 hours after transfection. Cells were lysed with 0.1N NaOH to quantify total protein by the BCA method. Total cell protein was used to normalize amounts of the apoA1 and apoB protein in media. To measure apoA1 and apoB protein levels in Huh-7 cells, transfected cells were lysed with 1× RIPA buffer containing protease inhibitor mixture (Sigma-Aldrich). Cell lysates were centrifuged and the supernatants were added to ELISA plates (Costar, Corning) to measure apoA1 (R&D Systems ELISA Kits) and apoB (Mabtech ELISA Kits) in triplicate. In some samples, sandwich ELISA using 1D1 as capture antibodies was used for apoB ELISA in triplicate, as previously described (27).

### Quantification of mRNAs by quantitative RT-PCR.

Huh-7 cells were washed with PBS, 1 mL of TRIzol was added, mixed vigorously for 30 seconds, and incubated for 10 minutes at room temperature. Similarly, 10 mg of mouse liver tissue was lysed in 1 mL of TRIzol and homogenized using a tissue homogenizer. Chloroform (200 μL) was added and mixed vigorously, left at room temperature for 5 minutes, centrifuged (15,700*g*, 15 minutes, 4°C), and the top aqueous phase was transferred to a new tube. Subsequently, samples were mixed with equal volumes of ice-chilled isopropanol and kept at room temperature for 5 minutes. Samples were centrifuged (15,700*g*, 15 minutes, 4°C) and supernatants were discarded, pellets were resuspended in 1 mL of 70% ethanol, centrifuged (12,000 rpm, 15 minutes, 4°C), and supernatants were discarded. Pellets were dried in a 37°C incubator for 10 minutes and dissolved in molecular biology grade water.

First-strand cDNA was synthesized using 1 μg of RNA in triplicate with the Applied Biosystems High-Capacity cDNA Reverse Transcription Kit, which was used for quantitative RT-PCR (PowerTrack SYBR Green Master Mix), and the Ct values for each mRNA were normalized to *18S*. For miR quantification, 100 ng of RNA and miR-specific primers were used for cDNA synthesis using the TaqMan MicroRNA Reverse Transcription kit (Applied Biosystems, 4366597). MiR-541-3p levels were quantified using the Ct method after normalization with *RNU44* or *U6* and are presented as arbitrary units.

### Immunoblotting.

Huh-7 cells (from 1 well of a 6-well plate) were mixed with 200 μL of cold 1× RIPA buffer and vortexed. After complete cell lysis, lysates were centrifuged (15,700*g* for 10 minutes at 4°C). Total protein (25 μg) was resolved in 6% and 12% acrylamide gels for apoB and apoA1, respectively. Liver tissue (50 mg) was homogenized in 1 mL of buffer K (1 mM Tris-Cl, 1 mM EGTA, and 1 mM MgCl_2_, pH 7.6) containing protease inhibitor mixture. Proteins (~25 μg) were resolved in SDS-PAGE gels, transferred to nitrocellulose blotting membranes by the semidry transfer method, and membranes were blocked with 5% dry milk powder in Tris-buffered saline (TBS) for 1 hour. After washing with TBS containing 0.1% Tween 20 detergent (TBST), membranes were incubated with 1:1000 dilution of primary polyclonal antibodies against mouse/human apoA1 and apoB or a rabbit antibody against human/mouse β-actin overnight at 4°C. The next day, membranes were washed and incubated with alkaline phosphatase–conjugated secondary antibody (1:10,000 dilution) for 2 hours, washed, substrate was added, and bands were visualized using a Storm 860 Molecular Imager.

### Luciferase assay.

Plasmids expressing *Gaussia* luciferase with the *ZNF101* 3′-UTR, *CASZ1*-3′ UTR, *APOA1* promoter, or *APOB* promoter (GLuc/ secreted alkaline phosphatase [SEAP] dual-reporter vector system) were obtained from Genecopoeia. Huh-7 cells were first forward transfected with 5 μg of these plasmids. After 16 hours of transfection, cells were trypsinized and equally distributed into wells and reverse transfected in triplicate with miR-541-3p, antimiR-541-3p, or different indicated siRNAs. Luciferase activity was measured in media after 8 hours of reverse transfection using a kit. Luciferase activity was normalized to SEAP activity.

### Ago2 precipitation.

Huh-7 cells transfected (*n* = 3) with miR-541-3p or antimiR-541-3p were collected in buffer K and sonicated to lyse the cells. Cell lysates were centrifuged (9,300*g*, 10 minutes, 4°C), supernatants were collected and incubated for 1 hour with IgG for preclearing. After centrifugation, supernatants were mixed with protein A/G plus agarose beads for 1 hour at 4°C. This mixture was centrifuged (800 rpm, 1 minute) and supernatants were incubated with 1:100 dilution of human Ago2 antibody overnight at 4°C. The next day, mixtures were incubated with agarose beads for 1 hour, and centrifuged (100*g*, 1 minute). Supernatants were discarded and pellets were processed for RNA isolation using TRIzol.

### Actinomycin D study.

After 16 hours of miR-541-3p (20 nM) transfection, Huh-7 cells were treated with 10 μg/mL of actinomycin D in triplicate to analyze posttranscriptional degradation of different mRNAs. Cells were collected every 2 hours and RNA was isolated using TRIzol. Isolated RNAs were used to quantify expression levels of *APOA1*, *APOB*, *CASZ1*, and *ZNF101* transcript levels.

### De novo lipogenesis.

Huh-7 cells (*n* = 4) were reverse transfected with control miR or miR/antimiR-541-3p (20 nM) in 12-well plates. After 8 hours of transfection, cells were washed 3 times with PBS and incubated with 500 μL of serum-free media containing ^3^[H]-glycerol (5 μCi/mL) for 16 hours. Media were collected and processed for lipid isolation using the chloroform/methanol method (29). Cells were washed 3 times with PBS and incubated overnight with 1 mL of isopropanol at 4°C. Isopropanol was collected and dried by vacuum centrifugation. Dried lipids were dissolved in 25 μL of chloroform and applied to thin-layer chromatography (TLC) plates (Whatman, aluminum plates coated with silica, 4420221) and separated using a petroleum ether/diethyl ether/acetic acid (70:30:1) solvent mixture. TLC plates were exposed to iodine vapors for 2–5 minutes, triglyceride and phospholipid bands were marked with pencil, excised, placed in scintillation vials, and 5 mL of ScintiSafe Econo 2 Cocktail was added. Each sample was counted in a liquid scintillation counter (Wallac 1409) for 5 minutes to measure disintegrations per minute (dpm).

### KD of TFs in mice.

Female C57BL/6J mice (*n* = 17, 2.5 months old) were divided into 4 groups and were intravenously transduced with different viruses expressing specific shRNA (2.5 × 10^11^ genome copies [gc]) as follows: shControl (4 mice), shCasz1 (4 mice), shZfp961 (4 mice), and shCasz1+shZfp961 (5 mice). These mice were fed a Western diet (Envigo TD.88137; contains 15.2% protein, 42.7% carbohydrate, 42% fat kcal and 1.5 g/kg cholesterol) for 2 months. In a separate experiment, male mice were transduced with these shRNAs along with an AAV expressing a gain-of-function mutant of mouse Pcsk9 (AAV8-D377Y-mPcsk9, 1.0 × 10^11^ gc) and fed a Western diet for 4 months. Plasma was collected at different times to document changes in plasma cholesterol and triglyceride levels. At the end, mice were fasted overnight, euthanized, and tissues were collected. In these mice, we also evaluated atherosclerosis, by dissecting and examining aortas, as previously described (30).

### Plasma and tissue lipid measurements.

Mice were fasted overnight (15 hours) and blood was collected in EDTA-rinsed tubes from retro-orbital venous plexus. Blood was centrifuged (4,600*g* rpm, 10 minutes), and plasma was collected and stored at –80°C. Total plasma cholesterol and triglyceride concentrations were enzymatically measured in triplicate using kits. HDL-C was measured after precipitating apoB using equal volumes of HDL cholesterol reagent (Pointe Scientific). LDL-C values were determined by subtracting the concentrations of HDL-C from total lipids. For hepatic lipid measurements, liver pieces (~50 mg) were homogenized in 1 mL of buffer K, and 200 μL was subjected to lipid extraction (29, 31). Plasma ALT (Cayman Chemical) and AST (Sigma-Aldrich) were measured in triplicate using kits according to the manufacturers’ instructions.

### FPLC.

Plasma samples (200 μL) were loaded onto an AKTA pure FPLC column (Superose 6 Increase 10/300 GL FPLC column, GE Healthcare). Lipoproteins were eluted with PBS and 0.25 mL fractions were collected at a flow rate of 0.4 mL/min.

### Cholesterol efflux.

Mouse J774 macrophages were seeded in 12-well plates at a density of 75 × 10^3^ cells/well and cultured in humidified incubator (37°C, 5% CO_2_). After 15 hours, 250 μL of complete DMEM containing 0.25 μCi [^3^H]-cholesterol and 5 μg acetylated LDL (Ac-LDL) were added to each well and incubated. After 48 hours, cells were examined under a microscope to ensure healthy appearance and at approximately 80% confluence, media were removed and cells were washed 3 times with PBS. Next, 500 μL of Opti-MEM containing 2 μM LXR agonist T0-901317 was added into each well. After 18 hours, media were removed and cells were gently washed with PBS. Next, Opti-MEM (500 μL) containing acceptor (HDL-C, 5 μg) or (plasma, 10 μL) in triplicate was added into each well. One set of wells received Opti-MEM only for background. After 4 hours, different media were collected and centrifuged at 15,700*g* for 10 minutes. Plates were kept in the freezer for 1 hour, prior to adding 500 μL dH_2_O to each well, and incubated on a shaker at 4°C for 1 hour to detach cells. Cells were pipetted up and down to break up cell clumps and obtain homogeneous suspensions. Media (100 μL) and cells (100 μL) were transferred to 7 mL scintillation vials, 5 mL of Insta-gel Plus (PerkinElmer) was added, mixed well, and ^3^H dpm was measured in a scintillation counter. The following formula was used to measure cholesterol efflux from cells to the acceptor: cholesterol efflux (%) = (media count – background)/(media count – background) + (cell counts – background) × 100.

### Hepatic triglyceride production.

Mice were fasted for 16 hours and then 1.5 mg/g (in 500 μL PBS) of poloxamer 407 was injected intraperitoneally. Plasma was collected at the indicated time points. Triglycerides were measured using a kit. Rates of triglyceride production were measured between 2 and 4 hours.

### Intestinal lipid absorption studies.

Mice were fasted overnight, and then 100 μL of olive oil was administered by intragastric gavage. Blood samples were collected from retro-orbital venous plexus at different times. Plasma triglyceride levels were measured using kits.

### Aortic plaque analyses.

Aortic arches were dissected and exposed for photography and quantified with ImageJ (NIH). Aortic fatty streaks were visualized with Oil Red O staining.

### Statistics.

All data are presented as mean ± SD. Statistical significance (*P* < 0.05) was determined using an unpaired *t* test or ordinary 1-way ANOVA pairwise multiple comparisons, where different group were compared with control.

### Study approval.

The animal studies were approved by the IAUCUC at NYU Grosmann Long Island School of Medicine.

### Data availability.

All raw data supporting the conclusions of this study are included in the Supporting Data Values file.

## Author contributions

MMH conceived the ideas, supervised experiments, and secured funding. LZ performed the initial screening of the miR library. BP performed Oil Red O and trichrome staining of the aortic arch cross sections as well as Western blots for MTP and ABCA1. LI and AC performed several secondary screenings. PKY focused work on miR-541-3p. AA took over the project and performed all the cell culture and mechanistic studies and brought the studies to a fruitful conclusion. PKY and AA performed all the mouse experiments. SA worked with AA in quantifying mRNA levels. AR performed genetic analyses. AA made the figures and wrote the first draft of the manuscript, which was extensively modified by MMH. All authors read the drafts and provided their suggestions on the manuscript.

## Supplementary Material

Supplemental data

Unedited blot and gel images

Supporting data values

## Version 1 01/09/2025

Electronic publication
