# Supplementary material for: Casz1 and Znf101/Zfp961 differentially regulate apolipoproteins A1 and B, alter plasma lipoproteins, and reduce atherosclerosis
Source: JCI Insight. 2025 Jan 9;10(1):e182260. doi: 10.1172/jci.insight.182260 (PMC11721306; doi:10.1172/jci.insight.182260)
Supplement: Supplemental data [file jciinsight-10-182260-s138.pdf]

Supplementary Fig 1

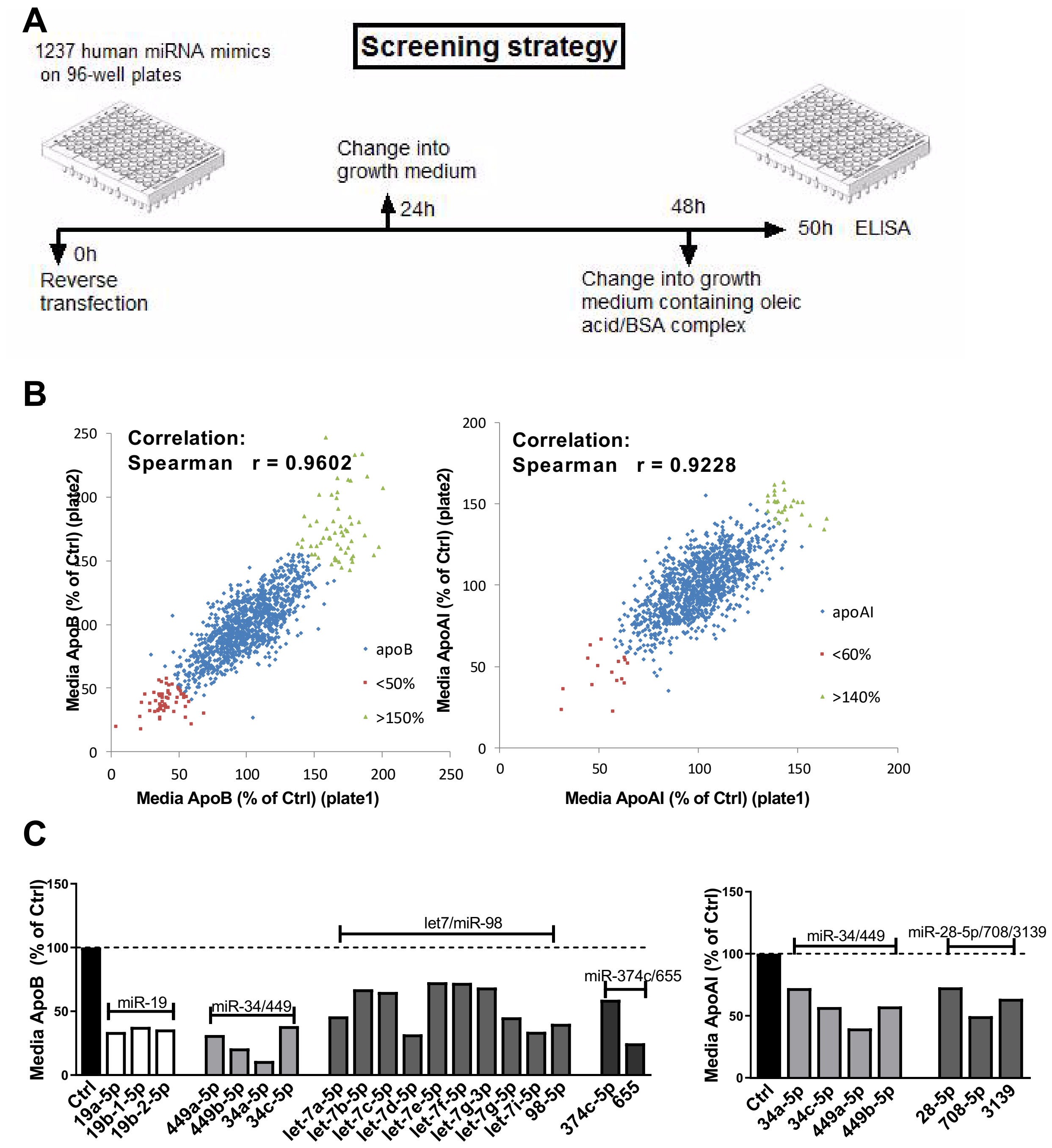

Supplementary Fig 1. Screening a library to identify microRNAs regulating apoB and apoA1 secretion in human hepatoma Huh-7 cells.

(A) Huh-7 cells were reverse transfected in duplicate plates with a human miRDIAN mimic 16.0 library (Dharmacon) of 1237 miRs at 50 nM. After 24 h, cells received complete media with 10% FBS. After another 24 h, cells were incubated with complete media containing 10% FBS and oleic acid/BSA complexes (0.4 mM/1.5%) for 2 h to avoid identification of miRs that affect posttranslational degradation of apoB. Media were used to quantify apoB and apoA1 levels by ELISA. Few wells in each plate were simultaneously transfected with negative control (Ctrl) or miR-30c (positive control, reduces apoB).

(B) Percentage change in media apoB and apoA1 in two plates exposed to the same miRs were compared with the negative control. Changes (%) in plate 1 are plotted against plate 2. Correlation between two plates with respect to changes in media apoB and apoA1 was determined.

(C) Different miR family members with the same seed sequence showed similar reductions in media apoB and apoA1 indicating internal consistency in the regulation of apoB and apoA1 secretion by family members.

Supplementary Fig 2

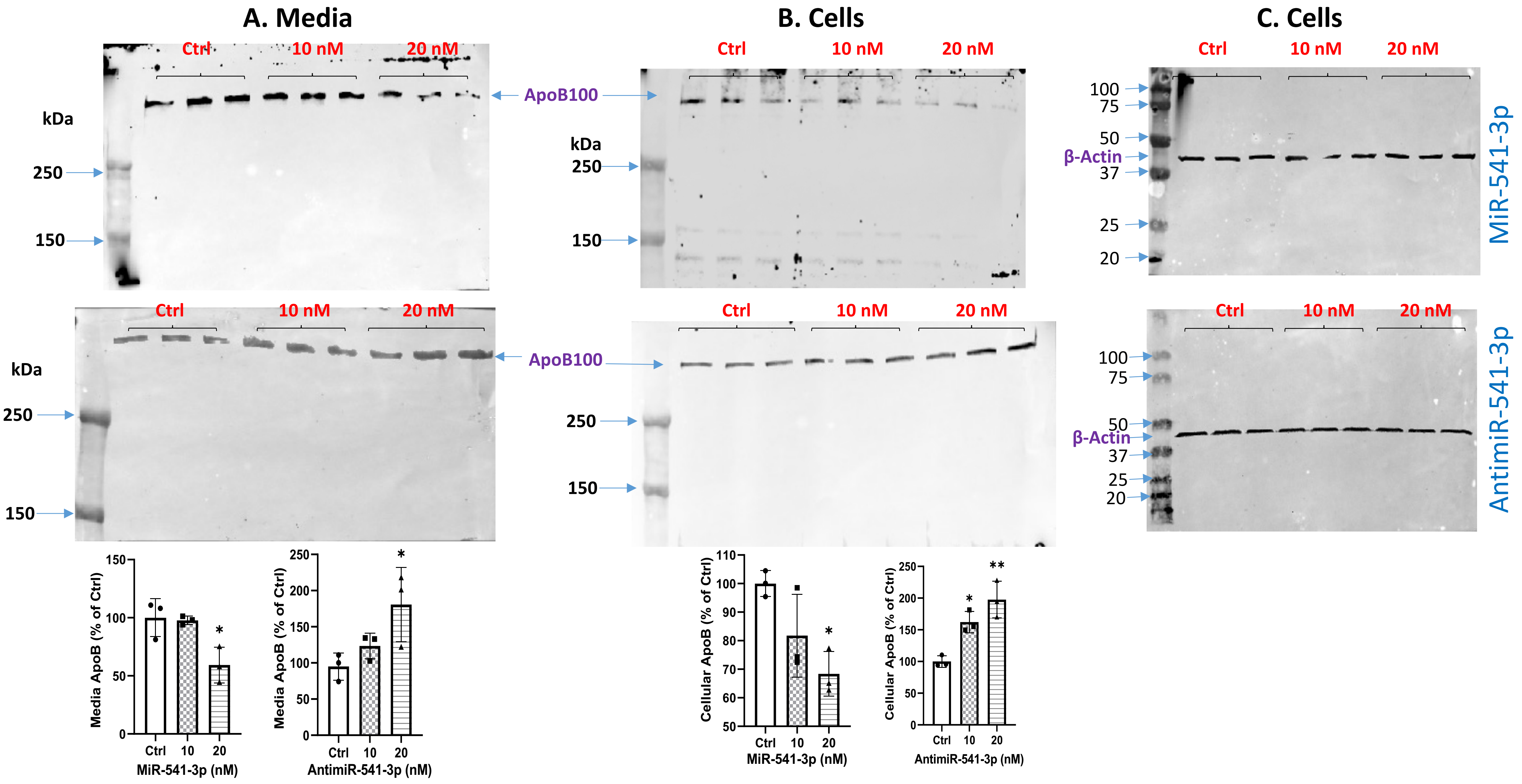

Supplementary Fig 2

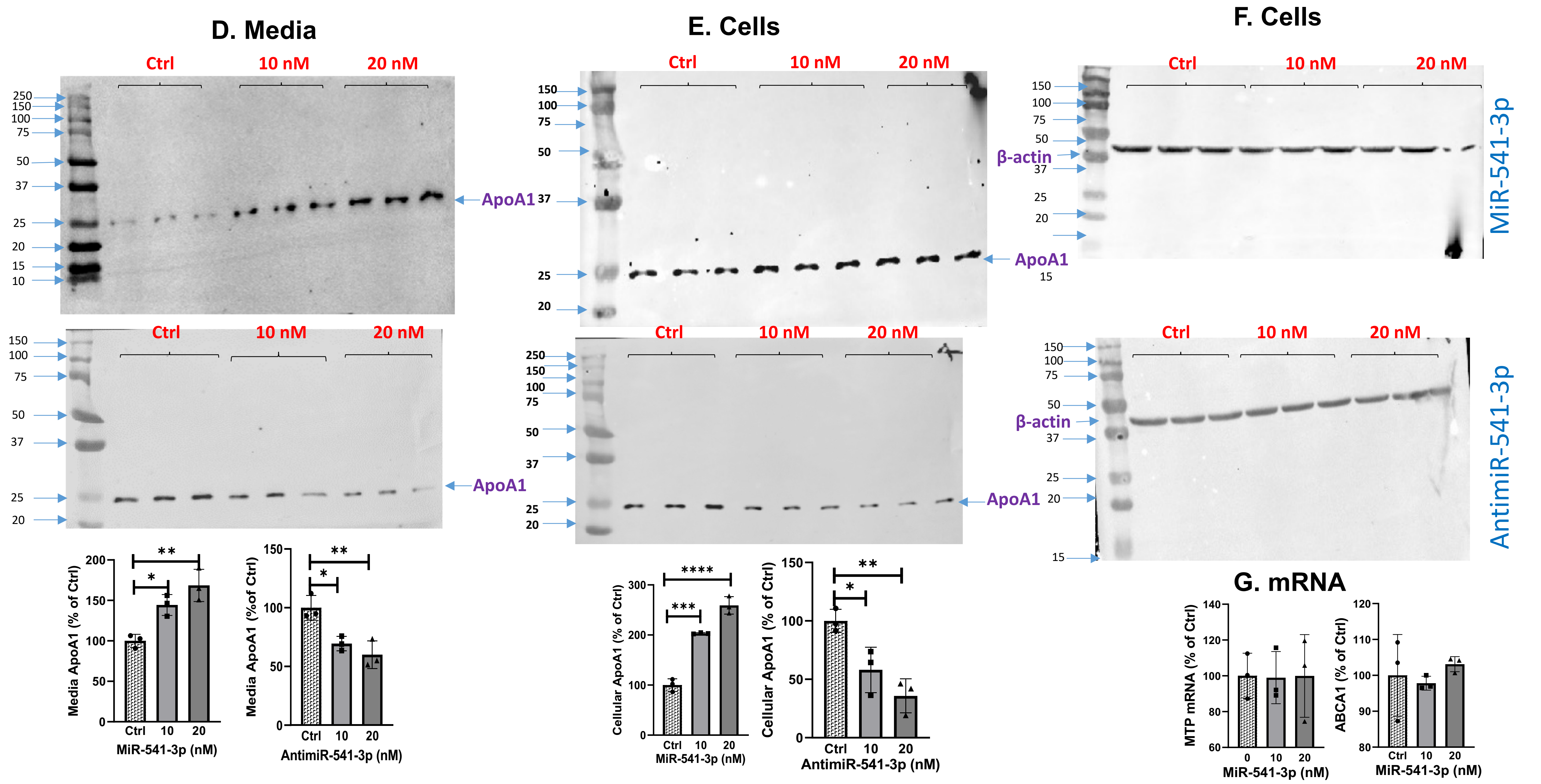

Supplementary Fig 2. Regulation of apoB and apoA1 by miR-541-3p.

(**A-C**) Full gels probing apoB100 in the (A) media and (B-C) cell lysates of Huh-7 cells treated with miR-541-3p mimics (top) or antimiR-541-3p (bottom), respectively. Bands were quantified by ImageJ and plotted as % of control (bottom). The densitometric analysis showed significant reductions in apoB protein levels in cells and media of Huh-7 cells overexpressing miR-541-3p mimics and significant increases in cells overexpressing antimiR-541-3p. MiR-541-3p mimics and antimiR-541-3p had no effect on β-actin protein levels.

(**D-F**) Full gels and quantification of apoA1 bands in media (A) and cells (B) transfected with miR-541-3p mimics and antimiR-541-3p. Protein bands were quantified using ImageJ and normalized to β-actin and plotted as % of control (bottom). MiR-541-3p mimics increase whereas antimiR-541-3p decreases apoA1 expression.

(**G**) Quantification of *MTP* and *ABCA1* mRNA levels in Huh-7 cells transfected with different amounts of miR-541-3p mimics.

Supplementary Fig 3

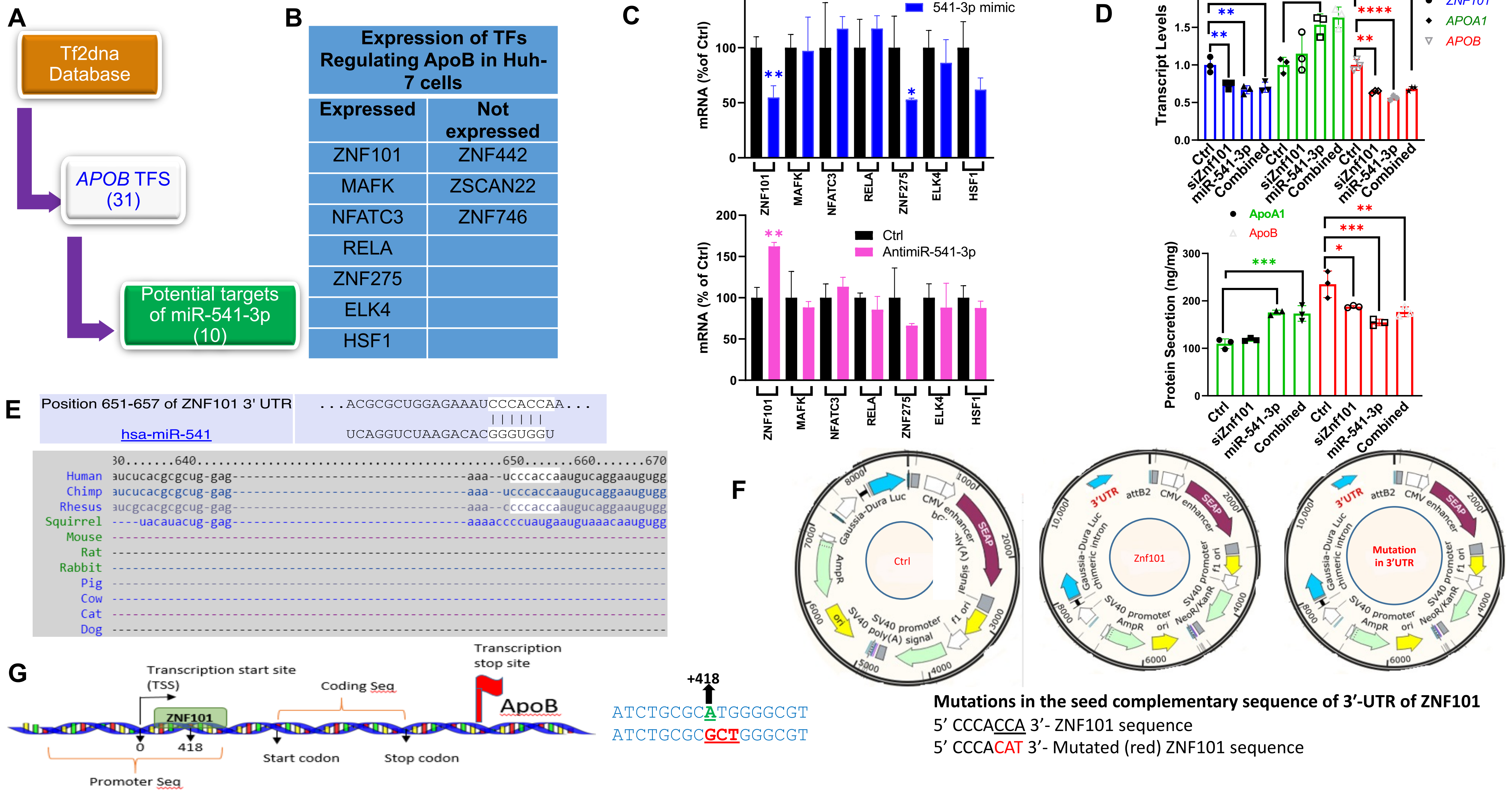

Supplementary Fig 3. Identification of transcription factors (TFs) regulating apoB.

(**A**) Tf2dna database was queried for TFs that could potentially regulate *APOB* gene expression. This identified 31 potential TFs that could regulate *APOB* expression. Ten of these TFs were identified as targets of miR-541-3p using Target Scan.

(**B**) Transcript levels of these 10 TFs were quantified in triplicate using specific primers. Seven TFs could be measured in Huh-7 cells.

(**C**) Huh-7 cells were transfected in triplicate with 20 nM miR-541-3p or antimiR-541-3p. After 48 h, changes in mRNA levels were quantified. Znf101 significantly reduced or increased, respectively, in cells transfected with miR-541-3p mimics or antimiR-541-3p.

(**D**) Huh-7 cells were transfected with miR-541-3p mimics (20 nM) or siZnf101 (28 nM), alone or in combination. After 48 h, cell lysates were used to measure different mRNA levels (left). Conditioned media was used to quantify apoB and apoA1 levels by ELISA (right).

(**E**) Target Scan predicted that the 3′-UTR of ZNF101 mRNA contains complementary bases that could pair with miR-541-3p seed sequence (top). Target Scan was used to determine the conservation of Znf101 3′-UTR in different species. Conserved sequences were found in the rhesus monkey and chimpanzee (bottom).

(**F**) Schematic diagrams of plasmids expressing Gaussia-Dura luciferase under the control of Znf101 3′-UTR obtained from Genecopoeia. The plasmid also constitutively expresses alkaline phosphatase under the control of cytomegalovirus (CMV) promoter and is used as a control. The highlighted bases were mutated in the wild-type plasmid.

(**G**) Schematic diagram showing potential Znf101 binding site in the *APOB* promoter. Transcription start site (TSS) and stop sites are identified. *APOB* promoter and coding sequences (seq) are shown (not to scale). Potential Znf101 binding site was mutated as shown in red.

Supplementary Fig 4

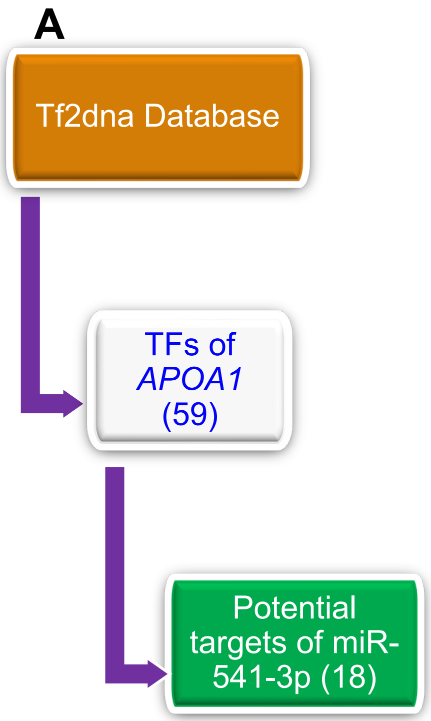

B

Expression of TFs regulating ApoA1 in Huh-7 cells	
Expressed	Not expressed
HNF4A	SP6
ZBTB4	ZNF81
IRF3	FOXI1
ZNF100	ONECUT3
THRB	ZNF418
RARA	
NR4A3	
ZSCAN16	
ZNF726	
ZNF471	
YY1	
CASZ1	
ZNF771	

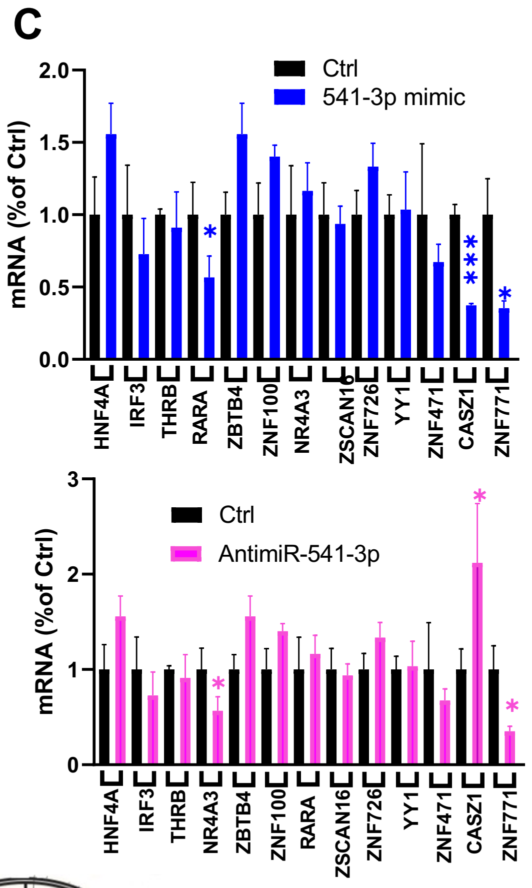

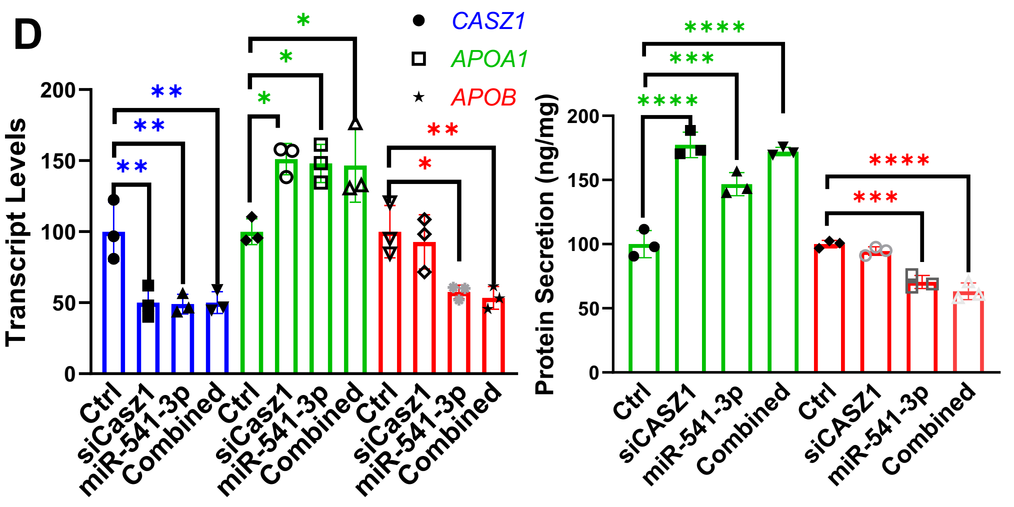

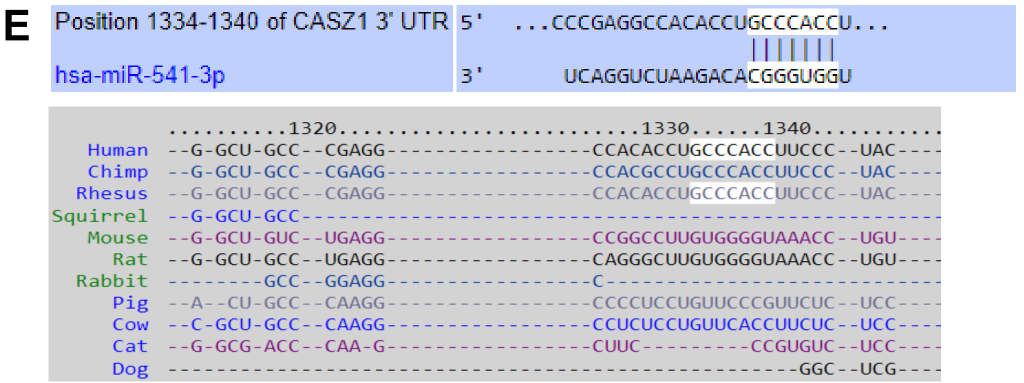

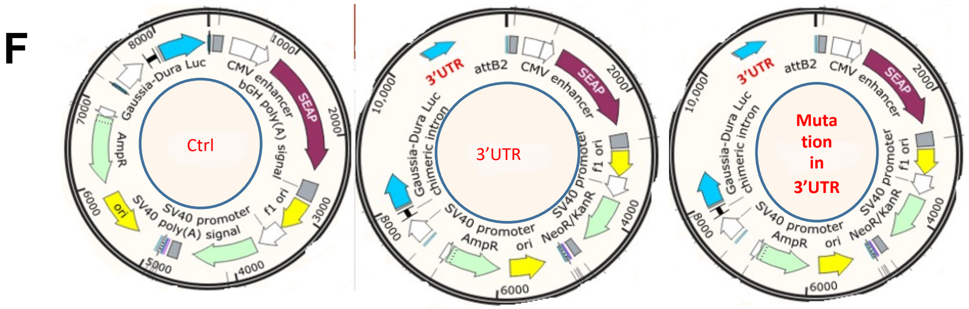

Mutations in the seed complementary sequence 3'-UTR of Casz1:

5' GCCCACC 3'- CASZ1 sequence

5' GCAATCC 3' Mutated (red) CASZ1 sequence

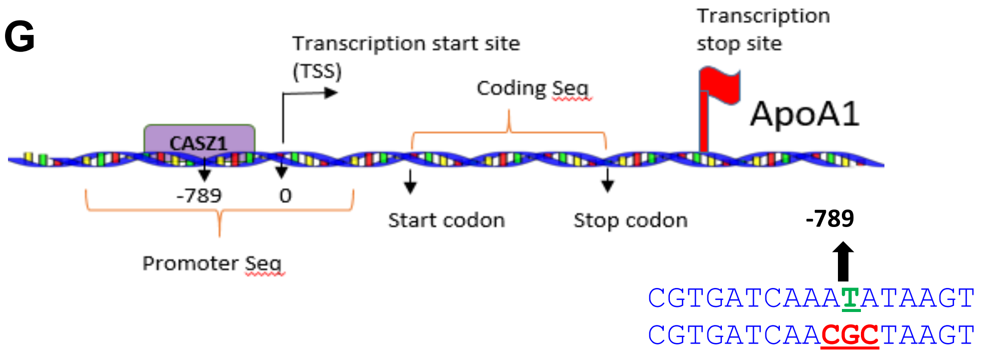

Supplementary Fig 4. Identification of TFs regulating apoA1.

(**A**) Tf2dna database was searched for TFs that could regulate *APOA1* gene expression. This identified 59 potential TFs. Target Scan predicted 18 of these TFs to be targets of miR-541-3p.

(**B**) mRNA levels of these TFs were quantified in triplicate by qRT-PCR. Huh-7 cells express 13 of these TFs.

(**C**) Huh-7 cells were transfected with 20 nM miR-541-3p mimics or antimiR-541-3p. After 48 h, changes in mRNA levels of different TFs were quantified in triplicate. Casz1 was significantly decreased and increased, respectively, in cells expressing miR-541-3p mimics or antimiR-541-3p.

(**D**) Huh-7 cells were transfected in triplicate with miR-541-3p mimics (20 nM) or siCasz1 (28 nM), alone or in combination. After 48 h, mRNA levels were quantified in cells (left), and protein levels in conditioned media (right) in triplicate. MiR-541-3p mimics and siCasz1, individually and combined, increased apoA1 levels to a similar extent indicating that they are in the same pathway.

(**E**) Target Scan 7.2 showed that Casz1 3′-UTR contains a complementary sequence that could base pair with miR-541-3p seed sequence (top). Clustal W alignment algorithms indicated conservation of the miR-541-3p complementary binding sequence in the 3′-UTR of Casz1 in primates (bottom).

(**F**) Plasmids expressing luciferase with Casz1 3′-UTR were obtained from Genecopoeia. Casz1 3′-UTR was mutated as shown below in red.

(**G**) Schematic diagram showing potential Casz1 binding site in the *APOA1* promoter (not to scale). Potential binding site sequence was mutated as shown in red.

Supplementary Fig 5

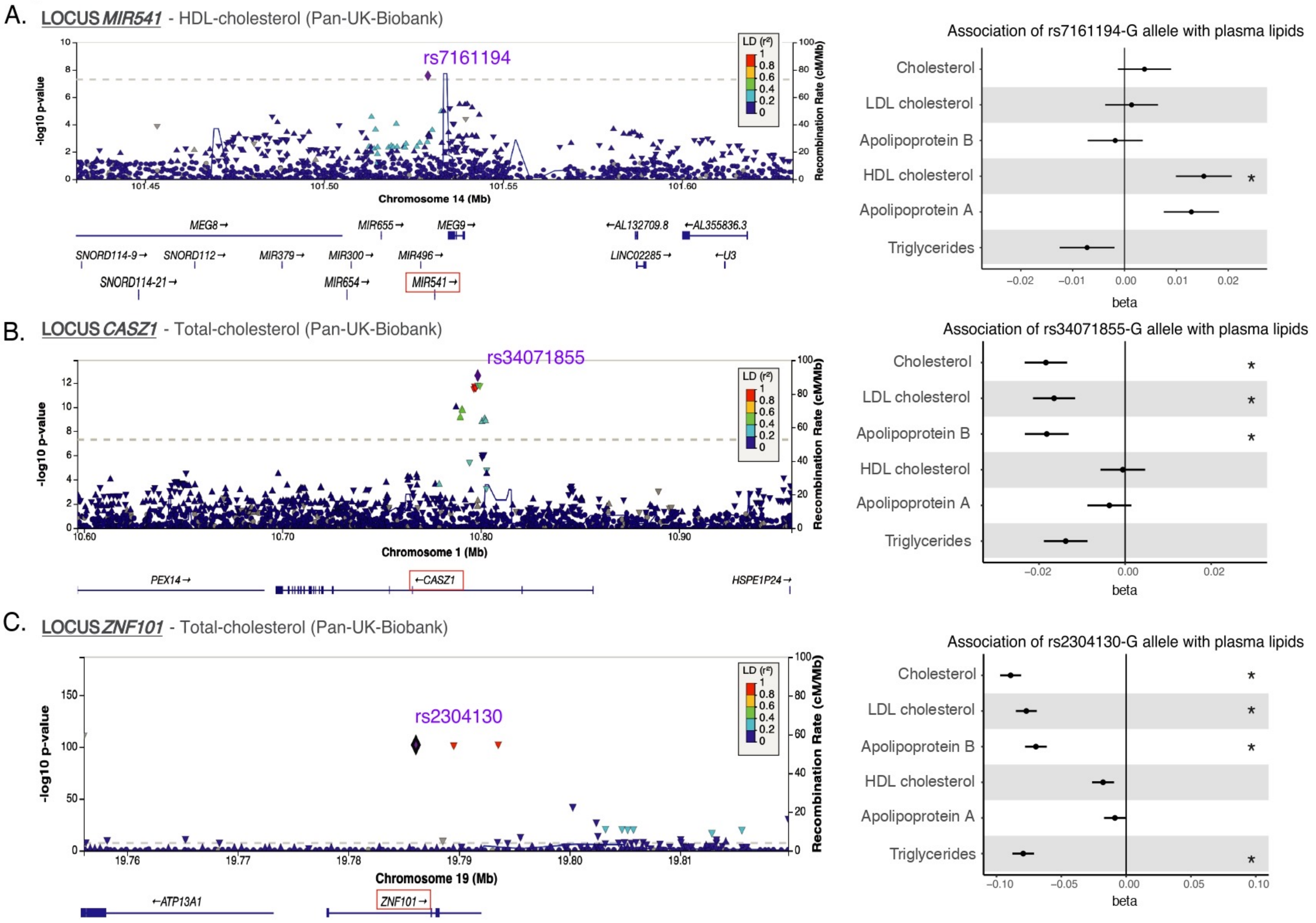

Supplementary Fig 5

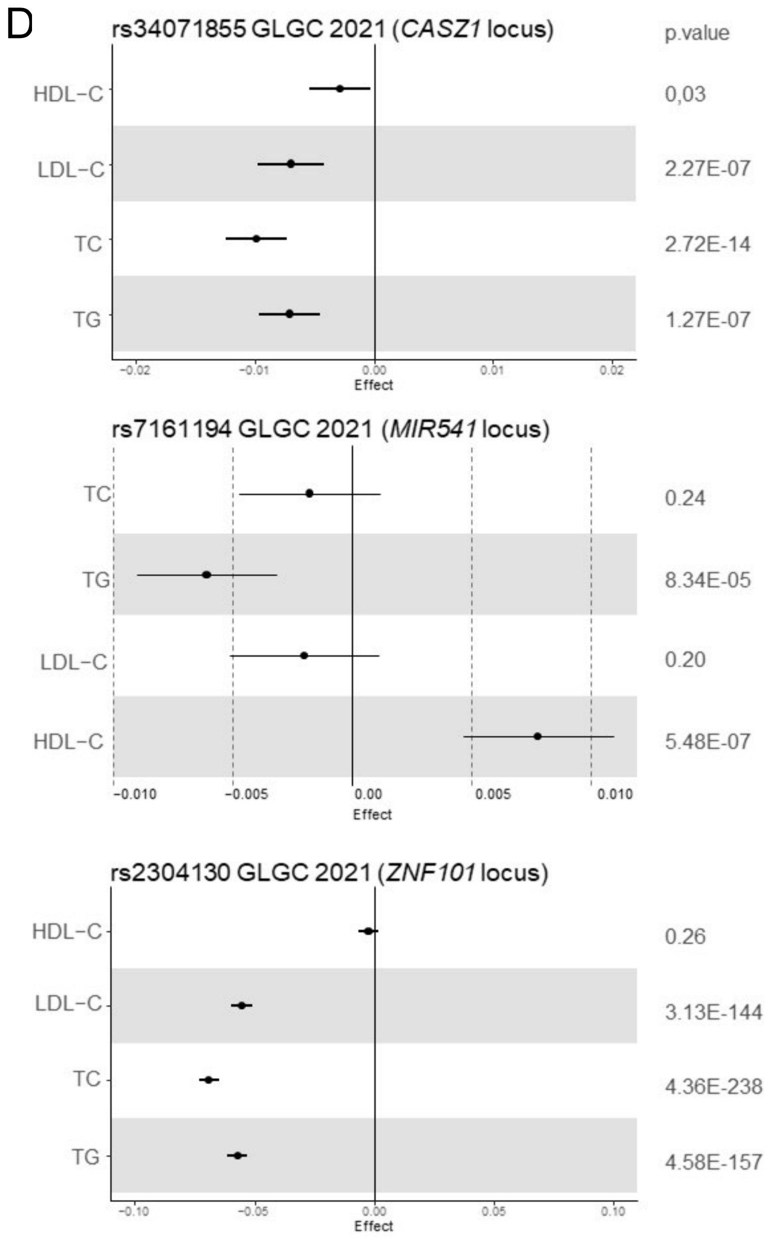

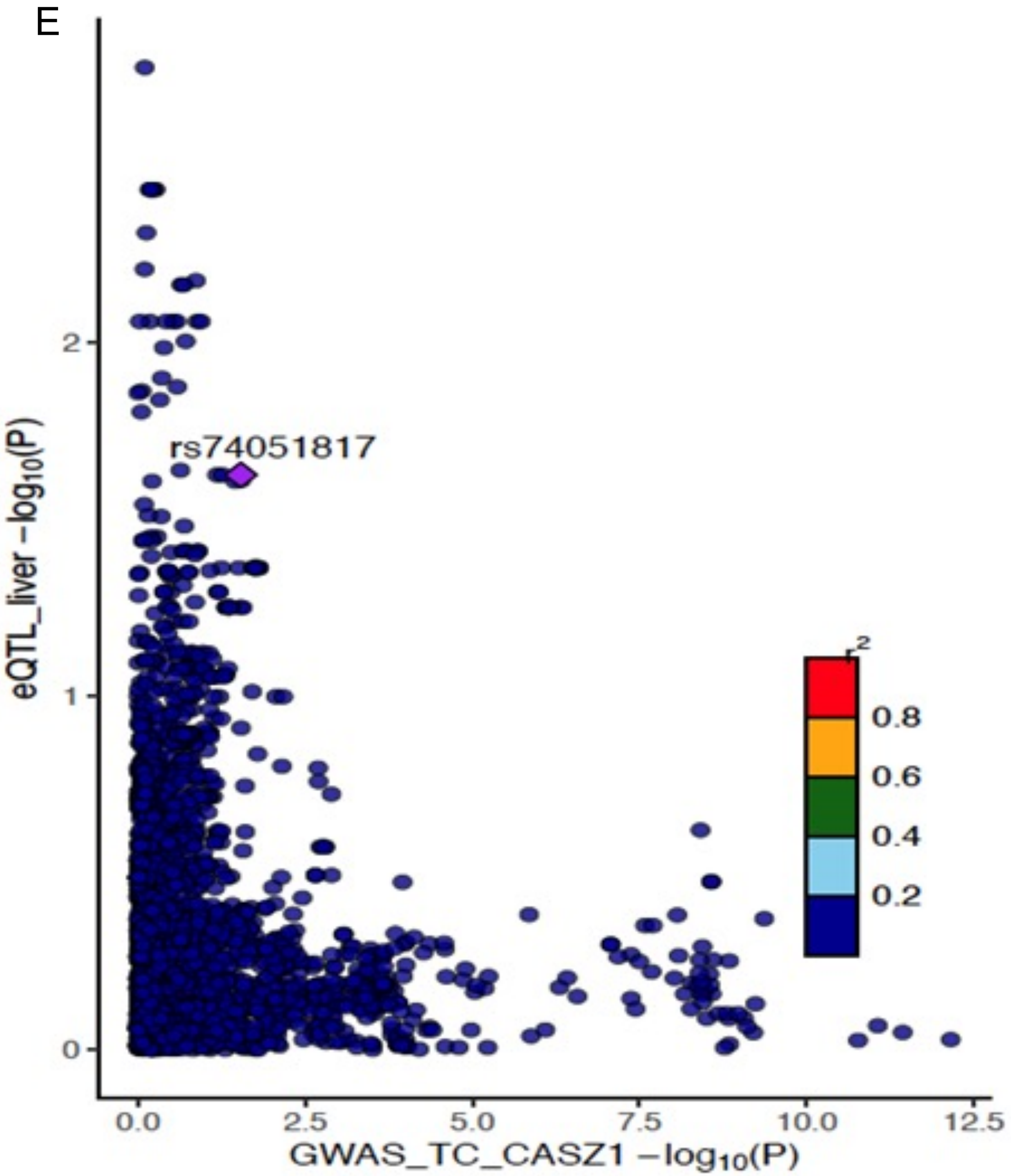

Supplementary Fig 5. Genome wide associations of variants in the *MIR541*, *CASZ1,* and *ZNF101* loci with plasma lipids and lipoproteins.

(A-C) Association results for SNPs (-log10 P value, y-axis) as a function of genomic coordinates using human gene version 19 (hg19) for (A) *MIR541*, (B) *CASZ1,* and (C) *ZNF101* gene loci with plasma total cholesterol, LDL, apoB, HDL-C, apoA1, and triglycerides in the UK Biobank using the Pan-UKB project summary statistics. The bottom panel shows genes at each locus, as annotated in the UCSC Genome Browser. The most highly associated SNPs are represented as purple diamonds, and the linkage disequilibrium (LD) values (1000 Genomes data) are in the inset. Light blue dotted lines indicate estimated recombination hotspots. Forrest plots (right panels) show the associations (beta ± standard error) of the top associated SNP with plasma lipids and apolipoproteins levels. (*) Correspond to genome wide significant p-values ($p<5.0E-08$). Raw data are presented in Supplemental Table 3.

(D-E) (D) Forrest plots show the associations (beta ± standard error) of the top associated SNP with plasma lipids and apolipoproteins levels in GLGC. (E) LocusCompare plot shows the relationship between SNPs in the *CASZ1* locus (±100kb) and their association with total cholesterol plasma levels in the x-axis with the gene expression of *CASZ1* in the liver (eQTL_liver-log10(P) from GTEx dataset V7) in the y-axis.

Supplementary Fig 6

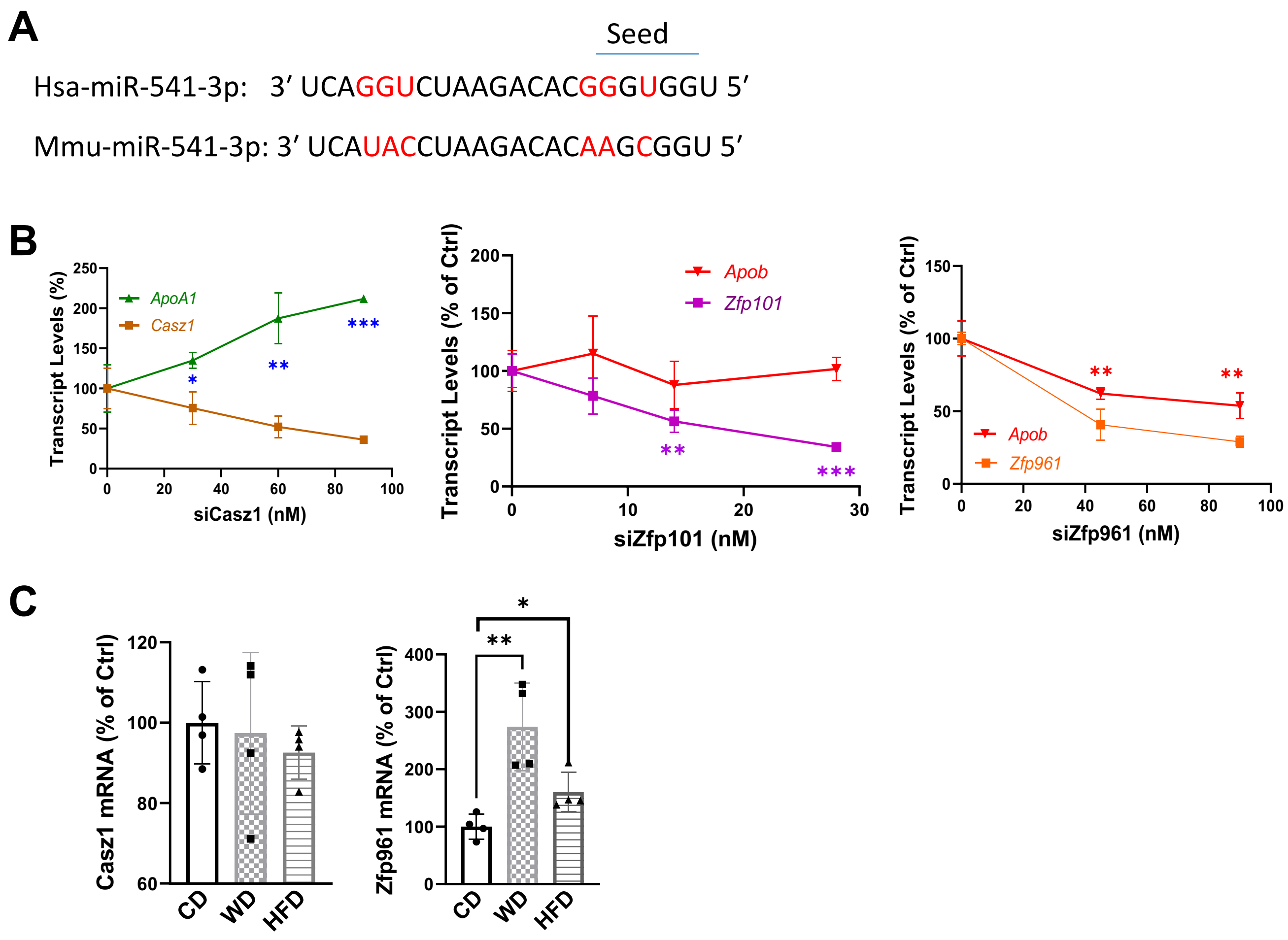

Supplementary Fig 5. Mouse orthologs of human genes.

(**A**) Human and mouse miR-541-3p sequences are compared to highlight sequence differences (red). Hsa-miR-541-3p and mmu-miR-541-3p are not identical. They contain three different bases in seed and non-seed sequences each.

(**B**) Identification of mouse orthologs of human Znf101 and Casz1 and their role in the regulation of mouse apoB and apoA1 expression. Mouse liver AML12 cells were transfected in triplicate with different concentrations of mouse siCasz1 (left), siZfp101 (middle) or siZfp961 (right). After 48 h, the indicated mouse mRNAs were quantified in triplicate. These studies identified *Casz1* and *Zfp961* as functional mouse orthologs of human *CASZ1* and *ZNF101* genes. * $P < 0.05$; ** $P < 0.01$; ***$P < 0.001$; ****$P < 0.0001$.

(**C**) Male C57Bl6 mice (*n* = 4, 5 months old) were fed chow (CD), Western (WD) or obesogenic (HFD) diets for 13 weeks. Livers were collected to measure mRNA levels. Zfp961 expression increased in the livers of high fat diet fed mice. One-way ANOVA, * $P < 0.05$, **$P < 0.001$.

Supplementary Fig 7

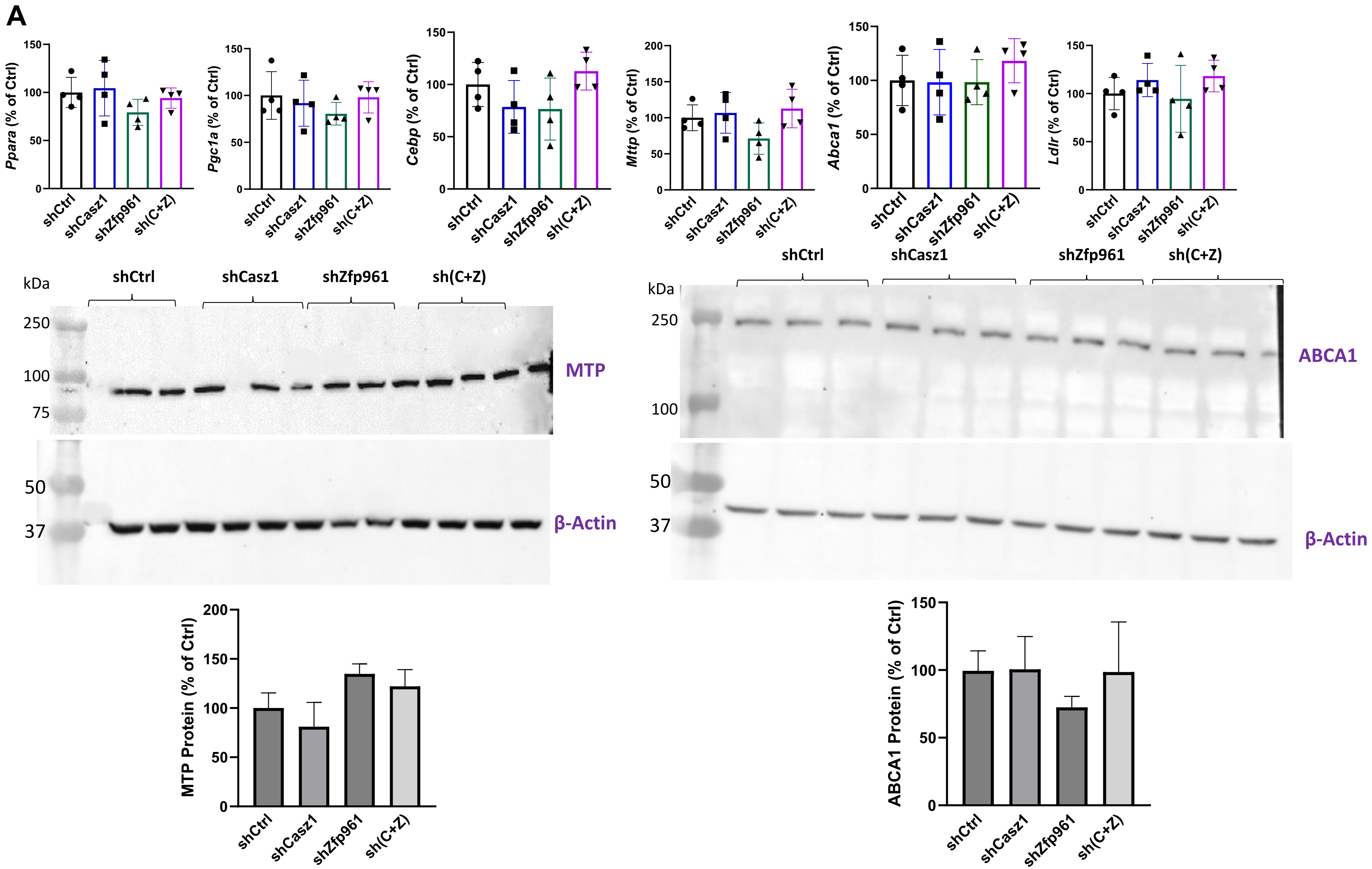

Supplementary Fig 7

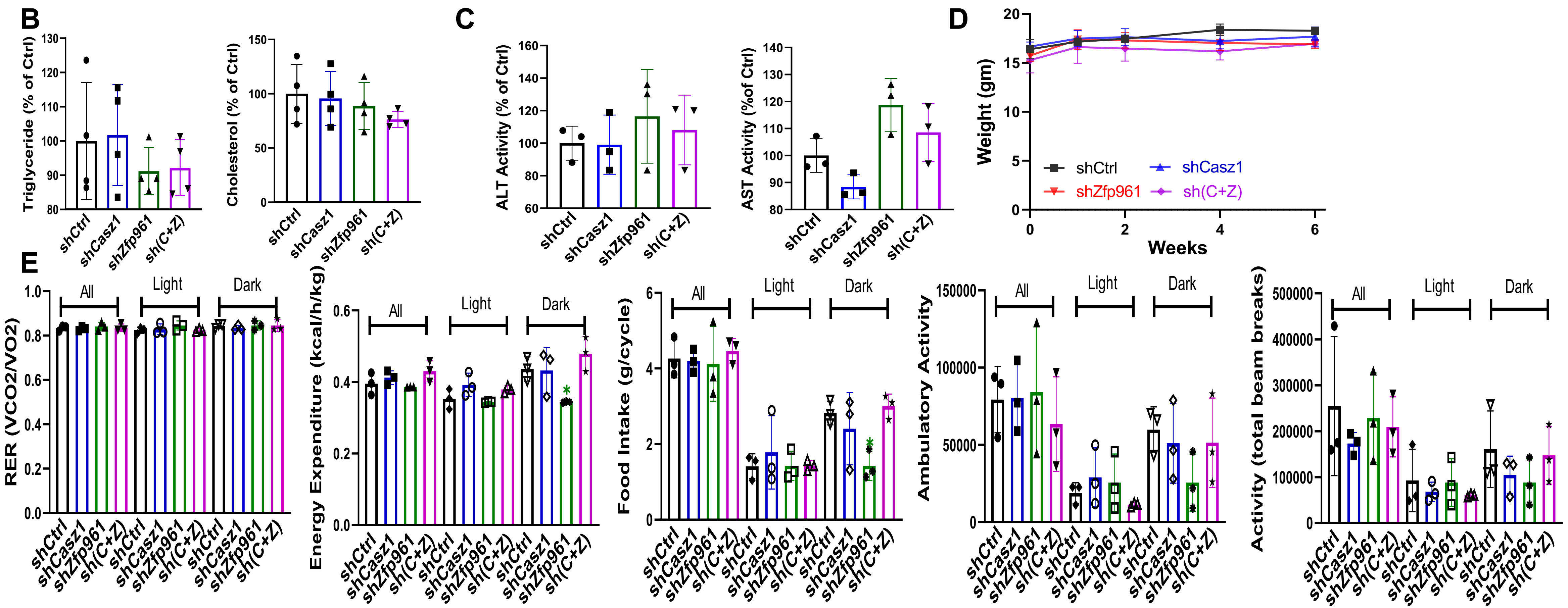

Supplementary Fig 7. Effect of hepatic Casz1 and Zfp961 knockdown on different parameters in mice.

Mice (C57Bl6J, female, 2.5-month-old) were transduced with AAV8 (2.5 x 10^{11} gc/mouse) expressing shControl (shCtrl, *n* = 4), shCasz1 (*n* = 4) or shZfp961 (*n* = 4), alone or combined (sh(C+Z), *n* = 5), and started on a Western diet.

(**A**) After 6 weeks, livers were collected to measure in triplicate different TFs and lipid metabolism genes. KD of different genes had no effect on their expression (top). Western blot analysis was performed to measure changes in MTP and ABCA1 (bottom). No significant differences in the expression of MTP and ABCA1 were found amongst different groups.

(**B**) After 6 weeks, lipids were extracted from liver slices and triglyceride and cholesterol levels were measured in triplicate and normalized with protein levels. KD of different genes had no effect on hepatic lipids.

(**C**) Plasma was used to measure ALT and AST activities in triplicate. These enzyme activities were unaffected by KD of Casz1 or Zfp961.

(**D**) Weight gain in mice was monitored over the course of 6 weeks. No significant differences were observed.

(**E**) After 5 week of transductions, mice were placed in CLAMS to measure different physiological parameters. No significant differences were noted.

Supplementary Fig 8

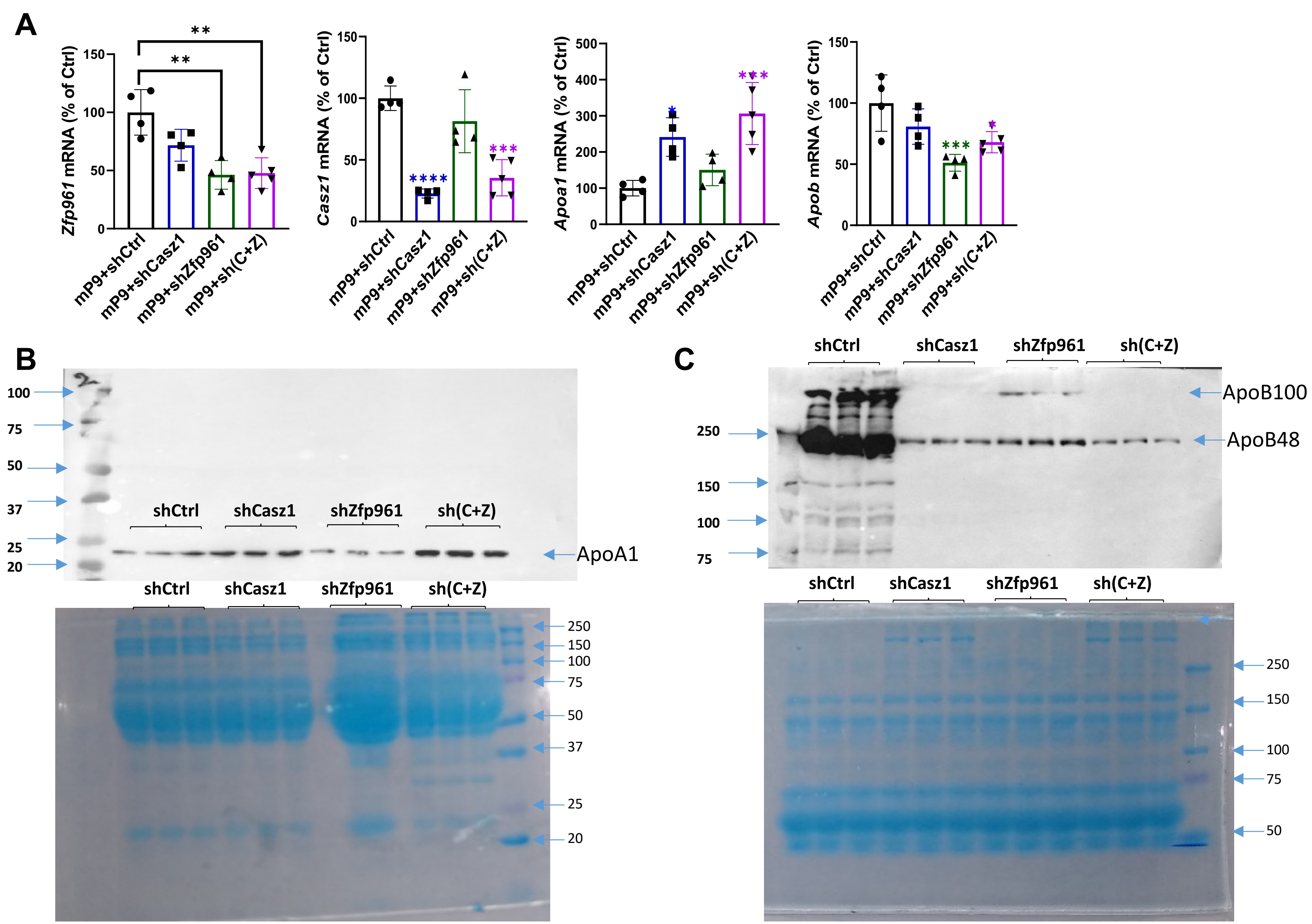

Supplementary Fig 8. Effect of knockdown of different TFs on atherosclerosis.

All mice received AA8 expressing mutant mouse PCSK9. In addition, mice were transduced with shCtrl, shCasz1, shZnf101, or shCasz1+shZnf101, and started on a Western diet.

(**A**) After 4 months, livers were collected to measure mRNA levels in triplicates.

(**B**) Detection of plasma apoA1 (top) by western blotting. Plasma (1 µL) was separated on a 10% gel, transferred, and probed with anti-apoA1 antibodies. Plasma (1 µL) was separated and stained with Coomassie blue (bottom) for control.

(**C**) Detection of apoB by western blotting (top) after separating plasma (1 µL) on a 6% gel. Total plasma was separated and stained with Coomassie blue (bottom) for control.

Supplementary Fig 9

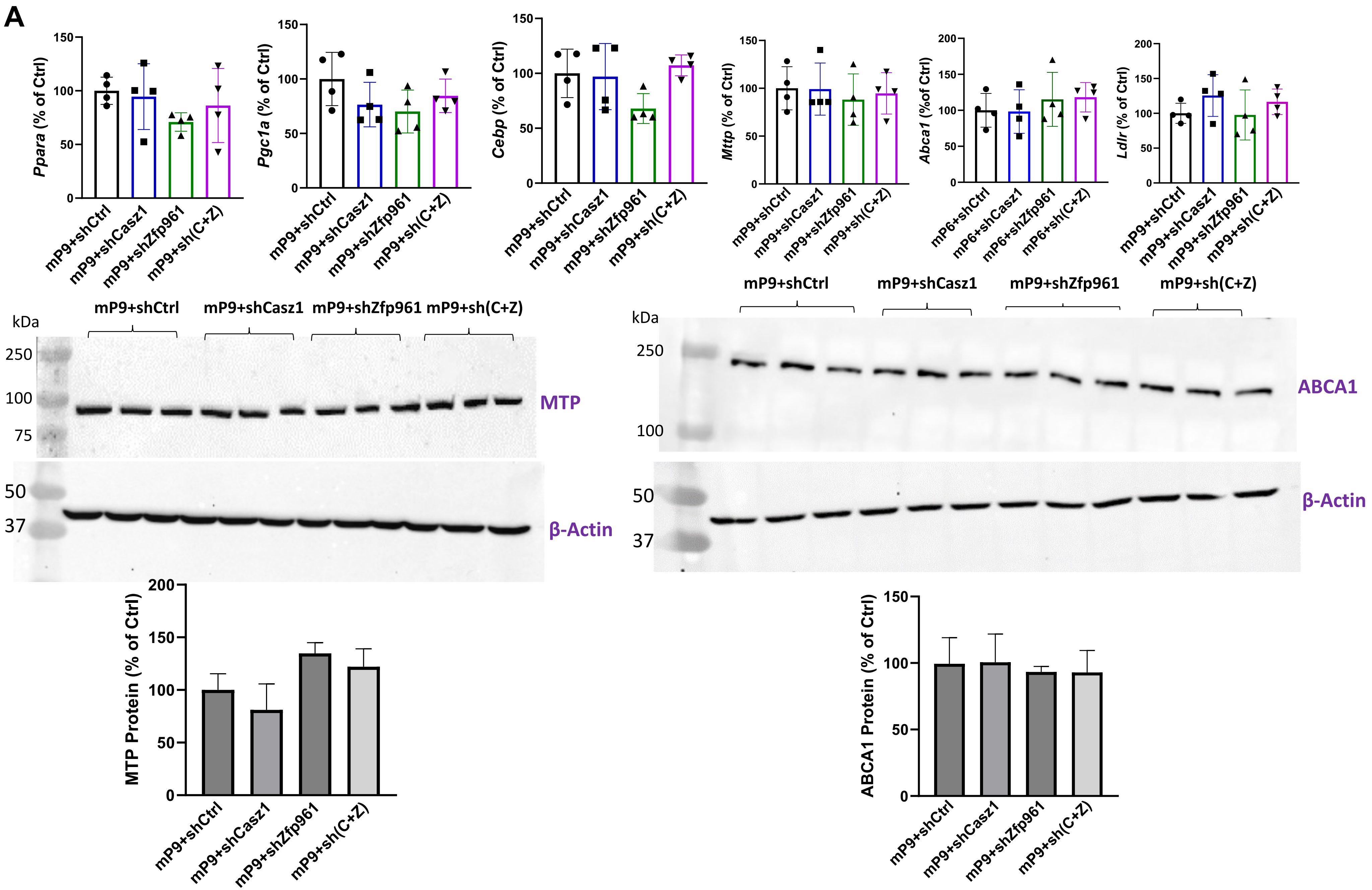

Supplementary Fig 9

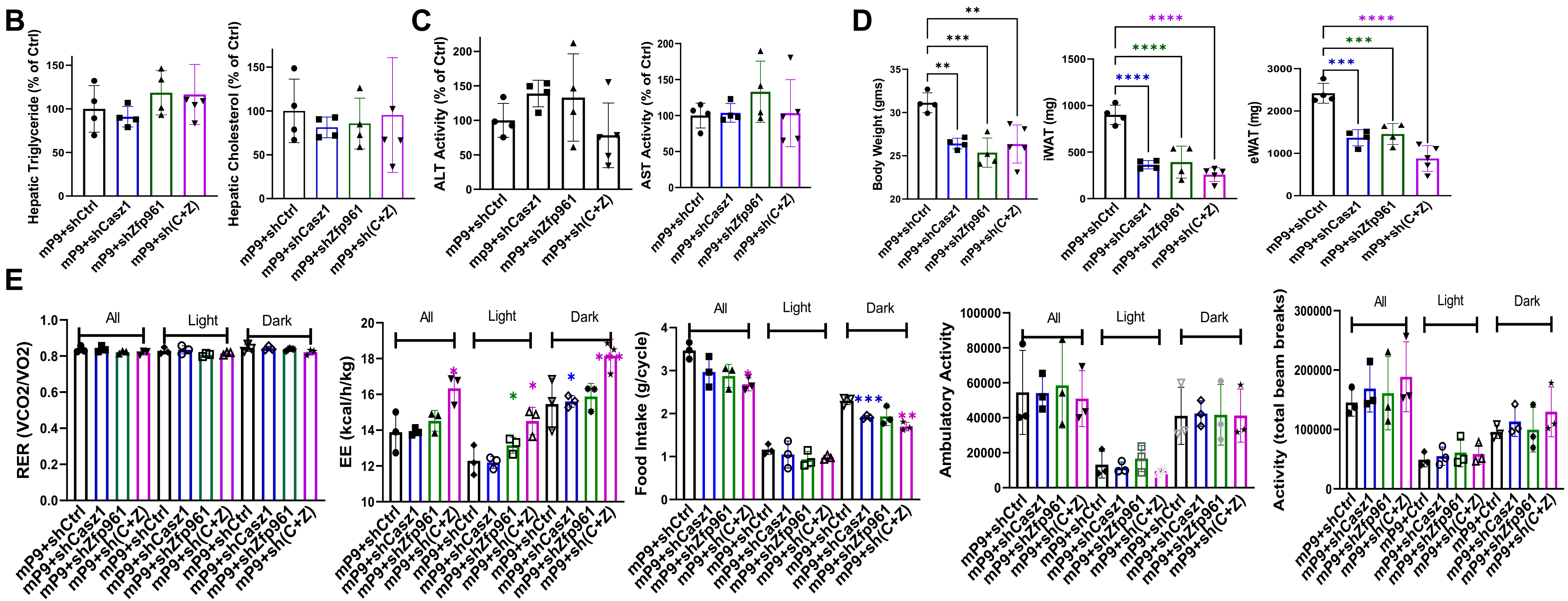

Supplementary Fig 9. Effect of knockdown of different TFs on physiological parameters. Mice were transduced with a combination of adenoviruses expressing gain-of-function mutant Pcsk9 and those expressing different shRNAs as in Fig 5.

(**A**) After 4 months, livers were collected to measure TFs and lipid metabolism genes in triplicates (top). Western blot analyses were performed to measure changes in MTP and ABCA1 proteins levels (bottom). MTP and ABCA1 mRNA and protein levels were similar in all the groups indicating that KD of different TFs had no effect on their expression.

(**B**) Lipids were measured in triplicates in livers and normalized to protein levels. Knockdown of different TFs had no effect on hepatic lipids.

(**C**) Plasma was used to measure AST/ALT activities in triplicates.

(**D**) At the end, different tissues were collected. Total body and adipose tissue weights in different knockdown mice were lower than in control mice (right). No differences in other organ weights were observed.

(**E**) After 3 months, mice were placed in CLAMS (comprehensive laboratory animal monitoring system) to monitor physiological indices.

Supplementary Fig 10

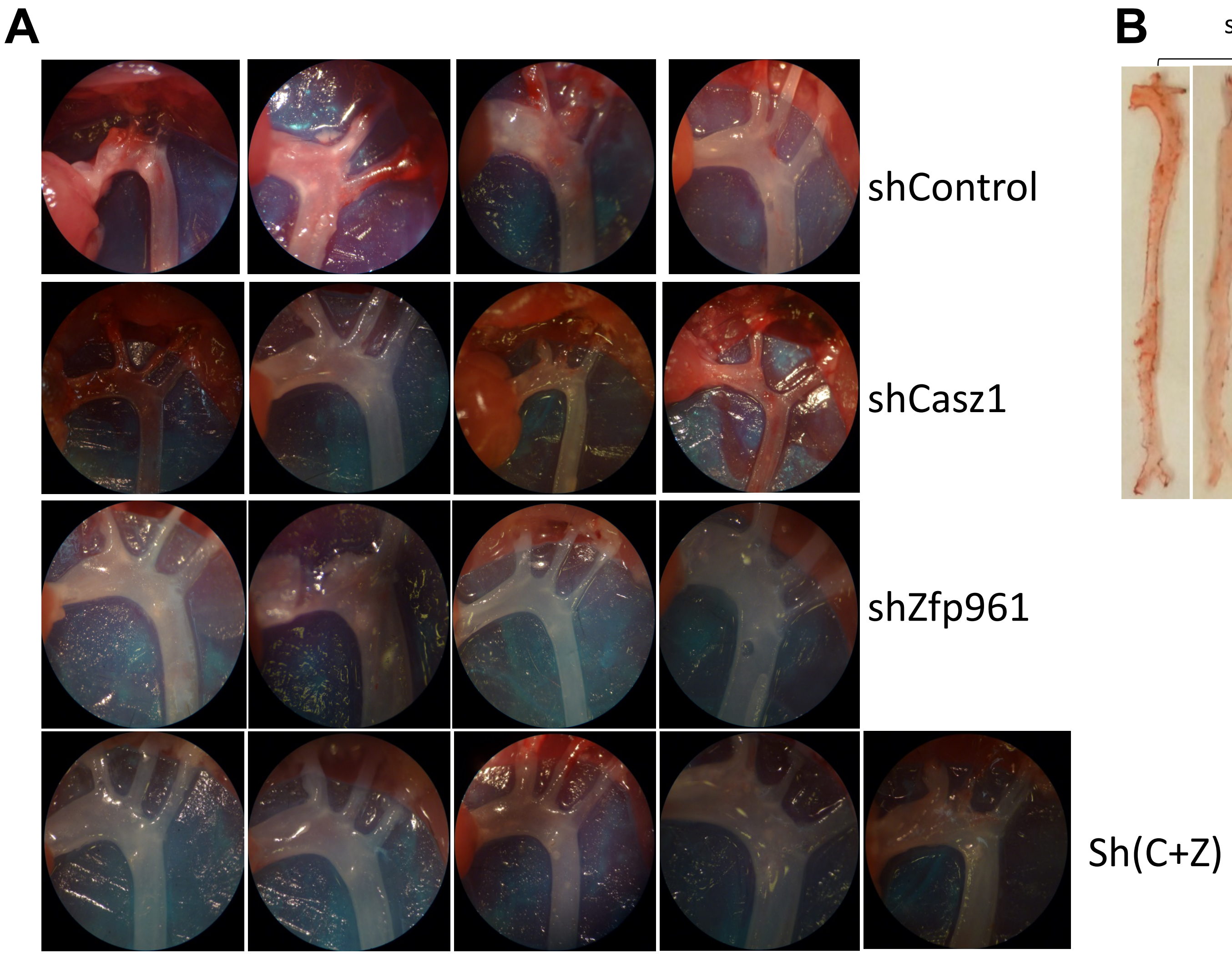

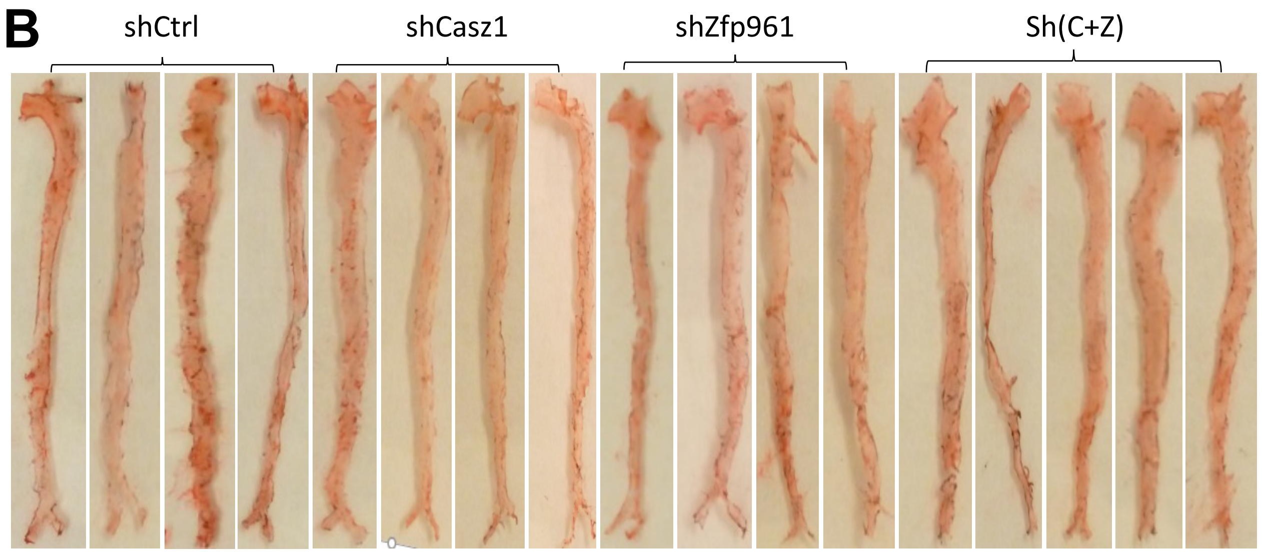

Supplementary Fig 10. Effect of knockdown of different TFs on atherosclerosis.

Mice were transduced with adenoviruses expressing gain-of-function mutant Pcsk9 and those expressing different shRNAs as in Fig 5.

(**A**) Aortas with major arteries were dissected from all mice, photographed, and presented as a collage.

(**B**) Whole aortas were stained with Oil Red O and all images were compiled.

Supplementary Fig 11

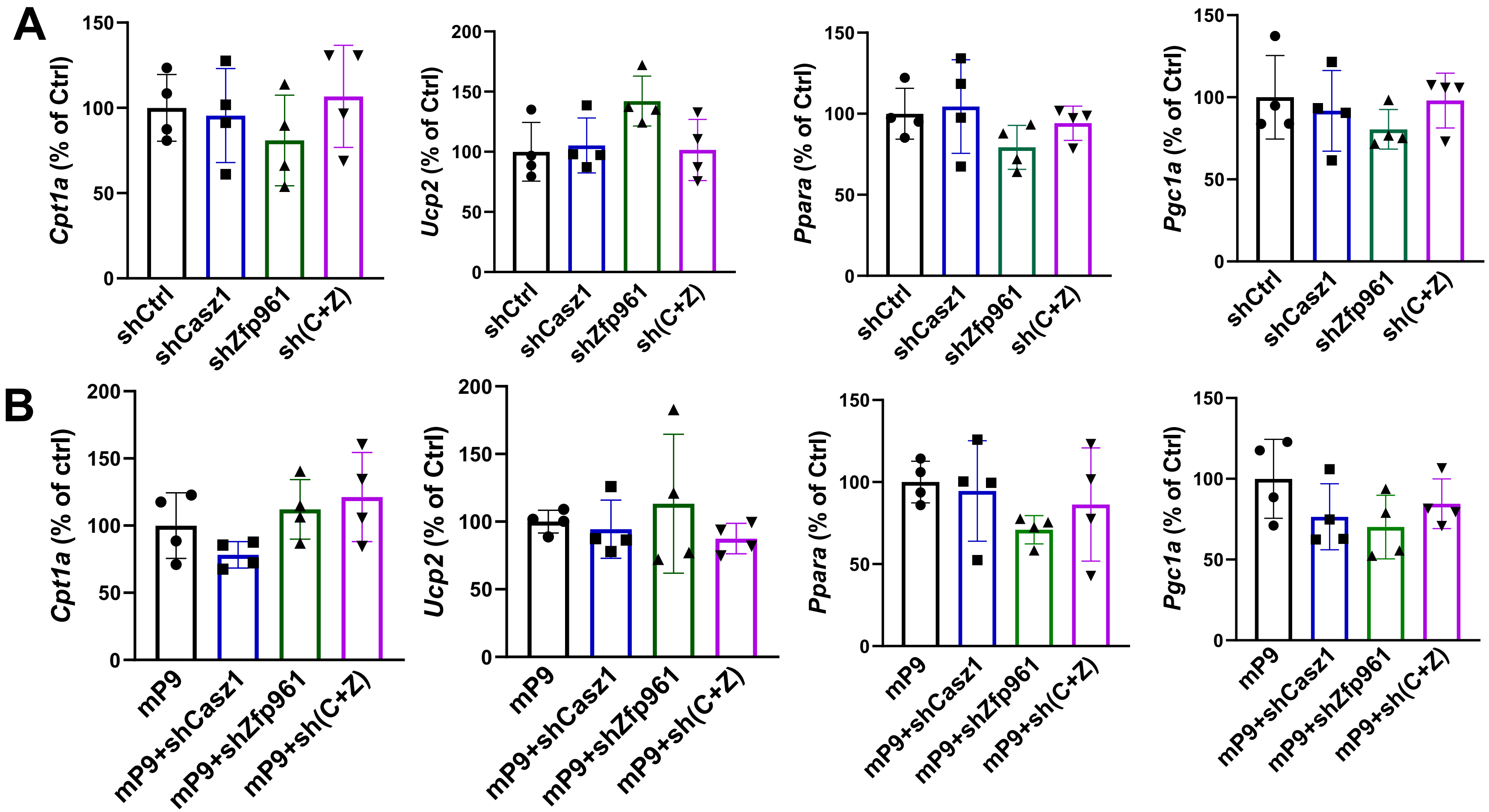

Supplementary Fig 11

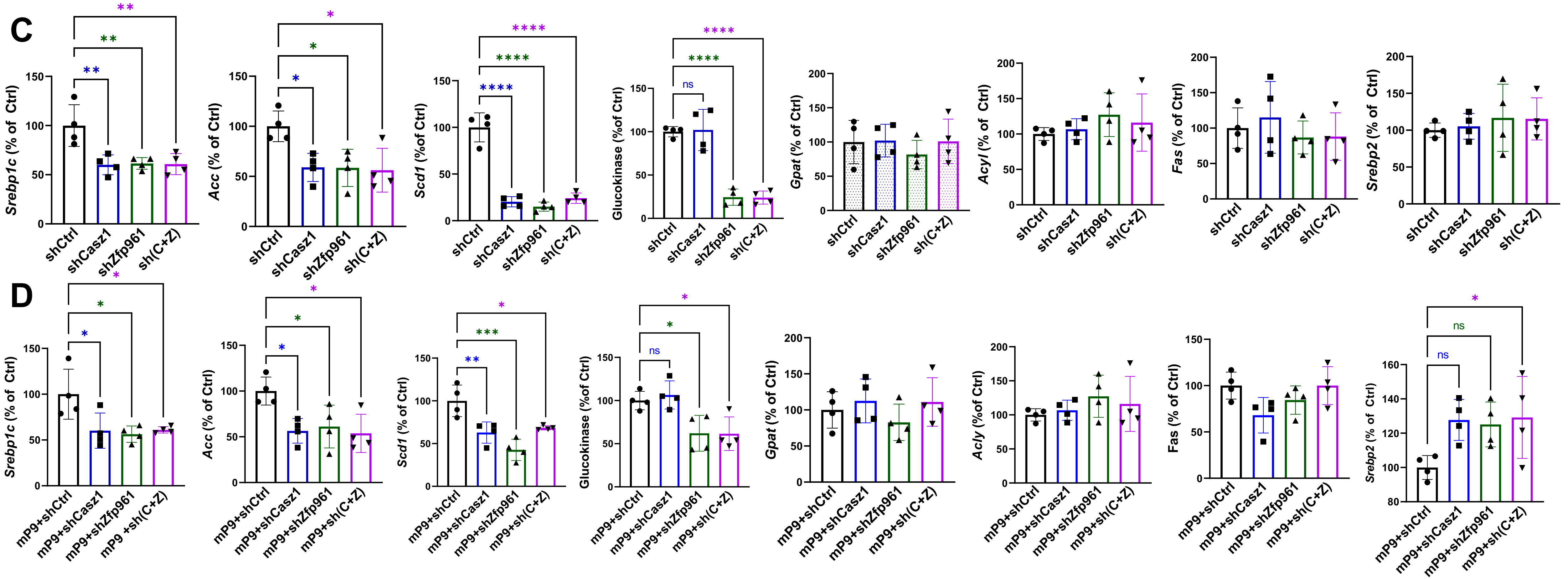

Supplementary Fig 11. Hepatic knockdown of Casz1 and Znf961 has no effect on the mRNA levels of genes involved in β-oxidation, but reduces the expression of genes in lipogenesis.

Mice were transduced with viruses as in Figs 4 and 5. Livers from these mice were used to quantify in triplicate different mRNAs involved in β-oxidation (A, B) and lipogenesis (C, D).

Supplementary Table 2. Physical parameters in different mice transduced with different shRNAs measured using dual-energy x-ray absorptiometry (DEXA). Mice were transduced with different shRNAs as described in Fig 4. DEXA was performed 3 months after the injection of viruses. All KD mice showed significantly less fat weight. Data are average +SD, Student *t*-test compared to controls.

DEXA parameters	shCtrl (n=4)	shCasz1 (n=4)	shZfp961 (n=4)	sh(C+Z) (n=5)
Total Weight (gms)	23.11 ± 0.55	24.60 ± 1.32<br>P = 1.72	23.59 ± 0.18<br>P = 0.86	21.31 ± 1.50,<br>P = 0.06
Soft Weight (gms)	21.91 ± 0.49	23.61 ± 1.42<br>P = 0.13	22.63 ± 0.13<br>P = 0.70	20.34 ± 1.60<br>P = 0.14
Lean Weight (gms)	12.82 ± 2.92	16.20 ± 2.92<br>P = 0.19	16.38 ± 0.95<br>P = 0.17	13.99 ± 2.75<br>P = 0.88
Fat Weight (gms)	10.59 ± 0.75	7.41 ± 1.69<br>**P = 0.001**	6.25 ± 0.86<br>**P = 0.0002**	6.35 ±1.75<br>**p<0.0001**
Fat (% of body weight)	46.46 ± 4.05	31.72 ± 8.83<br>**P = 0.003**	27.64 ± 3.92<br>**P = 0.0005**	31.56 ± 0.94<br>**P = 0.0003**
Bone Mineral Content (gms)	1.25 ± 0.17	0.98 ± 0.11<br>P = 0.08	0.96 ± 0.11<br>P = 0.052	0.97 ± 0.18<br>**P = 0.05**
Bone Mineral Density (mg/cm^2)	88.66 ± 3.82	87.76 ± 3.72<br>P = 0.99	90.52 ± 7.65<br>P = 0.94	88.42 ± 6.85<br>P = 0.99

Supplementary Table 3. Total weight gain was less in different mice transduced with shRNAs to KD Casz1 and Zfp961. Mice were transduced with different shRNAs as described in Fig 5. DEXA was performed 3 months after the injection of viruses. All KD mice showed significantly less total; soft, lean and fat weights. Data are average +SD, Student *t*-test compared to controls.

DEXA Parameters	mPcsk9+shCtrl (n=4)	mPcsk9+shCasz1 (n=4)	mPcsk9+shZfp961 (n=4)	mPcsk9+sh(C+Z) (n=5)
Total Weight (gms)	40.97 ± 0.82	34.18 ± 2.58 **P = 0.024**	36.17 ± 1.47 **P = 0.001**	30.58 ± 3.16 **P = 0.0004**
Soft Weight (gms)	40.00 ± 0.81	33.28 ± 2.59 **P = 0.002**	35.21 ± 1.38 **P = 0.001**	29.61 ± 3.17 **P = 0.002**
Lean Weight (gms)	27.05 ± 0.49	22.83 ± 1.35 **P = 0.001**	23.47 ± 2.61 **P = 0.041**	20.07 ± 2.54 **P = 0.0004**
Fat Weight (gms)	13.17 ± 0.58	10.45 ± 1.37 **P = 0.013**	10.24 ± 1.09 **P = 0.005**	9.54 ± 1.01 **P = 0.0006**
Fat (% of body weight)	32.93 ± 1.12	31.31 ± 1.92 P = 0.19	33.25 ± 8.01 P = 0.94	32.27 ± 2.46 P = 0.64
Bone Mineral Content (gms)	0.98 ± 0.01	0.90 ± 0.02 **P = 0.003**	0.96 ± 0.10 P = 0.82	0.96 ± 0.08 P = 0.76
Bone Mineral Density (mg/cm^2)	86.32 ± 1.50	85.38 ± 2.26 P = 0.51	86.12 ± 0.77 P = 0.81	89.55 ± 2.30 P = 0.04

Data are average +SD. Student t-test

SUPPLEMENTARY TABLE 4: Different diets, chemicals and siRNAs used and their sources have been tabulated. In addition, this table contains primer sequences used in the study for mRNA quantitation and site-directed mutagenesis.

Diets	% Fat	Company, ID
Chow	13%	Lab Diet, 5053
Western	45%	Envigo, TD.88137
Obesogenic	60%	Research Diets, D12492

siRNAs oligo	
Universal scrambled negative control	CGUUAAUCGCGUAUAAUACGCGUAT
ZNF101 Human siRNA Oligo Duplex	GAAGUCUAAGGUAAUUUGAAAAAGUAA
CASZ1 Human siRNA Oligo Duplex	UUUGGAGAAGGUUGACUUCAGGUACUC
Casz1 Mouse siRNA Oligo Duplex	CGUGAAGGAUCCCAGUAAUGAAUCA
Zfp961 Mouse siRNA Oligo Duplex	ACACUGCCAGUCAAUCAUCCCUGAA

Reagents		
Items	Vendor	Catalog #
Hsa-miR-541-3p	Thermo Fisher Scientific	4464066, Assay ID#MC13119
Hsa-AntimiR-541-3p	Thermo Fisher Scientific	4464084, Assay ID# MH13119
Hsa-miR-541-5p	Thermo Fisher Scientific	4464066, Assay ID#MC12516
miRNA Negative Control	Thermo Fisher Scientific	4464058
TaqMan™ miR-541-3p Assay	Thermo Fisher Scientific	4427975, Assay ID#002201
TaqMan™ miR-U6 Assay	Thermo Fisher Scientific	4427975, Assay ID#001973
TaqMan™ MicroRNA Reverse Transcription Kit	Thermo Fisher Scientific	4366596
TaqMan™ Universal Master Mix II, no UNG	Thermo Fisher Scientific	4440043
PowerTrack SYBR Green Master Mix	Thermo Fisher Scientific	A46012
High-Capacity cDNA Reverse Transcription Kit	Thermo Fisher Scientific	4374967
TRIzol™ Reagent	Thermo Fisher Scientific	15596026
ELISA Pro: Human apoB	MABTECH	3715-1HP-20
Human Apolipoprotein A-I/ApoA1 DuoSet ELISA	R&D system	DY3664-05
miRNA Target clone control vector	Genecopoeia	CmiT000001-MT05

miRNA 3´UTR target expression clone for Human ZNF101	Genecopoeia	HmiT071405-MT05
miRNA 3´UTR target expression clone for Human CASZ1	Genecopoeia	HmiT105330-MT05
Custom promoter reporter clone for Human APOB	Genecopoeia	CS-HPRM30361-PG04-02
Promoter reporter clone for Human APOA1	Genecopoeia	HPRM25646-PG04
Zfp961-AAVPrime™ Purified AAV Particles	Genecopoeia	AA08-MSH074798-AV03
Casz1-AAVPrime™ Purified AAV Particles	Genecopoeia	AA08-MSH034915-AV03
AAVPrime™ Purified AAV Particles for shRNA scrambled control	Genecopoeia	AA08-CSHCTR001-AV03
AAV8-D377y-mPCSK9 (5e12 gc)	Vector biosystems Inc	7600
Secrete-Pair Dual Luminescence Assay		LF031
Apolipoprotein A-I/ApoA1 Antibody	NOVUS	NBP2-15429
Apolipoprotein B (APOB), Polyclonal Antibody	MyBiosource	MBS2006107
Anti-Argonaute-2 antibody	Abcam	ab186733
Anti-Casz1 antibody	Rockland	600-401-B62
Anti-Znf101 antibody	Thermo Fisher	25599-1-AP
Pointe Scientific Triglycerides Liquid Reagent	Pointe Scientific	23-666-411
Pointe Scientific Cholesterol Liquid Reagents	Pointe Scientific	23-666-200
EndoFectin™ Max transfection Reagent	Genecopoeia	EF013
Protease Inhibitor Cocktail	Sigma Aldrich	P1860
RIPA buffer	Thermo Fisher Scientific	J63306
Poloxamer 40716758	Sigma Aldrich	40716758
Olive oil	Sigma Aldrich	01514
T0901317, LXR agonist	Abcam	ab142808
ScintiSafe™ Econo 2 Cocktail	Thermo Fisher Scientific	FISSX21-5
Cholesterol, [1,2-3H(N)]	Perkin Elmer	NET139001MC

Human primers		
Gene name	Forward primer	Reverse primer
ZNF101	GGAAGCCGGAAATGGACTCAG	CATTGGATTCCGACCGAGGC
MAFK	GCACACATGGCAGAGAGAGT	GAGTCCTGCTCACCGTCAAA
NFATC3	GCTCTCATGTCCAGCTCCTTT	AGTTGGTGGTGGACACAAGG
RELA	TCCAGTGTGTGAAGAAGCGG	TCCCCACGCTGCTCTTCTAT
ZNF275	AAGTCCTTCCGAGGGGTCAA	GAAGGCCGCAGGCGTAG
ELK4	TCACGAGCAGTGATCCAAGC	AAGAGCGAGCAAGCTACCTG
HSF1	GAAGCAGCTGGTGCACTACA	CTGTCAGCAGGGAGATGGTG
ZNF442	ACCTCATGTGTTGTAAAAGCTATGT	CTATGTAGGCAGGCCTGTGA
ZSCAN22	GCGCAAGTTGGCTAGTCTCT	GGAAGCTGTCCTCTTCCCAC
ZNF746	GGACCCTGAAGCTCAACACA	CTTTCCCAGGCTCCTTCCTG
CASZ1	CCGAGGGTGTCTACATGGTG	CCCGTCCGAATCCTTCTCC

HNF4A	GCAATGACACGTCCCCATCA	TCGAGGCACCGTAGTGTTTG
ZNF771	AGGGGGTGGGGCTATATGTTC	CAGGCATCTTGGTGTCCTGA G
IRF3	ACACATACTGGGCAGTGAGC	CTACAATGAAGGGCCCCAGG
THRB	TGCGCTTGGAAAAGAGACCT	AATTACCAGTGCCCTGGAGC
NR4A3	TGCGTCCAAGCCCAATATAGC	GGTGTATTCCGAGCTGTATGT CT
ZBTB4	TCCCTTTTGCACTGAGGCTT	CCTTCGATTCGCAGAGCAGA
ZNf100	AGGGGCCATTGACGTTTAGG	AGGGCTCTTTTCCTTGCTCC
RARA	CACACACCTGAGCAGCATCAC	TCAAGAGCCGGTCCTTTGGT
ZSCAN16	TAAGGCAGAGGACCATTACTGG	TGCATCCTGATAGCAGAGCTT
ZNF726	ATGAACCCCCAGGTAGGTGA	TTCAGGCTTTCCAGAAACGGT
YY1	ACGGCTTCGAGGATCAGATTC	TGACCAGCGTTTGTTCAATGT
ZNF4471	GTCCTTCGGATGAGAGCGTC	AGCAGGGTTCATCCATTGCC
ZNF81	GTTCTCAGCCGGGGTTTGAT	CTGCTGGGGTCAGAAGGAAG
SP6	CTTCCTTGCTGAGAGGGTTG	TAGGATGGCCACTGGAAAAG
FOXI1 (NM_012188.5)	GGAAGTCACCAGTGGCCTTA	GGTGGGAGGATTTAGGGGTA
ONECUT3	AAGGAACCTCCTCCCTGAAA	GCACAAATCCTTGGGAGAAA
ZNF418	GGGTGTGTTTCTGCGGTTTG	CCTCTGTGCAGCAAGAAGGA
MTP		
ABCA1	AACAGTTTGTGGCCCTTTTG	AGTTCCAGGCTGGGGTACTT
ApoA1	AGAGACTGCGAGAAGGAGGT	TCTCTGCCGCTGTCTTTGAG
ApoB	TGTCAGTACACACTGGACGC	TCAAATGCGAGGCCCATCTT
SDM_apoa1 luciferase promoter plasmid	GCGTGATCAAcgcTAAGTGTGAA C AATGCAAAGG	ACAGGTGTGACTGGATCTC
SDM_ApoB luciferase promoter plasmid with mutated bases	AAGGCATCTGataATGGGGCGTG	ACTTGGACAGACCAGGCT
**Mice primers**		
Casz1	CCTCCAACTGCCTGAGCTAC	CGGGGTCCTAGAGATTCCTC
Zfp961	TGACCCATGTGGAGAAACGG	ACAGTGCCGGTAACTGAAGG
Zfp101	GGTGAATGTGAACGTGGTTG	CCCAGCATTCCTCAGGTTTA
Apoa1	GGCCGTGGCTCTGGTCTT	AGCGTGGTGAAAGGGCTTAT
ApoB	TCCATATTCCAGACAACCTCTTC	GTTTATTTTGTTCCTGTTCATT GTGT
HNF4α	ACAGGAGAGGGTCAGAAGCA	ATGTTTGCACAACCACAGGA
PPARα	CACGCATGTGAAGGCTGT	GCTCCGATCACACTTGTCG
PGCa1α	CTGTCGAGTCTGTTGGAGCA	GGGAATGTCAATGCCTGAGT
C/EBP	TTACAACAGGCCAGGTTTCC	CTCTGGGATGGATCGATTGT
MTP	CACACAACTGGCTCTCTCATTAA AT	TGCCCCCATCAAGAAACACT
ABCA1	AACAGTTTGTGGCCCTTTTG	AGTTCCAGGCTGGGGTACTT
LDLR	GAAAAGGCTACTGGCTGTGC	CCAGGACCCGGTCAGTAGTA
SREBP2	CCATCTTCCCCTCTCTTTCC	AGGGAAGATCCTGGGAGAAA
SCD1	CTTCAAGGGCAGTTCTGAGG	CAATGGTTTTCATGGCAGTG
Glucokinase	CTTTCCAGGCCACAAACATT	TGAGTGTTGAAGCTGCCATC

mGpat	AGCAAGTCCTGCGCTATCAT	CTCGTGTGGGTGATTGTGAC
ACLY	AGGTCTCTCTGCAGCCATGT	AAGCTTTCCTCGACGTTTGA
ACC	GCCTCTTCCTGACAAACGAG	TGACTGCCGAAACATCTCTG
SREBP1c	AGGTGTATTTGCTGGCTTGGT	AGAGATGACTAGGGAACTGT GTGT
FAS	CTCAGTGTGCCCACCTA G	GCACTTGCTTGATGCAATCT
UCP1	CGTGAAGGTCAGAATGCAA	GCATTGTAGGTCCCCGTG
UCP2	GCGTTCTGGGTACCATCCTA	GCTCTGAGCCCTTGGTGTAG
CPT1	GACTCCGCTCGCTCATTC	TCTGCCATCTTGAGTGGTGA
HNF4α	ACAGGAGAGGGTCAGAAGCA	ATGTTTGCACAACCACAGGA
PPARα	CACGCATGTGAAGGCTGT	GCTCCGATCACACTTGTCG
PGCa1α	CTGTCGAGTCTGTTGGAGCA	GGGAATGTCAATGCCTGAGT
C/EBP	TTACAACAGGCCAGGTTTCC	CTCTGGGATGGATCGATTGT

Supplementary Table I. The effect of 1237 miRs on apoB and apoAI secretion in Huh7 cells.
Medium apoB and apoAI were normalized against protein levels and calculated to percentages of Scr control.

			apoB		apoAI	
Mature Name	Mature Accession	Mature Sequence	plate1	plate2	plate1	plate2
hsa-let-7a-2-3p	MIMAT0010195	CUGUACAGCCUCCUAGCUUUCC	110.79	94.94	114.44	102.87
hsa-let-7a-3p	MIMAT0004481	CUAUACAAUCUACUGUCUUUC	112.31	86.33	73.30	59.51
hsa-let-7a-5p	MIMAT0000062	UGAGGUAGUAGGUUGUAUAGUU	35.73	56.36	72.34	83.45
hsa-let-7b-3p	MIMAT0004482	CUAUACAACCUACUGCCUUCCC	105.54	109.86	90.36	91.48
hsa-let-7b-5p	MIMAT0000063	UGAGGUAGUAGGUUGUGUGGUU	77.29	57.43	107.87	115.59
hsa-let-7c	MIMAT0000064	UGAGGUAGUAGGUUGUAUGGUU	67.69	62.76	78.38	70.12
hsa-let-7c*	MIMAT0004483	UAGAGUUACACCCUGGGAGUUA	89.15	94.94	114.44	107.62
hsa-let-7d-3p	MIMAT0004484	CUAUACGACCUGCUGCCUUUCU	158.43	246.59	91.70	95.20
hsa-let-7d-5p	MIMAT0000065	AGAGGUAGUAGGUUGCAUAGUU	32.34	31.45	62.74	39.59
hsa-let-7e-3p	MIMAT0004485	CUAUACGGCCUCCUAGCUUUCC	125.04	144.66	96.92	102.60
hsa-let-7e-5p	MIMAT0000066	UGAGGUAGGAGGUUGUAUAGUU	78.48	67.11	89.71	77.22
hsa-let-7f-1-3p	MIMAT0004486	CUAUACAAUCUAUUGCCUUCCC	122.09	92.95	100.81	95.83
hsa-let-7f-2-3p	MIMAT0004487	CUAUACAGUCUACUGUCUUUCC	138.71	148.89	93.54	97.85
hsa-let-7f-5p	MIMAT0000067	UGAGGUAGUAGAUUGUAUAGUU	72.94	71.62	60.78	71.53
hsa-let-7g-3p	MIMAT0004584	CUGUACAGGCCACUGCCUUGC	69.96	67.38	110.83	99.48
hsa-let-7g-5p	MIMAT0000414	UGAGGUAGUAGUUUGUACAGUU	55.08	35.56	91.47	83.59
hsa-let-7i-3p	MIMAT0004585	CUGCGCAAGCUACUGCCUUGCU	86.46	92.51	108.30	108.19
hsa-let-7i-5p	MIMAT0000415	UGAGGUAGUAGUUUGUGCUGUU	35.04	32.74	64.23	51.90
hsa-miR-1	MIMAT0000416	UGGAAUGUAAAGAAGUAUGUAU	144.14	142.57	125.78	120.96
hsa-miR-100-3p	MIMAT0004512	CAAGCUUGUAUCUAUAGGUAUG	102.52	82.20	100.81	113.61
hsa-miR-100-5p	MIMAT0000098	AACCCGUAGAUCCGAACUUGUG	107.18	111.45	102.86	80.38
hsa-miR-101-3p	MIMAT0000099	UACAGUACUGUGAUAACUGAA	107.83	106.29	94.22	81.17
hsa-miR-101-5p	MIMAT0004513	CAGUUAUCACAGUGCUGAUGCU	47.51	60.73	67.47	70.52
hsa-miR-103a-2-5p	MIMAT0009196	AGCUUCUUUACAGUGCUGCCUUG	87.04	114.00	94.41	92.61
hsa-miR-103a-3p	MIMAT0000101	AGCAGCAUUGUACAGGGCUAUGA	93.20	105.24	79.17	81.24
hsa-miR-103b	MIMAT0007402	UCAUAGCCCUGUACAAUGCUGCU	91.33	92.95	125.87	122.49
hsa-miR-105-3p	MIMAT0004516	ACGGAUGUUUGAGCAUGUGCUA	162.70	201.90	107.31	122.44
hsa-miR-105-5p	MIMAT0000102	UCAAAUGCUCAGACUCCUGUGGU	89.81	80.09	82.59	115.36
hsa-miR-106a-3p	MIMAT0004517	CUGCAAUGUAAGCACUUCUUAC	100.65	91.42	71.76	71.72
hsa-miR-106a-5p	MIMAT0000103	AAAAGUGCUUACAGUGCAGGUAG	130.76	120.29	91.17	123.07
hsa-miR-106b-3p	MIMAT0004672	CCGCACUGUGGGUACUUGCUGC	42.28	41.93	101.74	71.50
hsa-miR-106b-5p	MIMAT0000680	UAAAGUGCUGACAGUGCAGAU	105.69	78.40	119.00	137.63
hsa-miR-107	MIMAT0000104	AGCAGCAUUGUACAGGGCUAUCA	152.95	152.07	93.21	120.04
hsa-miR-10a-3p	MIMAT0004555	CAAAUUCGUAUCUAGGGGAAUA	112.50	89.12	107.03	85.44
hsa-miR-10a-5p	MIMAT0000253	UACCCUGUAGAUCCGAAUUUGUG	111.97	124.33	90.01	79.79
hsa-miR-10b-3p	MIMAT0004556	ACAGAUUCGAUUCUAGGGGAAU	138.06	125.51	96.13	76.86
hsa-miR-10b-5p	MIMAT0000254	UACCCUGUAGAACCGAAUUUGUG	110.05	109.81	111.83	81.95
hsa-miR-1178-3p	MIMAT0005823	UUGCUCACUGUUCUUCCCUAG	116.70	96.58	101.86	91.31
hsa-miR-1179	MIMAT0005824	AAGCAUUCUUUCAUUGGUUGG	113.16	124.38	102.85	112.60

hsa-miR-1180	MIMAT0005825	UUUCCGGCUCGCGUGGGUGUGU	95.23	86.33	78.32	84.34
hsa-miR-1181	MIMAT0005826	CCGUCGCCGCCACCCGAGCCG	122.09	106.78	105.94	82.51
hsa-miR-1182	MIMAT0005827	GAGGGUCUUGGGAGGGAUGUGAC	138.79	128.61	126.82	128.87
hsa-miR-1183	MIMAT0005828	CACUGUAGGUGAUGGUGAGAGUGGGCA	52.19	45.32	64.36	59.02
hsa-miR-1184	MIMAT0005829	CCUGCAGCGACUUGAUGGCUUCC	144.95	126.62	95.92	122.56
hsa-miR-1184	MIMAT0005829	CCUGCAGCGACUUGAUGGCUUCC	129.95	123.14	91.66	112.83
hsa-miR-1185-5p	MIMAT0005798	AGAGGAUACCCUUUGUAUGUU	94.13	79.12	104.80	114.24
hsa-miR-1193	MIMAT0015049	GGGAUGGUAGACCGGUGACGUGC	118.45	81.94	95.19	81.56
hsa-miR-1197	MIMAT0005955	UAGGACACAUGGUCUACUUCU	113.14	88.51	119.20	99.16
hsa-miR-1200	MIMAT0005863	CUCCUGAGCCAUUCUGAGCCUC	28.99	31.74	145.08	155.96
hsa-miR-1201	MIMAT0005864	AGCCUGAUUAAACACAUGCUCUGA	82.47	111.85	83.60	78.97
hsa-miR-1202	MIMAT0005865	GUGCCAGCUGCAGUGGGGGAG	75.58	65.19	112.88	98.47
hsa-miR-1203	MIMAT0005866	CCCGGAGCCAGGAUGCAGCUC	135.30	129.60	134.57	130.80
hsa-miR-1204	MIMAT0005868	UCGUGGCCUGGUCUCCAUUAU	85.73	109.22	106.35	102.34
hsa-miR-1205	MIMAT0005869	UCUGCAGGGUUUGCUUUGAG	83.53	96.30	108.09	106.54
hsa-miR-1206	MIMAT0005870	UGUUCAUGUAGAUGUUUAAGC	62.56	77.76	63.54	58.24
hsa-miR-1207-3p	MIMAT0005872	UCAGCUGGCCCUCAUUUC	61.40	60.18	113.17	95.95
hsa-miR-1207-5p	MIMAT0005871	UGGCAGGGAGGCUGGGAGGGG	112.87	117.90	151.94	123.51
hsa-miR-1208	MIMAT0005873	UCACUGUUCAGACAGGCGGA	114.87	109.58	151.91	161.83
hsa-miR-122-3p	MIMAT0004590	AACGCCAUUAUCACACUAAAUA	114.53	99.16	87.63	104.83
hsa-miR-1224-3p	MIMAT0005459	CCCCACCUCCUCUCUCCUCAG	84.09	100.83	93.28	100.31
hsa-miR-1224-5p	MIMAT0005458	GUGAGGACUCGGGAGGUGG	86.15	78.45	104.32	102.15
hsa-miR-1225-3p	MIMAT0005573	UGAGCCCCUGUGCCGCCCCCAG	96.94	99.01	112.33	107.03
hsa-miR-1225-5p	MIMAT0005572	GUGGGUACGGCCCAGUGGGGGG	131.99	140.53	102.55	92.98
hsa-miR-122-5p	MIMAT0000421	UGGAGUGUGACAAUGGUGUUUG	123.66	141.89	94.10	115.04
hsa-miR-1226-3p	MIMAT0005577	UCACCAGCCCUGUGUUCCCUAG	96.52	85.57	98.76	104.73
hsa-miR-1226-5p	MIMAT0005576	GUGAGGGCAUGCAGGCCUGGAUGGGG	136.29	122.54	111.73	135.94
hsa-miR-1227-3p	MIMAT0005580	CGUGCCACCCUUUUCCCCAG	102.71	126.71	126.08	149.56
hsa-miR-1228-3p	MIMAT0005583	UCACACCUGCCUCGCCCCCC	166.12	150.46	140.76	137.86
hsa-miR-1228-5p	MIMAT0005582	GUGGGCGGGGGCAGGUGUGUG	101.82	104.19	103.67	155.07
hsa-miR-1229-3p	MIMAT0005584	CUCUCACCACUGCCCUCCCACAG	83.85	99.11	105.37	102.92
hsa-miR-1231	MIMAT0005586	GUGUCUGGGCGGACAGCUGC	140.49	134.25	75.53	78.78
hsa-miR-1233-3p	MIMAT0005588	UGAGCCCUGUCCUCCCGCAG	66.51	70.96	99.71	102.70
hsa-miR-1233-3p	MIMAT0005588	UGAGCCCUGUCCUCCCGCAG	76.74	64.70	109.18	101.08
hsa-miR-1234-3p	MIMAT0005589	UCGGCCUGACCACCCACCCCAC	101.30	96.12	113.06	121.44
hsa-miR-1236-3p	MIMAT0005591	CCUCUUCCCCUUGUCUCUCCAG	134.26	123.35	122.18	115.82
hsa-miR-1237-3p	MIMAT0005592	UCCUUCUGCUCCGUCCCCCAG	63.63	72.23	98.39	128.44
hsa-miR-1238-3p	MIMAT0005593	CUUCCUCGUCUGUCUGCCCC	107.18	97.60	85.01	35.11
hsa-miR-1243	MIMAT0005894	AACUGGAUCAAUUAUAGGAGUG	119.29	108.32	56.96	22.21
hsa-miR-124-3p	MIMAT0000422	UAAGGCACGCGGUGAAUGCC	85.48	81.06	100.65	109.74
hsa-miR-1244	MIMAT0005896	AAGUAGUUGGUUUGUAUGAGAUGGUU	99.83	80.53	85.69	101.62

hsa-miR-1244	MIMAT0005896	AAGUAGUUGGUUUGUAUGAGAUGGUU	73.40	91.43	91.12	106.18
hsa-miR-1245a	MIMAT0005897	AAGUGAUCUAAAGGCCUACAU	141.91	145.35	113.00	129.59
hsa-miR-124-5p	MIMAT0004591	CGUGUUCACAGCGGACCUUGAU	39.80	37.58	93.96	99.34
hsa-miR-1246	MIMAT0005898	AAUGGAUUUUUGGAGCAGG	86.87	106.17	103.77	110.50
hsa-miR-1247-5p	MIMAT0005899	ACCCGUCCCGUUCGUCCCCGGA	95.76	130.95	105.47	87.89
hsa-miR-1248	MIMAT0005900	ACCUUCUUGUAUAAGCACUGUGCUAAA	110.60	90.56	114.00	80.92
hsa-miR-1249	MIMAT0005901	ACGCCCUUCCCCCCCUUCUUCA	80.02	96.24	93.06	108.84
hsa-miR-1250	MIMAT0005902	ACGGUGCUGGAUGUGGCCUUU	71.49	61.09	110.40	94.68
hsa-miR-1251	MIMAT0005903	ACUCUAGCUGCCAAAGGCGCU	121.16	116.00	107.65	106.62
hsa-miR-1252	MIMAT0005944	AGAAGGAAAUUGAAUUCAUUUA	126.57	104.36	127.72	113.14
hsa-miR-1253	MIMAT0005904	AGAGAAGAAGAUCAGCCUGCA	102.13	80.01	72.53	84.02
hsa-miR-1254	MIMAT0005905	AGCCUGGAAGCUGGAGCCUGCAGU	49.17	59.73	83.65	99.30
hsa-miR-1255a	MIMAT0005906	AGGAUGAGCAAAGAAAGUAGAUU	88.54	80.66	107.65	118.68
hsa-miR-1255b-5p	MIMAT0005945	CGGAUGAGCAAAGAAAGUGGUU	74.20	67.39	109.32	98.34
hsa-miR-1256	MIMAT0005907	AGGCAUUGACUUCUCACUAGCU	38.12	42.15	72.95	79.25
hsa-miR-1257	MIMAT0005908	AGUGAAUGAUGGGUUCUGACC	197.57	160.70	135.85	123.64
hsa-miR-1258	MIMAT0005909	AGUUAGGAUUAGGUCGUGGAA	179.87	232.76	108.22	116.78
hsa-miR-1259	MIMAT0005910	AUAUAUGAUGACUUAGCUUUU	76.42	94.49	83.16	92.03
hsa-miR-125a-3p	MIMAT0004602	ACAGGUGAGGUUCUUGGGAGCC	66.15	67.12	104.32	117.84
hsa-miR-125a-5p	MIMAT0000443	UCCCUGAGACCCUUUAACCUGUGA	88.45	101.98	97.01	95.28
hsa-miR-125b-1-3p	MIMAT0004592	ACGGGUUAGGCUCUUGGGAGCU	110.90	129.83	87.71	102.82
hsa-miR-125b-2-3p	MIMAT0004603	UCACAAGUCAGGCUCUUGGGAC	65.00	51.04	62.15	88.87
hsa-miR-125b-5p	MIMAT0000423	UCCCUGAGACCCUAACUUGUGA	62.52	83.48	73.26	115.05
hsa-miR-1260a	MIMAT0005911	AUCCCACCUCUGCCACCA	171.48	169.77	102.52	111.07
hsa-miR-1260b	MIMAT0015041	AUCCCACCACUGCCACCAU	93.11	103.66	115.34	121.75
hsa-miR-1261	MIMAT0005913	AUGGAUAAGGCUUUGGCUU	76.42	92.95	100.24	102.82
hsa-miR-1262	MIMAT0005914	AUGGGUGAAUUUGUAGAAGGAU	66.49	63.99	96.22	79.39
hsa-miR-1263	MIMAT0005915	AUGGUACCCUGGCAUACUGAGU	102.52	103.71	129.86	137.72
hsa-miR-126-3p	MIMAT0000445	UCGUACCGUGAGUAAUAAUGCG	112.30	124.65	118.57	101.17
hsa-miR-1264	MIMAT0005791	CAAGUCUUAUUUGAGCACCUGUU	138.86	129.83	59.80	52.68
hsa-miR-1265	MIMAT0005918	CAGGAUGUGGUCAAGUGUUGUU	134.20	146.73	105.37	107.89
hsa-miR-126-5p	MIMAT0000444	CAUUAUUACUUUUGGUACGCG	113.16	90.56	103.41	78.35
hsa-miR-1266	MIMAT0005920	CCUCAGGGCUGUAGAACAGGGCU	134.15	118.21	95.23	85.17
hsa-miR-1267	MIMAT0005921	CCUGUUGAAGUGUAAUCCCCA	110.76	101.11	107.61	108.03
hsa-miR-1268a	MIMAT0005922	CGGGCGUGGUGGUGGGGG	94.84	100.76	73.66	78.84
hsa-miR-1269a	MIMAT0005923	CUGGACUGAGCCGUGCUACUGG	59.36	21.49	80.55	48.38
hsa-miR-1270	MIMAT0005924	CUGGAGAUAUGGAAGAGCUGUGU	125.32	138.58	88.91	81.83
hsa-miR-1270	MIMAT0005924	CUGGAGAUAUGGAAGAGCUGUGU	118.58	138.58	81.45	81.40
hsa-miR-1271-5p	MIMAT0005796	CUUGGCACCUAGCAAGCACUCA	99.97	110.28	82.76	115.02
hsa-miR-1272	MIMAT0005925	GAUGAUGAUGGCAGCAAAUUCUGAAA	141.70	139.97	163.23	134.14
hsa-miR-1273a	MIMAT0005926	GGGCGACAAAGCAAGACUCUUUCUU	135.71	143.44	83.52	110.63

hsa-miR-1273c	MIMAT0015017	GGCGACAAAACGAGACCCUGUC	130.63	118.27	106.82	117.69
hsa-miR-1273d	MIMAT0015090	GAACCCAUGAGGUUGAGGCUGCAGU	97.89	94.62	97.34	86.90
hsa-miR-1273e	MIMAT0018079	UUGCUUGAACCCAGGAAGUGGA	70.09	81.33	79.73	77.49
hsa-miR-127-3p	MIMAT0000446	UCGGAUCCGUCUGAGCUUGGCU	67.87	73.75	68.92	79.61
hsa-miR-1274a	MIMAT0005927	GUCCCUGUUCAGGCGCCA	89.33	96.24	94.77	111.23
hsa-miR-1274b	MIMAT0005938	UCCCUGUUCGGGCGCCA	109.93	126.62	111.55	104.07
hsa-miR-1275	MIMAT0005929	GUGGGGGAGAGGCUGUC	25.09	44.86	79.58	120.32
hsa-miR-127-5p	MIMAT0004604	CUGAAGCUCAGAGGGCUCUGAU	88.18	130.82	70.19	70.88
hsa-miR-1276	MIMAT0005930	UAAAGAGCCCUGUGGAGACA	159.21	175.34	102.31	120.83
hsa-miR-1277-3p	MIMAT0005933	UACGUAGAUAUAUAUGUAUUUU	112.99	115.43	106.71	100.13
hsa-miR-1278	MIMAT0005936	UAGUACUGUGCAUAUCAUCUAU	98.64	97.59	110.61	119.82
hsa-miR-1279	MIMAT0005937	UCAUAUUGCUUCUUUCU	169.64	159.80	117.45	112.66
hsa-miR-128	MIMAT0000424	UCACAGUGAACCGGUCUCUUU	119.68	102.74	94.52	90.63
hsa-miR-1280	MIMAT0005946	UCCCACCGCUGCCACCC	88.40	80.08	108.55	113.85
hsa-miR-1281	MIMAT0005939	UCGCCUCCUCCUCUCCC	90.29	83.83	107.03	121.36
hsa-miR-1282	MIMAT0005940	UCGUUUGCCUUUUUCUGCUU	118.45	134.23	110.53	99.21
hsa-miR-1283	MIMAT0005799	UCUACAAAGGAAAGCGCUUUCU	140.73	129.83	103.66	114.24
hsa-miR-1284	MIMAT0005941	UCUAUACAGACCCUGGCUUUUC	109.30	90.73	101.22	99.81
hsa-miR-1285-3p	MIMAT0005876	UCUGGGCAACAAAGUGAGACCU	89.47	97.56	80.31	101.55
hsa-miR-1286	MIMAT0005877	UGCAGGACCAAGAUGAGCCCU	88.16	82.27	88.36	106.93
hsa-miR-1287	MIMAT0005878	UGCUGGAUCAGUGGUUCGAGUC	100.35	99.72	100.06	123.73
hsa-miR-1288	MIMAT0005942	UGGACUGCCCUGAUCUGGAGA	125.82	105.24	84.86	78.06
hsa-miR-1289	MIMAT0005879	UGGAGUCCAGGAAUCUGCAUUUU	102.96	79.43	92.91	81.85
hsa-miR-1290	MIMAT0005880	UGGAUUUUUGGAUCAGGGA	129.92	136.08	100.39	110.04
hsa-miR-1291	MIMAT0005881	UGGCCCUGACUGAAGACCAGCAGU	125.63	98.77	126.67	110.42
hsa-miR-129-1-3p	MIMAT0004548	AAGCCCUUACCCCAAAAAGUAU	147.09	133.17	127.98	123.87
hsa-miR-129-2-3p	MIMAT0004605	AAGCCCUUACCCCAAAAAGCAU	130.20	145.87	116.12	118.43
hsa-miR-1292-5p	MIMAT0005943	UGGGAACGGGUUCCGGCAGACGCUG	114.12	114.84	156.17	136.96
hsa-miR-1293	MIMAT0005883	UGGGUGGUCUGGAGAUUUGUGC	42.19	34.36	119.24	138.84
hsa-miR-1294	MIMAT0005884	UGUGAGGUUGGCAUUGUUGUCU	36.74	33.06	62.19	72.23
hsa-miR-1295a	MIMAT0005885	UUAGGCCGCAGAUCUGGGUGA	116.97	105.37	108.17	91.69
hsa-miR-129-5p	MIMAT0000242	CUUUUUGCGGUCUGGGCUUGC	127.86	101.94	107.82	115.57
hsa-miR-1296	MIMAT0005794	UUAGGGCCCUGGCUCCAUCUCC	135.30	130.50	99.05	87.84
hsa-miR-1297	MIMAT0005886	UUCAAGUAAUUCAGGUG	67.92	63.87	80.62	83.49
hsa-miR-1298	MIMAT0005800	UUCAUUCGGCUGUCCAGAUGUA	131.10	120.16	115.11	128.44
hsa-miR-1299	MIMAT0005887	UUCUGGAAUUCUGUGUGAGGGA	177.44	151.54	96.24	102.34
hsa-miR-1300	MIMAT0005888	UUGAGAAGGAGGCUGCUG	130.16	110.81	72.64	78.04
hsa-miR-1301	MIMAT0005797	UUGCAGCUGCCUGGGAGUGACUUC	99.24	97.89	106.40	82.57
hsa-miR-1302	MIMAT0005890	UUGGGACAUACUUAUGCUAAA	98.92	120.50	76.87	108.47
hsa-miR-1302	MIMAT0005890	UUGGGACAUACUUAUGCUAAA	109.87	107.42	94.54	110.95
hsa-miR-1303	MIMAT0005891	UUUAGAGACGGGGUCUUGCUCU	130.73	124.03	112.08	128.49

hsa-miR-1304-5p	MIMAT0005892	UUUGAGGCUACAGUGAGAUGUG	67.90	64.48	93.93	95.04
hsa-miR-1305	MIMAT0005893	UUUUCAACUCUAAUGGGAGAGA	127.16	113.37	84.08	106.46
hsa-miR-1306-3p	MIMAT0005950	ACGUUGGCUCUGGUGGUG	84.02	78.02	110.74	122.94
hsa-miR-1307-3p	MIMAT0005951	ACUCGGCGUGGCGUCGGUCGUG	84.09	98.50	120.45	87.36
hsa-miR-1308	MIMAT0005947	GCAUGGGUGGUUCAGUGG	122.50	119.68	100.34	112.19
hsa-miR-130a-3p	MIMAT0000425	CAGUGCAAUGUUAAAAGGGCAU	175.16	213.42	115.12	111.32
hsa-miR-130a-5p	MIMAT0004593	UUCACAUUGUGCUACUGUCUGC	101.58	74.52	89.42	86.31
hsa-miR-130b-3p	MIMAT0000691	CAGUGCAAUGAUGAAAGGGCAU	121.07	141.58	120.69	86.88
hsa-miR-130b-5p	MIMAT0004680	ACUCUUUCCCUGUUGCACUAC	115.73	115.22	51.01	66.79
hsa-miR-1321	MIMAT0005952	CAGGGAGGUGAAUGUGAU	51.50	68.29	86.22	89.75
hsa-miR-1322	MIMAT0005953	GAUGAUGCUGCUGAUGCUG	81.08	76.05	88.85	76.16
hsa-miR-1323	MIMAT0005795	UCAAAACUGAGGGGCAUUUUCU	79.22	66.83	113.34	125.66
hsa-miR-132-3p	MIMAT0000426	UAACAGUCUACAGCCAUGGUCG	138.93	154.68	84.13	97.21
hsa-miR-1324	MIMAT0005956	CCAGACAGAAUUCUAUGCACUUUC	120.17	134.11	117.88	115.53
hsa-miR-132-5p	MIMAT0004594	ACCGUGGCUUUCGAUUGUUACU	75.49	89.88	105.94	116.14
hsa-miR-133a	MIMAT0000427	UUUGGUCCCCUUCAACCAGCUG	99.46	112.98	79.75	69.91
hsa-miR-133b	MIMAT0000770	UUUGGUCCCCUUCAACCAGCUA	142.29	185.05	118.31	104.55
hsa-miR-134	MIMAT0000447	UGUGACUGGUUGACCAGAGGGG	91.39	97.33	102.40	93.98
hsa-miR-135a-3p	MIMAT0004595	UAUAGGGAUUGGAGCCGUGGCG	124.92	128.04	96.87	84.85
hsa-miR-135a-5p	MIMAT0000428	UAUGGCUUUUUAUUCCUAUGUGA	84.81	71.44	46.70	38.71
hsa-miR-135b-3p	MIMAT0004698	AUGUAGGGCUAAAAGCCAUGGG	102.13	103.45	96.49	112.19
hsa-miR-135b-5p	MIMAT0000758	UAUGGCUUUUCAUUCCUAUGUGA	69.21	80.66	90.85	77.25
hsa-miR-136-3p	MIMAT0004606	CAUCAUCGUCUCAAAUGAGUCU	109.04	97.56	74.61	62.83
hsa-miR-136-5p	MIMAT0000448	ACUCCAUUUGUUUUGAUGAUGGA	109.99	122.90	97.74	97.25
hsa-miR-137	MIMAT0000429	UUAUUGCUUAAGAAUACGCGUAG	116.59	99.52	80.64	64.36
hsa-miR-138-1-3p	MIMAT0004607	GCUACUUCACAACACCAGGGCC	95.41	99.17	111.75	103.93
hsa-miR-138-2-3p	MIMAT0004596	GCUAUUUCACGACACCAGGGUU	100.39	84.97	93.49	108.37
hsa-miR-138-5p	MIMAT0000430	AGCUGGUGUUGUGAAUCAGGCCG	75.53	66.62	76.18	88.27
hsa-miR-139-3p	MIMAT0004552	GGAGACGCGGCCCUGUUGGAGU	78.33	86.48	124.22	117.39
hsa-miR-139-5p	MIMAT0000250	UCUACAGUGCACGUGUCUCCAG	68.75	29.95	77.76	46.24
hsa-miR-140-3p	MIMAT0004597	UACCACAGGGUAGAACCACGG	161.52	168.23	90.07	112.61
hsa-miR-140-5p	MIMAT0000431	CAGUGGUUUUACCCUAUGGUAG	106.14	122.03	93.72	79.92
hsa-miR-141-3p	MIMAT0000432	UAACACUGUCUGGUAAAGAUGG	96.55	97.06	99.61	114.49
hsa-miR-141-5p	MIMAT0004598	CAUCUUCCAGUACAGUGUUGGA	87.00	86.32	60.12	57.76
hsa-miR-142-3p	MIMAT0000434	UGUAGUGUUUCCUACUUUAUGGA	135.31	126.32	88.46	84.83
hsa-miR-142-5p	MIMAT0000433	CAUAAAGUAGAAAGCACUACU	114.35	119.68	93.92	83.06
hsa-miR-143-3p	MIMAT0000435	UGAGAUGAAGCACUGUAGCUC	97.71	121.47	83.17	103.36
hsa-miR-143-5p	MIMAT0004599	GGUGCAGUGCUGCAUCUCUGGU	123.05	106.37	80.10	89.82
hsa-miR-144-3p	MIMAT0000436	UACAGUAUAGAUGAUGUACU	161.18	167.61	82.85	83.90
hsa-miR-144-5p	MIMAT0004600	GGAUAUCAUCAUAUACUGUAAG	112.14	121.62	130.25	112.25
hsa-miR-145-3p	MIMAT0004601	GGAUUCCUGGAAAUACUGUUCU	53.83	42.58	110.06	106.57

hsa-miR-145-5p	MIMAT0000437	GUCCAGUUUUCCCAGGAAUCCCU	112.60	116.38	121.53	135.30
hsa-miR-1468	MIMAT0006789	CUCCGUUUGCCUGUUUCGCUG	73.03	65.59	84.08	100.25
hsa-miR-1469	MIMAT0007347	CUCGGCGCGGGGCGCGGGCUCC	93.26	101.48	104.82	103.18
hsa-miR-146a-3p	MIMAT0004608	CCUCUGAAAUUCAGUUCUUCAG	116.68	102.55	71.67	87.84
hsa-miR-146a-5p	MIMAT0000449	UGAGAACUGAAUUCCAUGGGUU	149.05	142.62	98.29	118.51
hsa-miR-146b-3p	MIMAT0004766	UGCCCUGUGGACUCAGUUCUGG	103.50	113.79	125.67	112.21
hsa-miR-146b-5p	MIMAT0002809	UGAGAACUGAAUUCCAUAGGCU	107.18	92.81	77.26	94.72
hsa-miR-1470	MIMAT0007348	GCCCUCCGCCCGUGCACCCCG	71.76	76.05	117.90	123.76
hsa-miR-1471	MIMAT0007349	GCCCGCGUGUGGAGCCAGGUGU	81.18	89.48	81.94	83.54
hsa-miR-147a	MIMAT0000251	GUGUGUGGAAAUGCUUCUGC	116.76	96.30	102.04	96.89
hsa-miR-147b	MIMAT0004928	GUGUGCGGAAAUGCUUCUGCUA	41.11	57.15	107.15	104.58
hsa-miR-148a-3p	MIMAT0000243	UCAGUGCACUACAGAACUUUGU	109.32	122.35	94.43	103.16
hsa-miR-148a-5p	MIMAT0004549	AAAGUUCUGAGACACUCCGACU	109.66	84.33	75.58	80.63
hsa-miR-148b-3p	MIMAT0000759	UCAGUGCAUCACAGAACUUUGU	123.55	90.26	125.26	126.32
hsa-miR-148b-5p	MIMAT0004699	AAGUUCUGUUAUACACUCAGGC	89.47	96.02	63.22	53.95
hsa-miR-149-3p	MIMAT0004609	AGGGAGGGACGGGGGCUGUGC	88.79	126.88	95.20	79.10
hsa-miR-149-5p	MIMAT0000450	UCUGGCUCCGUGUCUUCACUCCC	97.27	103.45	98.92	105.58
hsa-miR-150-3p	MIMAT0004610	CUGGUACAGGCCUGGGGGACAG	133.80	127.73	126.67	113.99
hsa-miR-150-5p	MIMAT0000451	UCUCCCAACCCUUGUACCAGUG	97.47	106.53	104.04	95.51
hsa-miR-151a-3p	MIMAT0000757	CUAGACUGAAGCUCCUUGAGG	85.74	63.76	113.91	123.13
hsa-miR-151a-5p	MIMAT0004697	UCGAGGAGCUCACAGUCUAGU	153.49	146.50	133.77	120.32
hsa-miR-152	MIMAT0000438	UCAGUGCAUGACAGAACUUGG	177.78	149.43	82.79	108.37
hsa-miR-153	MIMAT0000439	UUGCAUAGUCACAAAAGUGAUC	57.63	39.04	104.80	118.78
hsa-miR-1537	MIMAT0007399	AAAACCGUCUAGUUACAGUUGU	135.14	123.68	140.11	145.34
hsa-miR-1538	MIMAT0007400	CGGCCCGGGCUGCUGCUGUUCCU	90.29	75.90	112.08	111.85
hsa-miR-1539	MIMAT0007401	UCCUGCGCGUCCCAGAUGCCC	194.06	154.64	114.88	115.90
hsa-miR-154-3p	MIMAT0000453	AAUCAUACACGGUUGACCUAUU	113.95	74.32	84.36	87.13
hsa-miR-154-5p	MIMAT0000452	UAGGUUAUCCGUGUUGCCUUCG	84.17	100.90	89.06	87.46
hsa-miR-155-3p	MIMAT0004658	CUCCUACAUAUUAGCAUUAACA	128.61	139.04	104.23	111.07
hsa-miR-155-5p	MIMAT0000646	UUAAUGCUAAUCGUGAUAGGGGU	148.36	130.95	84.38	115.16
hsa-miR-15a-3p	MIMAT0004488	CAGGCCAUAUUGUGCUGCCUCA	110.85	116.18	77.92	63.41
hsa-miR-15a-5p	MIMAT0000068	UAGCAGCACAUAAUGGUUUGUG	104.58	85.91	73.92	87.59
hsa-miR-15b-3p	MIMAT0004586	CGAAUCAUUAUUUGCUGCUCUA	177.07	165.16	129.29	135.82
hsa-miR-15b-5p	MIMAT0000417	UAGCAGCACAUCAUGGUUUACA	69.16	88.37	86.77	82.99
hsa-miR-16-1-3p	MIMAT0004489	CCAGUAUUAACUGUGCUGCUGA	116.50	89.88	101.95	93.30
hsa-miR-16-2-3p	MIMAT0004518	CCAAUAUUACUGUGCUGCUUUA	80.60	67.44	85.79	76.46
hsa-miR-16-5p	MIMAT0000069	UAGCAGCACGUAAAUAUUGGCG	68.47	70.77	75.07	90.15
hsa-miR-17-3p	MIMAT0000071	ACUGCAGUGAAGGCACUUGUAG	73.85	80.73	109.66	103.18
hsa-miR-17-5p	MIMAT0000070	CAAAGUGCUUACAGUGCAGGUAG	95.94	87.30	85.46	102.44
hsa-miR-181a-2-3p	MIMAT0004558	ACCACUGACCGUUGACUGUACC	176.77	212.80	151.87	143.32
hsa-miR-181a-3p	MIMAT0000270	ACCAUCGACCGUUGAUUGUACC	68.79	74.76	98.54	105.59

hsa-miR-181a-5p	MIMAT0000256	AACAUUCAACGCUGUCGGUGAGU	114.26	113.40	82.66	84.47
hsa-miR-181b-5p	MIMAT0000257	AACAUUCAUUGCUGUCGGUGGGU	119.01	94.44	108.04	89.75
hsa-miR-181c-3p	MIMAT0004559	AACCAUCGACCGUUGAGUGGAC	124.58	106.81	99.90	95.48
hsa-miR-181c-5p	MIMAT0000258	AACAUUCAACCUGUCGGUGAGU	95.95	91.75	74.66	85.13
hsa-miR-181d	MIMAT0002821	AACAUUCAUUGUUGUCGGUGGGU	173.03	156.59	83.97	92.51
hsa-miR-182-3p	MIMAT0000260	UGGUUCUAGACUUGCCAACUA	75.49	76.05	82.02	93.93
hsa-miR-1825	MIMAT0006765	UCCAGUGCCCUCCUCUCC	174.60	160.38	94.82	89.07
hsa-miR-182-5p	MIMAT0000259	UUUGGCAAUGGUAGAACUCACACU	92.30	106.34	117.84	108.36
hsa-miR-1826	MIMAT0006766	AUUGAUCAUCGACACUUCGAACGCAAU	93.40	102.55	124.30	111.23
hsa-miR-1827	MIMAT0006767	UGAGGCAGUAGAUUGAAU	76.05	78.02	92.87	102.87
hsa-miR-183-3p	MIMAT0004560	GUGAAUUACCGAAGGGCCAUAA	116.30	86.44	103.67	74.59
hsa-miR-183-5p	MIMAT0000261	UAUGGCACUGGUAGAAUUCACU	106.08	90.86	112.04	93.19
hsa-miR-184	MIMAT0000454	UGGACGGAGAACUGAUAAGGGU	85.71	72.95	110.41	102.36
hsa-miR-185-3p	MIMAT0004611	AGGGGCUGGCUUUCCUCUGGUC	43.80	45.32	87.14	101.55
hsa-miR-185-5p	MIMAT0000455	UGGAGAGAAAGGCAGUUCCUGA	143.83	131.44	94.56	92.57
hsa-miR-186-3p	MIMAT0004612	GCCCAAAGGUGAAUUUUUUGGG	140.49	130.73	103.96	120.30
hsa-miR-186-5p	MIMAT0000456	CAAAGAAUUCUCCUUUUGGGCU	128.58	107.84	115.34	86.50
hsa-miR-187-3p	MIMAT0000262	UCGUGUCUUGUGUUGCAGCCGG	96.93	90.32	79.00	74.12
hsa-miR-187-5p	MIMAT0004561	GGCUACAACACAGGACCCGGGC	91.43	71.14	114.11	118.98
hsa-miR-188-3p	MIMAT0004613	CUCCCACAUGCAGGGUUUGCA	69.78	74.84	72.98	69.06
hsa-miR-188-5p	MIMAT0000457	CAUCCCUUGCAUGGUGGAGGG	130.25	129.52	83.63	79.40
hsa-miR-18a-3p	MIMAT0002891	ACUGCCCUAAGUGCUCCUUCUGG	79.51	94.85	101.41	110.02
hsa-miR-18a-5p	MIMAT0000072	UAAGGUGCAUCUAGUGCAGAUAG	100.26	126.11	142.80	163.49
hsa-miR-18b-3p	MIMAT0004751	UGCCCUAAAUGCCCCUUCUGGC	90.40	116.00	129.86	127.57
hsa-miR-18b-5p	MIMAT0001412	UAAGGUGCAUCUAGUGCAGUUAG	94.45	108.74	122.87	106.26
hsa-miR-1908	MIMAT0007881	CGGCGGGGACGGCGAUUGGUC	78.33	61.62	125.91	124.79
hsa-miR-1909-3p	MIMAT0007883	CGCAGGGGCCGGGUGCUCACCG	82.01	82.20	115.62	116.14
hsa-miR-1909-5p	MIMAT0007882	UGAGUGCCGGUGCCUGCCCUG	143.40	132.64	110.58	92.84
hsa-miR-190a	MIMAT0000458	UGAUAUGUUUGAUAUAUUAGGU	66.13	75.39	85.77	90.24
hsa-miR-190b	MIMAT0004929	UGAUAUGUUUGAUAUUGGGUU	78.15	63.78	97.83	98.47
hsa-miR-1910	MIMAT0007884	CCAGUCCUGUGCCUGCCGCCU	138.86	123.68	109.92	110.43
hsa-miR-1911-3p	MIMAT0007886	CACCAGGCAUUGUGGUCUCC	84.13	92.67	85.01	87.34
hsa-miR-1911-5p	MIMAT0007885	UGAGUACCGCCAUGUCUGUUGGG	111.36	127.21	94.56	97.06
hsa-miR-1912	MIMAT0007887	UACCCAGAGCAUGCAGUGUGAA	71.29	81.37	91.78	99.77
hsa-miR-1913	MIMAT0007888	UCUGCCCCCUCCGCUGCUGCCA	88.32	105.52	120.42	104.02
hsa-miR-191-3p	MIMAT0001618	GCUGCGCUUGGAUUUCGUCCCC	122.77	149.72	152.22	151.35
hsa-miR-1914-3p	MIMAT0007890	GGAGGGGUCCCGCACUGGGAGG	110.90	114.46	120.75	121.22
hsa-miR-1914-5p	MIMAT0007889	CCCUGUGCCCGGCCCACUUCUG	125.63	126.14	106.16	110.82
hsa-miR-1915-3p	MIMAT0007892	CCCCAGGGCGACGCGGCGGG	128.53	108.52	102.97	97.08
hsa-miR-1915-5p	MIMAT0007891	ACCUUGCCUUGCUGCCCGGGCC	81.37	77.55	95.96	67.87
hsa-miR-191-5p	MIMAT0000440	CAACGGAAUCCCAAAAGCAGCUG	94.13	84.13	131.97	96.76

hsa-miR-192-3p	MIMAT0004543	CUGCCAAUUCCAUAGGUCACAG	131.07	142.51	103.92	89.56
hsa-miR-192-5p	MIMAT0000222	CUGACCUAUGAAUUGACAGCC	93.88	102.09	91.85	86.35
hsa-miR-193a-3p	MIMAT0000459	AACUGGCCUACAAAGUCCCAGU	102.91	90.56	112.33	72.78
hsa-miR-193a-5p	MIMAT0004614	UGGGUCUUUGCGGGCGAGAUGA	114.60	115.93	106.51	105.74
hsa-miR-193b-3p	MIMAT0002819	AACUGGCCCUCAAAGUCCCGCU	81.18	77.31	115.74	85.93
hsa-miR-193b-5p	MIMAT0004767	CGGGGUUUUGAGGGCGAGAUGA	166.57	199.38	141.69	145.03
hsa-miR-194-3p	MIMAT0004671	CCAGUGGGGCUGCUGUUAUCUG	97.86	91.42	110.49	97.74
hsa-miR-194-5p	MIMAT0000460	UGUAACAGCAACUCCAUGUGGA	172.95	174.42	60.48	78.78
hsa-miR-195-3p	MIMAT0004615	CCAAUAUUGGCUGUGCUGCUCC	120.57	142.81	102.35	105.16
hsa-miR-195-5p	MIMAT0000461	UAGCAGCACAGAAAUAUUGGC	59.59	92.58	78.07	75.30
hsa-miR-196a-3p	MIMAT0004562	CGGCAACAAGAAACUGCCUGAG	114.75	108.52	122.90	120.98
hsa-miR-196a-5p	MIMAT0000226	UAGGUAGUUUCAUGUUGUUGGG	92.57	92.83	78.04	68.80
hsa-miR-196b-3p	MIMAT0009201	UCGACAGCACGACACUGCCUUC	115.52	104.36	124.30	91.66
hsa-miR-196b-5p	MIMAT0001080	UAGGUAGUUUCCUGUUGUUGGG	89.02	74.44	72.24	54.49
hsa-miR-1972	MIMAT0009447	UCAGGCCAGGCACAGUGGCUCA	134.19	146.58	109.52	117.99
hsa-miR-1972	MIMAT0009447	UCAGGCCAGGCACAGUGGCUCA	117.74	87.84	87.12	102.80
hsa-miR-1973	MIMAT0009448	ACCGUGCAAAGGUAGCAUA	112.50	89.12	99.28	89.66
hsa-miR-197-3p	MIMAT0000227	UUCACCACCUUCUCCACCCAGC	54.84	55.26	92.84	85.51
hsa-miR-1974	MIMAT0009449	UGGUUGUAGUCCGUGCGAGAAUA	119.67	101.48	105.57	112.62
hsa-miR-1975	MIMAT0009450	CCCCCACAACCGCGCUUGACUAGCU	106.24	91.42	69.49	69.18
hsa-miR-1976	MIMAT0009451	CCUCCUGCCCUCCUUGCUGU	64.48	73.64	93.93	79.20
hsa-miR-1977	MIMAT0009452	GAUUAGGGUGCUUAGCUGUUAA	82.93	90.38	85.36	110.28
hsa-miR-1978	MIMAT0009453	GGUUUGGUCCUAGCCUUUCUA	75.49	77.59	121.89	104.09
hsa-miR-1979	MIMAT0009454	CUCCCACUGCUUCACUUGACUA	141.23	137.14	96.26	124.51
hsa-miR-198	MIMAT0000228	GGUCCAGAGGGGAGAUAGGUUC	117.20	115.10	106.39	93.73
hsa-miR-199a-3p	MIMAT0000232	ACAGUAGUCUGCACAUUGGUUA	123.08	121.94	99.48	94.52
hsa-miR-199a-5p	MIMAT0000231	CCCAGUGUUCAGACUACCUGUUC	80.32	72.95	108.55	90.87
hsa-miR-199b-5p	MIMAT0000263	CCCAGUGUUUAGACUAUCUGUUC	90.49	118.78	86.22	90.23
hsa-miR-19a-3p	MIMAT0000073	UGUGCAAAUCUAUGCAAAACUGA	107.81	113.91	117.27	115.72
hsa-miR-19a-5p	MIMAT0004490	AGUUUUGCAUAGUUGCACUACA	50.23	49.19	124.73	109.32
hsa-miR-19b-1-5p	MIMAT0004491	AGUUUUGCAGGUUUGCAUCCAGC	36.55	45.32	90.42	76.21
hsa-miR-19b-2-5p	MIMAT0004492	AGUUUUGCAGGUUUGCAUUUCA	37.00	35.70	103.66	92.57
hsa-miR-19b-3p	MIMAT0000074	UGUGCAAAUCCAUGCAAAACUGA	154.62	164.41	94.91	122.60
hsa-miR-200a-3p	MIMAT0000682	UAACACUGUCUGGUAACGAUGU	82.95	80.66	110.49	92.03
hsa-miR-200a-5p	MIMAT0001620	CAUCUUACCGGACAGUGCUGGA	82.47	98.23	100.73	91.69
hsa-miR-200b-3p	MIMAT0000318	UAAUACUGCCUGGUAAUGAUGA	50.55	52.33	57.75	66.35
hsa-miR-200b-5p	MIMAT0004571	CAUCUUACUGGGCAGCAUUGGA	75.58	89.15	119.57	113.45
hsa-miR-200c-3p	MIMAT0000617	UAAUACUGCCGGGUAAUGAUGGA	82.47	83.97	61.63	63.38
hsa-miR-200c-5p	MIMAT0004657	CGUCUUACCCAGCAGUGUUUGG	108.11	120.61	102.52	107.89
hsa-miR-202-3p	MIMAT0002811	AGAGGUAUAGGGCAUGGGAA	91.09	85.98	135.72	103.64
hsa-miR-202-5p	MIMAT0002810	UUCCUAUGCAUAUACUUCUUUG	97.50	90.18	106.19	93.41

hsa-miR-203a	MIMAT0000264	GUGAAAUGUUUAGGACCACUAG	106.73	110.55	110.04	101.95
hsa-miR-204-5p	MIMAT0000265	UUCCCUUUGUCAUCCUAUGCCU	107.48	101.94	117.07	120.93
hsa-miR-2052	MIMAT0009977	UGUUUUGAUAACAGUAAUGU	40.43	35.71	100.73	92.51
hsa-miR-2053	MIMAT0009978	GUGUUAAUUAAACCUCUAUUUAC	53.83	45.30	74.24	92.61
hsa-miR-205-3p	MIMAT0009197	GAUUUCAGUGGAGUGAAGUUC	40.99	53.26	83.90	86.81
hsa-miR-2054	MIMAT0009979	CUGUAAUAUAAAUUUAAUUUAUU	102.96	104.72	106.68	98.26
hsa-miR-205-5p	MIMAT0000266	UCCUUCAUUCCACCGGAGUCUG	85.84	97.14	105.47	119.35
hsa-miR-206	MIMAT0000462	UGGAAUGUAAGGAAGUGUGUGG	143.45	121.41	80.81	73.61
hsa-miR-208a	MIMAT0000241	AUAAGACGAGCAAAAAGCUUGU	109.11	120.13	56.69	46.31
hsa-miR-208b	MIMAT0004960	AUAAGACGAACAAAAGGUUUGU	112.15	124.71	79.73	80.71
hsa-miR-20a-3p	MIMAT0004493	ACUGCAUUAUGAGCACUUAAAG	117.03	108.50	107.64	84.78
hsa-miR-20a-5p	MIMAT0000075	UAAAGUGCUUAUAGUGCAGGUAG	122.90	124.82	108.55	124.10
hsa-miR-20b-3p	MIMAT0004752	ACUGUAGUAUGGGCACUUCCAG	111.46	126.50	120.13	124.59
hsa-miR-20b-5p	MIMAT0001413	CAAAGUGCUCAUAGUGCAGGUAG	124.49	105.05	114.87	111.62
hsa-miR-210	MIMAT0000267	CUGUGCGUGUGACAGCGGCUGA	117.72	95.35	115.90	97.41
hsa-miR-2110	MIMAT0010133	UUGGGGAAACGGCCGCUGAGUG	84.81	74.52	106.51	84.41
hsa-miR-2113	MIMAT0009206	AUUUGUGCUUGGCUCUGUCAC	146.94	177.83	120.02	118.87
hsa-miR-2114-3p	MIMAT0011157	CGAGCCUCAAGCAAGGGACUU	78.42	79.72	77.84	82.63
hsa-miR-2114-5p	MIMAT0011156	UAGUCCCUUCCUUGAAGCGGUC	97.35	108.45	86.13	84.46
hsa-miR-2115-3p	MIMAT0011159	CAUCAGAAUUCAUGGAGGCUAG	67.88	70.54	85.66	79.24
hsa-miR-2115-5p	MIMAT0011158	AGCUUCCAUGACUCCUGAUGGA	71.96	66.09	97.82	82.85
hsa-miR-211-5p	MIMAT0000268	UUCCCUUUGUCAUCCUUCGCCU	112.02	92.64	93.49	101.68
hsa-miR-2116-3p	MIMAT0011161	CCUCCCAUGCCAAGAACUCCC	89.02	77.22	87.87	79.21
hsa-miR-2116-5p	MIMAT0011160	GGUUCUUAGCAUAGGAGGUCU	76.57	78.94	85.09	128.63
hsa-miR-2117	MIMAT0011162	UGUUCUCUUUGCCAAGGACAG	85.49	92.42	117.80	112.80
hsa-miR-212-3p	MIMAT0000269	UAACAGUCUCCAGUCACGGCC	105.69	107.21	62.78	81.69
hsa-miR-21-3p	MIMAT0004494	CAACACCAGUCGAUGGGCUGU	86.67	83.73	97.39	89.49
hsa-miR-214-3p	MIMAT0000271	ACAGCAGGCACAGACAGGCAGU	113.07	138.59	86.42	93.32
hsa-miR-214-5p	MIMAT0004564	UGCCUGUCUACACUUGCUGUGC	121.57	78.40	94.27	79.61
hsa-miR-215	MIMAT0000272	AUGACCUAUGAAUUGACAGAC	90.86	96.00	107.37	116.87
hsa-miR-21-5p	MIMAT0000076	UAGCUUAUCAGACUGAUGUUGA	110.59	121.46	112.80	105.38
hsa-miR-216a-5p	MIMAT0000273	UAAUCUCAGCUGGCAACUGUGA	116.50	124.28	96.76	99.23
hsa-miR-216b	MIMAT0004959	AAAUCUCUGCAGGCAAAUGUGA	169.78	172.43	116.53	118.25
hsa-miR-217	MIMAT0000274	UACUGCAUCAGGAACUGAUUGGA	109.75	99.72	64.39	80.92
hsa-miR-218-1-3p	MIMAT0004565	AUGGUUCCGUCAAGCACCAUGG	91.81	110.29	72.19	62.93
hsa-miR-218-2-3p	MIMAT0004566	CAUGGUUCUGUCAAGCACCGCG	112.15	106.28	89.33	107.52
hsa-miR-218-5p	MIMAT0000275	UUGUGCUUGAUCUAACCAUGU	83.07	86.29	99.94	99.21
hsa-miR-219-1-3p	MIMAT0004567	AGAGUUGAGUCUGGACGUCCCG	78.15	99.72	111.21	116.45
hsa-miR-219-2-3p	MIMAT0004675	AGAAUUGUGGCUGGACAUCUGU	49.33	57.21	138.44	141.06
hsa-miR-219-5p	MIMAT0000276	UGAUUGUCCAAACGCAAUUCU	84.15	98.59	73.01	90.63
hsa-miR-220a	MIMAT0000277	CCACACCGUAUCUGACACUUU	80.32	93.05	105.19	91.69

hsa-miR-220b	MIMAT0004908	CCACCACCGUGUCUGACACUU	92.26	82.20	105.94	104.09
hsa-miR-221-3p	MIMAT0000278	AGCUACAUUGUCUGCUGGGUUUC	142.31	139.73	110.78	120.82
hsa-miR-221-5p	MIMAT0004568	ACCUGGCAUACAAUGUAGAUUU	108.04	92.67	97.28	104.46
hsa-miR-222-3p	MIMAT0000279	AGCUACAUCUGGCUACUGGGU	97.75	118.23	79.09	87.81
hsa-miR-222-5p	MIMAT0004569	CUCAGUAGCCAGUGUAGAUCCU	45.34	106.70	89.53	91.03
hsa-miR-223-3p	MIMAT0000280	UGUCAGUUUGUCAAAUACCCCA	113.82	98.12	82.72	76.97
hsa-miR-223-5p	MIMAT0004570	CGUGUAUUUGACAAGCUGAGUU	83.86	74.16	83.23	82.97
hsa-miR-22-3p	MIMAT0000077	AAGCUGCCAGUUGAAGAACUGU	56.06	43.12	78.01	67.08
hsa-miR-224-3p	MIMAT0009198	AAAAUGGUGCCCUAGUGACUACA	65.71	61.75	99.32	110.46
hsa-miR-224-5p	MIMAT0000281	CAAGUCACUAGUGGUUCCGUU	76.04	63.08	109.42	104.22
hsa-miR-22-5p	MIMAT0004495	AGUUCUUCAGUGGCAAGCUUUA	90.29	103.40	85.12	117.66
hsa-miR-2276	MIMAT0011775	UCUGCAAGUGUCAGAGGCGAGG	89.12	89.44	98.34	105.48
hsa-miR-2277-3p	MIMAT0011777	UGACAGCGCCCUGCCUGGCUC	103.84	120.29	120.76	115.88
hsa-miR-2277-5p	MIMAT0017352	AGCGCGGGCUGAGCGCUGCCAGUC	62.31	46.63	95.20	82.85
hsa-miR-2278	MIMAT0011778	GAGAGCAGUGUGUGUUGCCUGG	101.10	126.54	96.74	97.36
hsa-miR-2355-3p	MIMAT0017950	AUUGUCCUUGCUGUUUGGAGAU	89.56	97.60	120.80	114.49
hsa-miR-2355-5p	MIMAT0016895	AUCCCCAGAUACAAUGGACAA	144.95	144.71	103.03	119.72
hsa-miR-23a-3p	MIMAT0000078	AUCACAUUGCCAGGGAUUUCC	123.02	117.53	73.47	88.85
hsa-miR-23a-5p	MIMAT0004496	GGGGUUCCUGGGGAUGGGAUUU	120.64	136.43	88.90	97.80
hsa-miR-23b-3p	MIMAT0000418	AUCACAUUGCCAGGGAUUACC	103.77	112.40	116.79	98.04
hsa-miR-23b-5p	MIMAT0004587	UGGGUUCCUGGCAUGCUGAUUU	106.45	78.96	77.14	73.53
hsa-miR-23c	MIMAT0018000	AUCACAUUGCCAGUGAUUACCC	85.10	101.04	75.80	90.37
hsa-miR-24-1-5p	MIMAT0000079	UGCCUACUGAGCUGAUAUCAGU	97.27	78.96	96.88	84.56
hsa-miR-24-2-5p	MIMAT0004497	UGCCUACUGAGCUGAAACACAG	93.84	96.75	83.13	84.17
hsa-miR-24-3p	MIMAT0000080	UGGCUCAGUUCAGCAGGAACAG	93.28	105.52	106.94	125.15
hsa-miR-25-3p	MIMAT0000081	CAUUGCACUUGUCUCGGUCUGA	63.24	65.68	97.73	78.77
hsa-miR-25-5p	MIMAT0004498	AGGCGGAGACUUGGGCAAUUG	99.22	93.27	91.90	110.65
hsa-miR-26a-1-3p	MIMAT0004499	CCUAUUCUUGGUUACUUGCACG	93.69	103.54	103.25	107.42
hsa-miR-26a-2-3p	MIMAT0004681	CCUAUUCUUGAUUACUUGUUUC	110.79	88.07	100.29	86.49
hsa-miR-26a-5p	MIMAT0000082	UUCAAGUAAUCCAGGAUAGGCU	113.07	120.86	99.95	80.42
hsa-miR-26b-3p	MIMAT0004500	CCUGUUCUCCAUUACUUGGCUC	98.64	91.73	82.79	105.50
hsa-miR-26b-5p	MIMAT0000083	UUCAAGUAAUUCAGGAUAGGU	70.46	91.93	76.84	105.02
hsa-miR-27a-3p	MIMAT0000084	UUCACAGUGGCUAAGUUCCGC	170.19	144.71	111.55	127.42
hsa-miR-27a-5p	MIMAT0004501	AGGGCUUAGCUGCUUGUGAGCA	112.61	89.93	128.15	121.73
hsa-miR-27b-3p	MIMAT0000419	UUCACAGUGGCUAAGUUCUGC	132.98	149.59	104.30	89.81
hsa-miR-27b-5p	MIMAT0004588	AGAGCUUAGCUGAUUGGUGAAC	98.79	112.92	95.12	104.72
hsa-miR-28-3p	MIMAT0004502	CACUAGAUUGUGAGCUCCUGGA	54.99	26.89	111.63	94.57
hsa-miR-28-5p	MIMAT0000085	AAGGAGCUCACAGUCUAUUGAG	40.74	34.42	73.17	72.82
hsa-miR-2861	MIMAT0013802	GGGGCCUGGCGGUGGGCGG	66.92	88.91	100.30	94.96
hsa-miR-2909	MIMAT0013863	GUUAGGGCCAACAUCUCUUGG	78.10	95.31	103.97	79.61
hsa-miR-296-3p	MIMAT0004679	GAGGGUUGGGUGGAGGCUCUCC	100.76	100.24	95.92	88.42

hsa-miR-296-5p	MIMAT0000690	AGGGCCCCCCCUCAAUCCUGU	75.42	93.65	95.40	99.61
hsa-miR-297	MIMAT0004450	AUGUAUGUGUGCAUGUGCAUG	60.75	60.73	84.53	79.10
hsa-miR-298	MIMAT0004901	AGCAGAAGCAGGGAGGUUCUCCCA	84.61	98.50	108.71	97.57
hsa-miR-299-3p	MIMAT0000687	UAUGUGGGAUGGUAAACCGCUU	128.60	104.32	99.51	110.03
hsa-miR-299-5p	MIMAT0002890	UGGUUUACCGUCCCACAUACAU	96.31	85.59	94.84	82.38
hsa-miR-29a-3p	MIMAT0000086	UAGCACCAUCUGAAAUCGGUUA	54.08	79.25	67.99	84.28
hsa-miR-29a-5p	MIMAT0004503	ACUGAUUUCUUUUGGUGUUCAG	112.31	125.79	110.65	129.72
hsa-miR-29b-1-5p	MIMAT0004514	GCUGGUUUCAUAUGGUGGUUUAGA	93.52	101.83	125.15	124.59
hsa-miR-29b-2-5p	MIMAT0004515	CUGGUUUCACAUGGUGGCUUAG	74.65	61.98	108.65	97.69
hsa-miR-29b-3p	MIMAT0000100	UAGCACCAUUUGAAAUCAGUGUU	118.09	132.64	101.97	72.19
hsa-miR-29c-3p	MIMAT0000681	UAGCACCAUUUGAAAUCGGUUA	73.93	91.96	44.61	55.13
hsa-miR-29c-5p	MIMAT0004673	UGACCGAUUUCUCCUGGUGUUC	95.15	90.44	113.92	113.19
hsa-miR-300	MIMAT0004903	UAUACAAGGGCAGACUCUCUCU	126.73	137.37	97.91	116.74
hsa-miR-301a-3p	MIMAT0000688	CAGUGCAAUAGUAUUGUCAAAGC	86.15	103.73	113.09	93.98
hsa-miR-301b	MIMAT0004958	CAGUGCAAUGAUAUUGUCAAAGC	200.53	207.16	116.74	113.85
hsa-miR-302a-3p	MIMAT0000684	UAAGUGCUUCCAUGUUUUGGUGA	123.05	121.62	146.53	148.85
hsa-miR-302a-5p	MIMAT0000683	ACUUAAACGUGGAUGUACUUGCU	120.90	72.56	107.17	98.25
hsa-miR-302b-3p	MIMAT0000715	UAAGUGCUUCCAUGUUUUAGUAG	123.02	126.75	129.86	126.30
hsa-miR-302b-5p	MIMAT0000714	ACUUUAACAUGGAAGUGCUUUC	88.64	86.87	96.20	93.50
hsa-miR-302c-3p	MIMAT0000717	UAAGUGCUUCCAUGUUUCAGUGG	185.42	233.52	130.29	123.47
hsa-miR-302c-5p	MIMAT0000716	UUUAACAUGGGGGUACCUGCUG	116.50	106.78	112.77	109.16
hsa-miR-302d-3p	MIMAT0000718	UAAGUGCUUCCAUGUUUGAGUGU	89.25	89.85	134.07	144.28
hsa-miR-302d-5p	MIMAT0004685	ACUUUAACAUGGAGGCACUUGC	92.67	115.93	71.08	76.21
hsa-miR-302e	MIMAT0005931	UAAGUGCUUCCAUGCUU	146.11	139.37	143.10	133.24
hsa-miR-302f	MIMAT0005932	UAAUUGCUUCCAUGUUU	118.05	99.53	82.11	105.64
hsa-miR-3065-3p	MIMAT0015378	UCAGCACCAGGAUAUUGUUGGAG	90.69	78.54	99.51	91.43
hsa-miR-3065-5p	MIMAT0015066	UCAACAAAAUCACUGAUGCUGGA	128.50	114.95	108.00	122.54
hsa-miR-3074-3p	MIMAT0015027	GAUAUCAGCUCAGUAGGCACCG	42.83	52.05	85.07	65.69
hsa-miR-30a-3p	MIMAT0000088	CUUUCAGUCGGAUGUUUGCAGC	129.95	118.27	69.39	81.23
hsa-miR-30a-5p	MIMAT0000087	UGUAAACAUCCUCGACUGGAAG	94.27	78.71	104.53	98.77
hsa-miR-30b-3p	MIMAT0004589	CUGGGAGGUGGAUGUUUACUUC	118.82	128.78	102.76	80.18
hsa-miR-30b-5p	MIMAT0000420	UGUAAACAUCCUACACUCAGCU	118.05	104.07	107.06	112.21
hsa-miR-30c-1-3p	MIMAT0004674	CUGGGAGAGGGUUGUUUACUCC	102.06	104.65	134.62	121.16
hsa-miR-30c-2-3p	MIMAT0004550	CUGGGAGAAGGCUGUUUACUCU	121.07	107.99	110.42	127.52
hsa-miR-30c-5p	MIMAT0000244	UGUAAACAUCCUACACUCUCAGC	62.44	55.93	81.77	93.80
hsa-miR-30d-3p	MIMAT0004551	CUUUCAGUCAGAUGUUUGCUGC	102.91	120.86	86.13	83.06
hsa-miR-30d-5p	MIMAT0000245	UGUAAACAUCCCCGACUGGAAG	69.99	78.45	119.30	109.34
hsa-miR-30e-3p	MIMAT0000693	CUUUCAGUCGGAUGUUUACAGC	104.62	27.13	78.88	44.10
hsa-miR-30e-5p	MIMAT0000692	UGUAAACAUCCUUGACUGGAAG	101.04	89.22	119.54	109.60
hsa-miR-3115	MIMAT0014977	AUAUGGGUUUACUAGUUGGU	81.25	117.34	68.31	90.93
hsa-miR-3116	MIMAT0014978	UGCCUGGAACAUAGUAGGGACU	84.89	82.42	109.60	137.02

hsa-miR-3117-3p	MIMAT0014979	AUAGGACUCAUAUAGUGCCAG	85.77	97.58	84.64	83.11
hsa-miR-3118	MIMAT0014980	UGUGACUGCAUUAUGAAAAUUCU	108.26	69.40	112.27	101.62
hsa-miR-3119	MIMAT0014981	UGGCUUUUAACUUUGAUGGC	66.25	61.63	86.07	104.30
hsa-miR-3120-3p	MIMAT0014982	CACAGCAAGUGUAGACAGGCA	97.91	109.31	122.03	114.28
hsa-miR-3121-3p	MIMAT0014983	UAAAUAGAGUAGGCAAAGGACA	125.85	115.49	71.29	76.78
hsa-miR-3122	MIMAT0014984	GUUGGGACAAGAGGACGGUCUU	76.87	107.66	97.28	96.58
hsa-miR-3123	MIMAT0014985	CAGAGAAUUGUUUAAUC	175.93	142.40	127.65	101.27
hsa-miR-3124-5p	MIMAT0014986	UUCGCGGGCGAAGGCAAAGUC	92.68	101.94	120.80	108.06
hsa-miR-3125	MIMAT0014988	UAGAGGAAGCUGUGGAGAGA	100.35	103.93	97.84	95.78
hsa-miR-3126-3p	MIMAT0015377	CAUCUGGCAUCCGUCACACAGA	89.56	107.36	100.00	73.74
hsa-miR-3126-5p	MIMAT0014989	UGAGGGACAGAUGCCAGAAGCA	121.87	115.42	101.78	105.09
hsa-miR-3127-5p	MIMAT0014990	AUCAGGGCUUGUGGAAUGGGAAG	109.22	112.44	124.42	104.76
hsa-miR-3128	MIMAT0014991	UCUGGCAAGUAAAAAACUCUCAU	65.20	78.81	107.64	121.30
hsa-miR-3129-5p	MIMAT0014992	GCAGUAGUGUAGAGAUUGGUUU	117.45	127.33	82.32	79.95
hsa-miR-3130-3p	MIMAT0014994	GCUGCACCGGAGACUGGGUAA	79.22	72.37	100.31	107.81
hsa-miR-3130-5p	MIMAT0014995	UACCCAGUCUCCGGUGCAGCC	80.28	89.43	95.97	93.77
hsa-miR-3131	MIMAT0014996	UCGAGGACUGGUGGAAGGGCCUU	83.26	124.16	115.03	102.51
hsa-miR-3132	MIMAT0014997	UGGGUAGAGAAGGAGCUCAGAGGA	93.66	90.84	95.07	101.72
hsa-miR-3133	MIMAT0014998	UAAAGAACUCUUAAAACCCAAU	60.02	68.42	75.39	78.60
hsa-miR-3134	MIMAT0015000	UGAUGGAUAAAAGACUACAUAUU	86.91	99.64	88.12	110.22
hsa-miR-3135a	MIMAT0015001	UGCCUAGGCUGAGACUGCAGUG	84.81	112.63	85.66	101.23
hsa-miR-3136-5p	MIMAT0015003	CUGACUGAAUAGGUAGGGUCAUU	78.12	82.82	97.29	119.72
hsa-miR-3137	MIMAT0015005	UCUGUAGCCUGGGAGCAAUGGGGU	95.35	78.96	140.39	138.44
hsa-miR-3138	MIMAT0015006	UGUGGACAGUGAGGUAGAGGGAGU	70.84	65.69	106.19	114.43
hsa-miR-3139	MIMAT0015007	UAGGAGCUCAACAGAUGCCUGUU	84.11	60.73	65.33	61.94
hsa-miR-31-3p	MIMAT0004504	UGCUAUGCCAACAUAUUGCCAU	110.60	106.77	112.33	139.14
hsa-miR-3140-3p	MIMAT0015008	AGCUUUUGGGAAUUCAGGUAGU	85.77	99.88	93.82	111.38
hsa-miR-3141	MIMAT0015010	GAGGGCGGGUGGAGGAGGA	92.30	88.04	99.57	116.86
hsa-miR-3142	MIMAT0015011	AAGGCCUUUCUGAACCUUCAGA	101.98	77.22	124.81	83.66
hsa-miR-3143	MIMAT0015012	AUAACAUUGUAAAGCGCUUCUUUCG	130.06	146.40	114.93	126.83
hsa-miR-3144-3p	MIMAT0015015	AUAUACCUGUUCGGUCUCUUUA	125.38	120.28	84.98	118.38
hsa-miR-3144-5p	MIMAT0015014	AGGGGACCAAAGAGAUAUAUAG	73.21	65.07	76.00	76.95
hsa-miR-3145-3p	MIMAT0015016	AGAUAUUUUGAGUGUUUGGAAUUG	79.06	105.20	82.01	95.87
hsa-miR-3146	MIMAT0015018	CAUGCUAGGAUAGAAAGAAUGG	98.13	121.46	84.00	87.14
hsa-miR-3147	MIMAT0015019	GGUUGGGCAGUGAGGAGGGUGUGA	77.69	82.76	121.40	115.60
hsa-miR-3148	MIMAT0015021	UGGAAAAAACUGGUGUGUGCUU	108.63	126.76	133.10	128.28
hsa-miR-3149	MIMAT0015022	UUUGUAUGGAUAUGUGUGUGUAU	118.82	107.26	78.23	88.35
hsa-miR-3150a-3p	MIMAT0015023	CUGGGGAGAUCCUCGAGGUUGG	59.00	75.14	111.55	118.10
hsa-miR-3150b-3p	MIMAT0018194	UGAGGAGAUCGUCGAGGUUGG	90.38	112.71	99.24	123.77
hsa-miR-3151	MIMAT0015024	GGUGGGGCAAUGGGAUCAGGU	86.15	75.83	103.59	92.02
hsa-miR-3152-3p	MIMAT0015025	UGUGUUAGAAUAGGGGCAAUAA	121.87	104.72	100.62	112.83

hsa-miR-3153	MIMAT0015026	GGGGAAAGCGAGUAGGGACAUUU	164.33	134.47	135.73	134.87
hsa-miR-3154	MIMAT0015028	CAGAAGGGGAGUUGGGAGCAGA	87.59	88.70	83.14	87.96
hsa-miR-3155a	MIMAT0015029	CCAGGCUCUGCAGUGGGAACU	150.31	129.05	114.93	120.93
hsa-miR-3156-5p	MIMAT0015030	AAAGAUCUGGAAGUGGGAGACA	104.81	109.88	102.28	89.45
hsa-miR-3157-5p	MIMAT0015031	UUCAGCCAGGCUAGUGCAGUCU	82.20	69.57	82.66	85.28
hsa-miR-3158-3p	MIMAT0015032	AAGGGCUUCCUCUCUGCAGGAC	106.21	102.17	98.92	114.52
hsa-miR-3159	MIMAT0015033	UAGGAUUACAAGUGUCGGCCAC	164.05	180.89	114.39	130.66
hsa-miR-31-5p	MIMAT0000089	AGGCAAGAUGCUGGCAUAGCU	84.58	86.58	133.23	137.00
hsa-miR-3160-3p	MIMAT0015034	AGAGCUGAGACUAGAAAGCCCA	132.73	116.71	98.24	93.50
hsa-miR-3161	MIMAT0015035	CUGAUAAGAACAGAGGCCCAGAU	84.74	85.68	90.08	92.22
hsa-miR-3162-5p	MIMAT0015036	UUAGGGAGUAGAAGGGUGGGGAG	86.91	106.34	116.01	87.77
hsa-miR-3163	MIMAT0015037	UAUAAAAUGAGGGCAGUAAGAC	97.53	124.25	125.86	124.01
hsa-miR-3164	MIMAT0015038	UGUGACUUUAAGGGAAAUGGCG	101.11	117.37	108.67	104.62
hsa-miR-3165	MIMAT0015039	AGGUGGAUGCAAUGUGACCUCA	42.63	46.61	61.34	75.15
hsa-miR-3166	MIMAT0015040	CGCAGACAAUGCCUACUGGCCUA	75.38	82.09	93.08	76.78
hsa-miR-3167	MIMAT0015042	AGGAUUUCAGAAAUACUGGUGU	91.78	108.28	84.29	105.46
hsa-miR-3168	MIMAT0015043	GAGUUCUACAGUCAGAC	111.67	84.98	100.56	90.23
hsa-miR-3169	MIMAT0015044	UAGGACUGUGCUUGGCACAUAG	56.96	52.87	119.13	116.48
hsa-miR-3170	MIMAT0015045	CUGGGGUUCUGAGACAGACAGU	133.06	143.82	139.03	151.18
hsa-miR-3171	MIMAT0015046	AGAUGUAUGGAAUCUGUAUAUAUC	85.29	92.31	93.26	101.67
hsa-miR-3173-3p	MIMAT0015048	AAAGGAGGAAAUAGGCAGGCCA	73.28	81.16	101.82	102.79
hsa-miR-3174	MIMAT0015051	UAGUGAGUUAGAGAUGCAGAGCC	60.53	76.59	92.20	98.05
hsa-miR-3175	MIMAT0015052	CGGGGAGAGAACGCAGUGACGU	81.51	95.31	112.50	94.60
hsa-miR-3176	MIMAT0015053	ACUGGCCUGGGACUACCGG	125.63	97.58	126.67	111.61
hsa-miR-3177-3p	MIMAT0015054	UGCACGGCACUGGGGACACGU	28.82	37.96	92.00	82.32
hsa-miR-3178	MIMAT0015055	GGGGCGCGGCCGGAUCG	95.97	97.61	119.44	119.88
hsa-miR-3179	MIMAT0015056	AGAAGGGGUGAAAUUUAAACGU	81.94	88.40	83.28	102.68
hsa-miR-3180	MIMAT0018178	UGGGGCGGAGCUUCCGGAG	73.63	113.65	105.04	116.69
hsa-miR-3180-3p	MIMAT0015058	UGGGGCGGAGCUUCCGGAGGCC	91.12	72.66	121.33	137.02
hsa-miR-3180-5p	MIMAT0015057	CUUCCAGACGCUCCGCCCCACGUCG	88.36	103.54	101.78	105.09
hsa-miR-3181	MIMAT0015061	AUCGGGCCCUCGGCGCCGG	54.91	60.53	118.18	78.40
hsa-miR-3182	MIMAT0015062	GCUUCUGUAGUGUAGUC	134.06	152.53	117.47	102.36
hsa-miR-3183	MIMAT0015063	GCCUCUCUCGGAGUCGCUCGGA	113.73	109.08	122.79	117.15
hsa-miR-3184-5p	MIMAT0015064	UGAGGGGCCUCAGACCGAGCUUUU	65.14	53.57	103.50	95.82
hsa-miR-3185	MIMAT0015065	AGAAGAAGGCGGUCGGUCUGCGG	138.13	153.06	104.45	120.13
hsa-miR-3186-3p	MIMAT0015068	UCACGCGGAGAGAUGGCUUUG	59.19	41.21	85.60	91.97
hsa-miR-3186-5p	MIMAT0015067	CAGGCGUCUGUCUACGUGGCUU	72.11	99.17	85.09	77.57
hsa-miR-3187-3p	MIMAT0015069	UUGGCCAUGGGGCUGCGCGG	93.46	124.71	108.53	131.65
hsa-miR-3188	MIMAT0015070	AGAGGCUUUGUGCGGAUACGGGG	131.77	107.26	106.19	90.30
hsa-miR-3189-3p	MIMAT0015071	CCCUUGGGUCUGAUGGGGUAG	88.45	64.50	95.55	84.50
hsa-miR-3190	MIMAT0015073	UGUGGAAGGUAGACGGCCAGAGA	123.81	131.49	125.29	108.38

hsa-miR-3191-3p	MIMAT0015075	UGGGGACGUAGCUGGCCAGACAG	95.16	88.36	112.97	107.16
hsa-miR-3192	MIMAT0015076	UCUGGGAGGUUGUAGCAGUGGAA	92.30	71.48	70.70	61.29
hsa-miR-3193	MIMAT0015077	UCCUGCGUAGGAUCUGAGGAGU	74.69	98.79	87.39	113.64
hsa-miR-3194-5p	MIMAT0015078	GGCCAGCCACCAGGAGGGCUG	127.73	108.45	81.87	64.62
hsa-miR-3195	MIMAT0015079	CGCGCCGGGCCCGGGUU	111.97	108.00	95.89	82.13
hsa-miR-3196	MIMAT0015080	CGGGGCGGCAGGGGCCUC	95.16	88.36	98.29	114.86
hsa-miR-3197	MIMAT0015082	GGAGGCGCAGGCUCGGAAAGGCG	94.56	108.13	138.78	127.39
hsa-miR-3198	MIMAT0015083	GUGGAGUCCUGGGGAAUGGAGA	151.77	180.89	83.60	71.91
hsa-miR-3199	MIMAT0015084	AGGGACUGCCUUAGGAGAAAGUU	121.87	116.46	94.50	89.87
hsa-miR-3200-3p	MIMAT0015085	CACCUUGCGCUACUCAGGUCUG	89.95	115.43	82.88	93.80
hsa-miR-3200-5p	MIMAT0017392	AAUCUGAGAAGGCGCACAAGGU	83.03	74.48	108.48	102.55
hsa-miR-3201	MIMAT0015086	GGGAUAUGAAGAAAAAU	117.60	123.63	113.33	111.27
hsa-miR-3202	MIMAT0015089	UGGAAGGGAGAAGAGCUUUAAU	125.70	99.88	98.58	97.85
hsa-miR-320a	MIMAT0000510	AAAAGCUGGGUUGAGAGGGCGA	70.62	68.41	98.12	85.54
hsa-miR-320b	MIMAT0005792	AAAAGCUGGGUUGAGAGGGCAA	120.48	111.87	127.59	135.09
hsa-miR-320c	MIMAT0005793	AAAAGCUGGGUUGAGAGGGU	86.83	98.74	97.88	123.20
hsa-miR-320d	MIMAT0006764	AAAAGCUGGGUUGAGAGGA	112.50	122.45	92.20	78.84
hsa-miR-320e	MIMAT0015072	AAAGCUGGGUUGAGAAGG	118.23	114.14	105.12	115.34
hsa-miR-323a-3p	MIMAT0000755	CACAUUACACGGUCGACCUCU	73.44	86.87	69.63	81.51
hsa-miR-323a-5p	MIMAT0004696	AGGUGGUCCGUGGCGCGUUCGC	85.38	91.52	92.99	90.38
hsa-miR-323b-3p	MIMAT0015050	CCCAAUACACGGUCGACCUCUU	69.58	52.18	79.91	74.92
hsa-miR-323b-5p	MIMAT0001630	AGGUUGUCCGUGGUGAGUUCGCA	107.40	90.18	111.59	97.69
hsa-miR-32-3p	MIMAT0004505	CAAUUUAGUGUGUGUGAUAUUU	112.02	128.25	112.75	123.17
hsa-miR-324-3p	MIMAT0000762	ACUGCCCCAGGUGCUGCUGG	96.55	117.16	101.63	109.47
hsa-miR-324-5p	MIMAT0000761	CGCAUCCCCUAGGGCAUUGGUGU	74.34	82.25	103.99	83.06
hsa-miR-325	MIMAT0000771	CCUAGUAGGUGUCCAGUAAGUGU	94.39	84.31	109.45	101.47
hsa-miR-32-5p	MIMAT0000090	UAUUGCACAUUACUAAGUUGCA	86.67	74.03	99.25	88.63
hsa-miR-326	MIMAT0000756	CCUCUGGGCCCUUCCUCCAG	76.41	97.04	78.06	61.68
hsa-miR-328	MIMAT0000752	CUGGCCCUCUCUGCCCUUCCGU	183.06	180.43	84.95	103.13
hsa-miR-329	MIMAT0001629	AACACACCUGGUUAACCUCUUU	76.53	71.45	82.37	98.82
hsa-miR-330-3p	MIMAT0000751	GCAAAGCACACGGCCUGCAGAGA	140.42	154.18	105.68	113.43
hsa-miR-330-5p	MIMAT0004693	UCUCUGGGCCUGUGUCUUAGGC	102.21	102.13	141.60	121.45
hsa-miR-331-3p	MIMAT0000760	GCCCCUGGGCCUAUCCUAGAA	112.24	130.82	125.31	99.03
hsa-miR-331-5p	MIMAT0004700	CUAGGUAUGGUCCCAGGGAUCC	107.42	110.88	94.68	78.28
hsa-miR-335-3p	MIMAT0004703	UUUUUCAUUAUUGCUCCUGACC	81.56	85.62	87.24	78.35
hsa-miR-335-5p	MIMAT0000765	UCAAGAGCAAUAACGAAAAAUGU	84.55	86.47	116.49	114.82
hsa-miR-337-3p	MIMAT0000754	CUCCUAUAUGAUGCCUUUCUUC	91.12	101.94	113.33	110.74
hsa-miR-337-5p	MIMAT0004695	GAACGGCUUCAUACAGGAGUU	127.73	109.53	102.13	111.81
hsa-miR-338-3p	MIMAT0000763	UCCAGCAUCAGUGAUUUUGUUG	110.65	124.17	77.06	105.17
hsa-miR-338-5p	MIMAT0004701	AACAAUAUCCUGGUGCUGAGUG	98.24	83.48	87.19	82.18
hsa-miR-339-3p	MIMAT0004702	UGAGCGCCUCGACGACAGAGCCG	121.51	95.78	73.14	78.33

hsa-miR-339-5p	MIMAT0000764	UCCCUGUCCUCCAGGAGCUCACG	122.90	128.71	79.13	85.54
hsa-miR-33a-3p	MIMAT0004506	CAAUGUUUCCACAGUGCAUCAC	72.94	78.40	80.46	70.81
hsa-miR-33a-5p	MIMAT0000091	GUGCAUUGUAGUUGCAUUGCA	105.87	110.97	65.48	65.39
hsa-miR-33b-3p	MIMAT0004811	CAGUGCCUCGGCAGUGCAGCCC	98.24	111.45	100.67	100.76
hsa-miR-33b-5p	MIMAT0003301	GUGCAUUGCUGUUGCAUUGC	75.23	113.91	76.14	69.34
hsa-miR-340-3p	MIMAT0000750	UCCGUCUCAGUUACUUUAUAGC	111.36	121.39	103.66	113.70
hsa-miR-340-5p	MIMAT0004692	UUAUAAAGCAAUGAGACUGAUU	73.74	88.44	141.77	110.64
hsa-miR-342-3p	MIMAT0000753	UCUCACACAGAAAUCGCACCCGU	118.09	100.43	96.21	91.04
hsa-miR-342-5p	MIMAT0004694	AGGGGUGCUAUCUGUGAUUGA	98.70	79.78	106.67	90.93
hsa-miR-345-5p	MIMAT0000772	GCUGACUCCUAGUCCAGGGCUC	108.92	87.21	81.18	82.90
hsa-miR-346	MIMAT0000773	UGUCUGCCCGCAUGCCUGCCUCU	115.52	110.67	102.48	116.48
hsa-miR-34a-3p	MIMAT0004557	CAAUCAGCAAGUAUACUGCCCU	70.46	98.74	78.84	66.74
hsa-miR-34a-5p	MIMAT0000255	UGGCAGUGUCUUAGCUGGUUGU	37.93	39.04	68.79	75.93
hsa-miR-34b-3p	MIMAT0004676	CAAUCACUAACUCCACUGCCAU	67.80	70.55	98.20	104.07
hsa-miR-34b-5p	MIMAT0000685	UAGGCAGUGUCAUUAGCUGAUUG	84.31	73.10	132.29	128.37
hsa-miR-34c-3p	MIMAT0004677	AAUCACUAACCACACGGCCAGG	147.12	161.18	142.49	124.44
hsa-miR-34c-5p	MIMAT0000686	AGGCAGUGUAGUUAGCUGAUUGC	33.46	39.89	80.23	91.66
hsa-miR-3605-3p	MIMAT0017982	CCUCCGUGUUACCUGUCCUCUAG	97.59	97.07	105.37	102.44
hsa-miR-3605-5p	MIMAT0017981	UGAGGAUGGAUAGCAAGGAAGCC	102.06	100.90	118.63	102.92
hsa-miR-3606-5p	MIMAT0017983	UUAGUGAAGGCUAUUUUAAUU	100.09	88.94	83.16	91.98
hsa-miR-3607-3p	MIMAT0017985	ACUGUAAACGCUUUCUGAUG	67.69	101.66	95.93	127.87
hsa-miR-3607-5p	MIMAT0017984	GCAUGUGAUGAAGCAAAUCAGU	113.58	135.74	111.96	114.43
hsa-miR-3609	MIMAT0017986	CAAAGUGAUGAGUAAUACUGGCUG	106.30	109.48	109.49	105.28
hsa-miR-3610	MIMAT0017987	GAAUCGGAAAGGAGGCGCCG	125.39	151.82	88.27	77.49
hsa-miR-3611	MIMAT0017988	UUGUGAAGAAAGAAAUUCUUA	96.95	90.18	86.00	83.11
hsa-miR-3612	MIMAT0017989	AGGAGGCAUCUUGAGAAAUGGA	48.32	67.43	97.29	91.03
hsa-miR-3613-3p	MIMAT0017991	ACAAAAAAAAAAGCCCAACCCUUC	101.42	92.48	106.73	98.81
hsa-miR-3613-5p	MIMAT0017990	UGUUGUACUUUUUUUUUUGUUC	67.79	73.85	108.15	112.09
hsa-miR-361-3p	MIMAT0004682	UCCCCCAGGUGUGAUUCUGAUUU	131.44	109.64	99.29	93.35
hsa-miR-3614-3p	MIMAT0017993	UAGCCUUCAGAUCUUGGUGUUUU	127.90	118.96	113.37	114.52
hsa-miR-3614-5p	MIMAT0017992	CCACUUGGAUCUGAAGGCUGCCC	120.40	128.01	87.39	110.00
hsa-miR-3615	MIMAT0017994	UCUCUCGGCUCCUCGCGGCUC	166.78	162.80	134.76	151.73
hsa-miR-361-5p	MIMAT0000703	UUAUCAGAAUCUCCAGGGGUAC	91.08	102.49	97.84	97.84
hsa-miR-3616-3p	MIMAT0017996	CGAGGGCAUUUCAUGAUGCAGGC	101.30	94.64	112.08	104.31
hsa-miR-3616-5p	MIMAT0017995	AUGAAGUGCACUCAUGAUAUGU	132.55	145.68	88.55	80.55
hsa-miR-3617-5p	MIMAT0017997	AAAGACAUAGUUGCAAGAUGGG	125.85	144.71	103.50	109.19
hsa-miR-3618	MIMAT0017998	UGUCUACAUUAAUGAAAAGAGC	119.16	137.73	110.67	127.90
hsa-miR-3619-5p	MIMAT0017999	UCAGCAGGCAGGCUGGUGCAGC	98.13	121.46	67.47	76.42
hsa-miR-3620-3p	MIMAT0018001	UCACCCUGCAUCCCGCACCCAG	113.57	126.62	100.18	112.83
hsa-miR-3621	MIMAT0018002	CGCGGGUCGGGGUCUGCAGG	110.44	87.21	96.87	92.24
hsa-miR-3622a-3p	MIMAT0018004	UCACCUGACCUCCCAUGCCUGU	103.98	89.93	100.96	111.13

hsa-miR-3622a-5p	MIMAT0018003	CAGGCACGGGAGCUCAGGUGAG	102.83	99.83	106.68	100.42
hsa-miR-3622b-3p	MIMAT0018006	UCACCUGAGCUCCCGUGCCUG	104.40	88.09	86.08	93.62
hsa-miR-3622b-5p	MIMAT0018005	AGGCAUGGGAGGUCAGGUGA	37.17	55.14	69.57	70.45
hsa-miR-362-3p	MIMAT0004683	AACACACCUAUUCAAGGAUUCA	98.59	86.38	84.42	90.57
hsa-miR-362-5p	MIMAT0000705	AAUCCUUGGAACCUAGGUGUGAGU	70.92	76.43	103.66	79.10
hsa-miR-363-3p	MIMAT0000707	AAUUGCACGGUAUCCAUCUGUA	129.28	84.59	104.27	130.04
hsa-miR-363-5p	MIMAT0003385	CGGGUGGAUCACGAUGCAAUUU	36.49	37.31	100.66	89.33
hsa-miR-3646	MIMAT0018065	AAAAUGAAAUGAGCCCAGCCCA	116.58	67.71	90.67	87.29
hsa-miR-3647-3p	MIMAT0018067	AGAAAAUUUUUGUGUGUCUGAUC	57.70	50.29	72.07	80.51
hsa-miR-3647-5p	MIMAT0018066	CUGAAGUGAUGAUUCACAUUCAU	137.02	129.37	120.90	117.62
hsa-miR-3648	MIMAT0018068	AGCCGCGGGGAUCGCCGAGGG	131.01	93.15	102.76	102.36
hsa-miR-3649	MIMAT0018069	AGGGACCUGAGUGUCUAAG	135.51	134.47	104.27	105.38
hsa-miR-365*	MIMAT0009199	AGGGACUUUCAGGGGCAGCUGU	83.51	75.96	108.04	95.95
hsa-miR-3650	MIMAT0018070	AGGUGUGUCUGUAGAGUCC	61.93	54.72	89.12	79.96
hsa-miR-3651	MIMAT0018071	CAUAGCCCGGUCGCUGGUACAUGA	71.53	68.86	124.78	117.51
hsa-miR-3652	MIMAT0018072	CGGCUGGAGGUGUGAGGA	88.64	81.25	135.29	139.64
hsa-miR-3653	MIMAT0018073	CUAAGAAGUUGACUGAAG	147.98	155.08	95.20	87.14
hsa-miR-3654	MIMAT0018074	GACUGGACAAGCUGAGGAA	112.21	137.75	76.50	101.08
hsa-miR-3655	MIMAT0018075	GCUUGUCGCUGCGGUGUUGCU	117.39	96.81	121.69	93.26
hsa-miR-3656	MIMAT0018076	GGCGGGUGCGGGGGUGG	127.86	102.34	120.02	113.59
hsa-miR-3657	MIMAT0018077	UGUGUCCCAUUAUUGGUGAUU	51.79	47.88	98.84	92.24
hsa-miR-3658	MIMAT0018078	UUUAAGAAAACACCAUGGAGAU	74.77	87.84	86.67	95.19
hsa-miR-3659	MIMAT0018080	UGAGUGUUGUCUACGAGGGCA	110.05	114.67	107.07	92.05
hsa-miR-365a-3p	MIMAT0000710	UAAUGCCCCUAAAAAUCCUUAU	90.34	73.74	95.73	109.13
hsa-miR-3660	MIMAT0018081	ACUGACAGGAGAGCAUUUUGA	86.57	92.08	69.76	81.69
hsa-miR-3661	MIMAT0018082	UGACCUGGGACUCGGACAGCUG	112.92	116.71	100.96	82.14
hsa-miR-3662	MIMAT0018083	GAAAAUGAUGAGUAGUGACUGAUG	87.05	93.24	92.80	96.16
hsa-miR-3663-3p	MIMAT0018085	UGAGCACCACACAGGCCGGGCGC	103.33	84.49	31.32	23.35
hsa-miR-3663-5p	MIMAT0018084	GCUGGUCUGCGUGGUGCUCGG	94.66	112.02	92.89	89.09
hsa-miR-3664-5p	MIMAT0018086	AACUCUGUCUUCACUCAUGAGU	108.69	82.95	108.92	96.92
hsa-miR-3665	MIMAT0018087	AGCAGGUGCGGGGCGGCG	3.89	19.52	98.40	85.53
hsa-miR-3666	MIMAT0018088	CAGUGCAAGUGUAGAUGCCGA	120.99	107.12	91.64	83.06
hsa-miR-3667-3p	MIMAT0018090	ACCUUCCUCUCCAUGGGUCUUU	67.88	62.37	92.46	71.03
hsa-miR-3667-5p	MIMAT0018089	AAAGACCCAUUGAGGAGAAGGU	113.73	119.79	80.66	94.20
hsa-miR-3668	MIMAT0018091	AAUGUAGAGAUUGAUCAAAAU	99.82	107.27	103.68	103.16
hsa-miR-3669	MIMAT0018092	ACGGAAUAUGUAUACGGAAUAUA	100.75	123.17	103.27	99.75
hsa-miR-3670	MIMAT0018093	AGAGCUCACAGCUGUCCUUCUCUA	107.40	105.03	89.03	93.02
hsa-miR-3671	MIMAT0018094	AUCAAAUAAGGACUAGUCUGCA	126.56	108.22	116.88	108.79
hsa-miR-3672	MIMAT0018095	AUGAGACUCAUGUAAAACAUCUU	109.40	132.11	114.06	124.81
hsa-miR-3673	MIMAT0018096	AUGGAAUGUAUAUACGGAAUA	112.48	112.18	81.18	86.62
hsa-miR-367-3p	MIMAT0000719	AAUUGCACUUUAGCAAUGGUGA	51.75	52.20	95.89	102.36

hsa-miR-3674	MIMAT0018097	AUUGUAGAACCUAAGAUUGGCC	57.75	59.08	88.17	80.59
hsa-miR-3675-3p	MIMAT0018099	CAUCUCUAAGGAACUCCCCCAA	95.99	94.26	117.61	112.83
hsa-miR-3675-5p	MIMAT0018098	UAUGGGGCUUCUGUAGAGAUUUC	124.45	136.85	105.37	109.44
hsa-miR-367-5p	MIMAT0004686	ACUGUUGCUAAUAUGCAACUCU	96.08	93.38	85.57	63.36
hsa-miR-3676-3p	MIMAT0018100	CCGUGUUUCCCCCACGCUUU	140.19	93.26	111.20	103.77
hsa-miR-3677-3p	MIMAT0018101	CUCGUGGGCUCUGGCCACGGCC	102.38	102.94	98.92	100.02
hsa-miR-3678-3p	MIMAT0018103	CUGCAGAGUUUGUACGGACCGG	71.47	87.49	87.95	81.58
hsa-miR-3678-5p	MIMAT0018102	UCCGUACAAACUCUGCUGUG	106.21	104.72	95.52	84.56
hsa-miR-3679-3p	MIMAT0018105	CUUCCCCCCAGUAAUCUUCAUC	120.91	118.50	95.52	100.75
hsa-miR-3679-5p	MIMAT0018104	UGAGGAUAUGGCAGGGAAGGGGA	69.92	77.92	94.50	71.10
hsa-miR-3680-3p	MIMAT0018107	UUUUGCAUGACCCUGGGAGUAGG	152.65	96.52	93.60	81.78
hsa-miR-3680-5p	MIMAT0018106	GACUCACUCACAGGAUUGUGCA	94.07	104.21	113.19	100.02
hsa-miR-3681-3p	MIMAT0018109	ACACAGUGCUUCAUCCACUACU	108.77	109.48	94.84	75.69
hsa-miR-3681-5p	MIMAT0018108	UAGUGGAUGAUGCACUCUGUGC	68.54	68.32	114.40	106.98
hsa-miR-3682-3p	MIMAT0018110	UGAUGAUACAGGUGGAGGUAG	93.63	121.93	96.72	118.81
hsa-miR-3683	MIMAT0018111	UGCGACAUUGGAAGUAGUAUCA	88.96	94.26	93.14	87.46
hsa-miR-3684	MIMAT0018112	UUAGACCUAGUACACGUCCUU	118.38	88.93	82.93	79.64
hsa-miR-3685	MIMAT0018113	UUUCCUACCCUACCUGAAGACU	102.06	101.66	90.42	114.52
hsa-miR-3686	MIMAT0018114	AUCUGUAAGAGAAAGUAAAUGA	80.02	85.08	80.56	93.74
hsa-miR-3687	MIMAT0018115	CCCGGACAGGCGUUCGUGCGACGU	112.73	119.13	118.45	110.54
hsa-miR-3688-3p	MIMAT0018116	UAUGGAAAGACUUUGCCACUCU	138.10	121.79	103.49	118.05
hsa-miR-3689a-3p	MIMAT0018118	CUGGGAGGUGUGAUAUCGUGGU	130.83	110.67	119.47	116.76
hsa-miR-3689a-5p	MIMAT0018117	UGUGAUAUCAUGGUUCCUGGGA	93.69	125.81	100.31	119.88
hsa-miR-3689b-3p	MIMAT0018181	CUGGGAGGUGUGAUAUUGUGGU	89.22	94.14	128.80	113.59
hsa-miR-3689b-5p	MIMAT0018180	UGUGAUAUCAUGGUUCCUGGGA	101.30	100.88	97.34	114.05
hsa-miR-3690	MIMAT0018119	ACCUGGACCCAGCGUAGACAAAG	91.75	83.49	104.92	116.48
hsa-miR-3691-5p	MIMAT0018120	AGUGGAUGAUGGAGACUCGGUAC	102.30	91.52	113.82	106.07
hsa-miR-3692-3p	MIMAT0018122	GUUCCACACUGACACUGCAGAAGU	93.57	131.08	112.22	114.07
hsa-miR-3692-5p	MIMAT0018121	CCUGCUGGUCAGGAGUGGAUACUG	137.84	140.69	106.39	111.86
hsa-miR-369-3p	MIMAT0000721	AAUAAUACAUGGUUGAUCUUU	116.11	113.15	138.23	155.59
hsa-miR-369-5p	MIMAT0001621	AGAUCGACCGUGUUAUAUUCGC	105.87	108.74	116.98	115.99
hsa-miR-370	MIMAT0000722	GCCUGCUGGGGUGGAACCUGGU	92.72	101.48	101.47	97.85
hsa-miR-3713	MIMAT0018164	GGUAUCCGUUUGGGGAUGGU	81.53	70.60	106.15	102.48
hsa-miR-3714	MIMAT0018165	GAAGGCAGCAGUGCUCCCCUGU	68.39	74.18	101.17	87.86
hsa-miR-371a-3p	MIMAT0000723	AAGUGCCGCCAUCUUUUGAGUGU	135.14	135.97	129.29	128.84
hsa-miR-371a-5p	MIMAT0004687	ACUCAAACUGUGGGGGCACU	139.61	137.14	101.10	110.15
hsa-miR-372	MIMAT0000724	AAAGUGCUGCGACAUUUGAGCGU	86.91	115.93	121.86	128.30
hsa-miR-373-3p	MIMAT0000726	GAAGUGCUUCGAUUUUGGGGUGU	139.07	135.84	138.34	158.15
hsa-miR-373-5p	MIMAT0000725	ACUCAAAAUGGGGGCGCUUUCC	151.73	161.56	111.33	92.81
hsa-miR-374a-3p	MIMAT0004688	CUUAUCAGAUUGUAUUGUAAUU	80.02	97.14	82.37	97.39
hsa-miR-374a-5p	MIMAT0000727	UUAUAAUACAACCUGAUAAGUG	87.82	96.20	114.49	84.95

hsa-miR-374b-3p	MIMAT0004956	CUUAGCAGGUUGUAUUAUCAUU	49.17	42.60	114.46	90.70
hsa-miR-374b-5p	MIMAT0004955	AUAUAAUACAACCUGCUAAGUG	64.48	70.12	116.79	117.31
hsa-miR-374c-5p	MIMAT0018443	AUAAUACAACCUGCUAAGUGCU	60.53	57.53	96.54	93.74
hsa-miR-375	MIMAT0000728	UUUGUUCGUUCGGCUCGCGUGA	115.48	101.41	97.56	93.74
hsa-miR-376a-3p	MIMAT0000729	AUCAUAGAGGAAAAUCCACGU	77.76	102.14	94.06	84.77
hsa-miR-376a-5p	MIMAT0003386	GUAGAUUCUCCUUCUAUGAGUA	104.10	94.86	84.07	96.59
hsa-miR-376b-3p	MIMAT0002172	AUCAUAGAGGAAAAUCCAUGUU	111.64	104.67	102.79	87.26
hsa-miR-376c-3p	MIMAT0000720	AACAUAGAGGAAAUUCCACGU	86.18	69.76	82.27	67.03
hsa-miR-377-3p	MIMAT0000730	AUCACACAAAGGCAACUUUUGU	98.23	106.76	93.14	109.20
hsa-miR-377-5p	MIMAT0004689	AGAGGUUGCCCUUGGUGAAUUC	57.65	56.03	76.09	69.78
hsa-miR-378a-3p	MIMAT0000732	ACUGGACUUGGAGUCAGAAGG	105.43	116.51	111.95	112.54
hsa-miR-378a-5p	MIMAT0000731	CUCCUGACUCCAGGUCCUGUGU	76.95	105.18	98.12	106.33
hsa-miR-378b	MIMAT0014999	ACUGGACUUGGAGGCAGAA	135.51	142.06	125.60	133.26
hsa-miR-378c	MIMAT0016847	ACUGGACUUGGAGUCAGAAGAGUGG	106.30	91.63	101.17	110.82
hsa-miR-379-3p	MIMAT0004690	UAUGUAACAUGGUCCACUAACU	122.50	136.81	102.48	102.16
hsa-miR-379-5p	MIMAT0000733	UGGUAGACUAUGGAACGUAGG	98.45	88.91	69.61	60.31
hsa-miR-380-3p	MIMAT0000735	UAUGUAAUAUGGUCCACAUCUU	82.26	82.76	97.85	94.58
hsa-miR-380-5p	MIMAT0000734	UGGUUGACCAUAGAACAUGCGC	65.49	58.77	134.01	130.53
hsa-miR-381-3p	MIMAT0000736	UAUACAAGGGCAAGCUCUCUGU	104.62	91.26	126.82	110.46
hsa-miR-382-5p	MIMAT0000737	GAAGUUGUUCGUGGUGGAUUCG	97.14	87.75	102.56	98.51
hsa-miR-383	MIMAT0000738	AGAUCAGAAGGUGAUUGUGGCU	49.96	61.66	125.15	132.29
hsa-miR-384	MIMAT0001075	AUUCCUAGAAAUUGUUCAUA	92.00	117.16	74.67	84.91
hsa-miR-3907	MIMAT0018179	AGGUGCUCCAGGCUGGCUCACA	49.80	49.98	81.21	88.26
hsa-miR-3908	MIMAT0018182	GAGCAAUGUAGGUAGACUGUUU	71.96	89.05	96.39	73.13
hsa-miR-3909	MIMAT0018183	UGUCCUCUAGGGCCUGCAGUCU	126.95	142.06	80.80	85.00
hsa-miR-3910	MIMAT0018184	AAAGGCAUAAAACCAAGACA	81.97	61.29	81.32	99.55
hsa-miR-3911	MIMAT0018185	UGUGUGGAUCCUGGAGGAGGCA	73.21	57.48	97.33	106.98
hsa-miR-3912	MIMAT0018186	UAACGCAUAAUAUGGACAUGU	96.86	118.84	86.55	116.34
hsa-miR-3913-5p	MIMAT0018187	UUUGGGACUGAUCUUGAUGUCU	125.31	115.46	108.72	103.35
hsa-miR-3914	MIMAT0018188	AAGGAACCAGAAAAUGAGAAGU	119.95	112.63	105.04	120.32
hsa-miR-3915	MIMAT0018189	UUGAGGAAAAGAUGGUCUUAUU	97.89	101.57	93.08	90.55
hsa-miR-3916	MIMAT0018190	AAGAGGAAGAAAUGGCUGGUUCUCAG	74.50	90.71	106.23	122.56
hsa-miR-3917	MIMAT0018191	GCUCGGACUGAGCAGGUGGG	107.79	93.61	101.73	123.09
hsa-miR-3918	MIMAT0018192	ACAGGGCCGCAGAUGGAGACU	72.78	87.36	91.69	85.87
hsa-miR-3919	MIMAT0018193	GCAGAGAACAAAGGACUCAGU	78.10	71.66	86.92	107.97
hsa-miR-3920	MIMAT0018195	ACUGAUUAUCUUAACUCUCUGA	123.81	122.45	103.97	83.26
hsa-miR-3921	MIMAT0018196	UCUCUGAGUACCAUAUGCCUUGU	82.51	103.53	98.95	105.67
hsa-miR-3922-3p	MIMAT0018197	UCUGGCCUUGACUUGACUCUUU	75.55	116.04	105.33	80.17
hsa-miR-3923	MIMAT0018198	AACUAGUAAUGUUGGAUUAGGG	86.15	79.32	98.84	102.15
hsa-miR-3924	MIMAT0018199	AUAUGUAUAUGUGACUGCUACU	107.57	88.52	92.16	84.36
hsa-miR-3925-5p	MIMAT0018200	AAGAGAACUGAAAGUGGAGCCU	87.28	90.12	117.40	99.17

hsa-miR-3926	MIMAT0018201	UGGCCAAAAAGCAGGCAGAGA	116.71	119.00	97.29	112.80
hsa-miR-3927-3p	MIMAT0018202	CAGGUAGAUAUUUGAUAGGCAU	96.95	90.95	82.26	92.53
hsa-miR-3928	MIMAT0018205	GGAGGAACCUUGGAGCUUCGGC	83.26	101.94	101.73	94.20
hsa-miR-3929	MIMAT0018206	GAGGCUGAUGUGAGUAGACCACU	96.57	93.26	90.93	103.23
hsa-miR-3934-5p	MIMAT0018349	UCAGGUGUGGAAACUGAGGCAG	95.89	99.17	109.49	105.67
hsa-miR-3935	MIMAT0018350	UGUAGAUACGAGCACCAGCCAC	67.87	59.83	77.45	69.89
hsa-miR-3936	MIMAT0018351	UAAGGGGUGUAUGGCAGAUGCA	83.79	96.86	84.61	106.26
hsa-miR-3937	MIMAT0018352	ACAGGCGGCUGUAGCAAUGGGGG	89.12	117.65	103.25	89.91
hsa-miR-3938	MIMAT0018353	AAUUCCCUUGUAGAUAACCCGG	94.07	116.71	90.08	90.60
hsa-miR-3939	MIMAT0018355	UACGCGCAGACCACAGGAUGUC	91.43	83.30	118.36	113.19
hsa-miR-3940-3p	MIMAT0018356	CAGCCCGGAUCCCAGCCCACUU	131.99	111.31	103.03	123.37
hsa-miR-3941	MIMAT0018357	UUACACACAACUGAGGAUCAUA	79.70	87.38	81.24	81.18
hsa-miR-3942-5p	MIMAT0018358	AAGCAAUACUGUUACCUGAAAU	86.68	94.90	93.44	87.25
hsa-miR-3943	MIMAT0018359	UAGCCCCCAGGCUUCACUUGGCG	115.78	91.67	114.53	113.65
hsa-miR-3944-3p	MIMAT0018360	UUCGGGCUGGCCUGCUGCUCCGG	128.50	147.49	83.47	72.66
hsa-miR-3945	MIMAT0018361	AGGGCAUAGGAGAGGGUUGAUAU	105.11	91.67	99.33	117.54
hsa-miR-409-3p	MIMAT0001639	GAAUGUUGCUCGGUGAACCCCU	89.12	94.64	68.91	79.01
hsa-miR-409-5p	MIMAT0001638	AGGUUACCCGAGCAACUUUGCAU	101.74	98.34	91.44	94.22
hsa-miR-410	MIMAT0002171	AAUAUAACACAGAUGGCCUGU	86.25	78.13	120.83	119.59
hsa-miR-411-3p	MIMAT0004813	UAUGUAACACGGUCCACUAACC	113.07	110.81	118.49	112.90
hsa-miR-411-5p	MIMAT0003329	UAGUAGACCGUAUAGCGUACG	92.18	96.39	99.51	96.18
hsa-miR-412	MIMAT0002170	ACUUCACCUGGUCCACUAGCCGU	117.10	74.16	69.14	75.67
hsa-miR-421	MIMAT0003339	AUCAACAGACAUUAAUUGGGCGC	104.26	84.09	86.65	67.60
hsa-miR-422a	MIMAT0001339	ACUGGACUUAGGGUCAGAAGGC	132.40	119.29	143.20	146.67
hsa-miR-423-3p	MIMAT0001340	AGCUCGGUCUGAGGCCCCUCAGU	104.04	95.64	95.89	82.26
hsa-miR-423-5p	MIMAT0004748	UGAGGGGCAGAGAGCGAGACUUU	89.15	82.78	126.58	115.28
hsa-miR-424-3p	MIMAT0004749	CAAAACGUGAGGCGCUGCUAU	74.39	67.76	121.21	120.82
hsa-miR-424-5p	MIMAT0001341	CAGCAGCAAUUCAUGUUUUGAA	81.40	85.91	86.20	83.08
hsa-miR-4251	MIMAT0016883	CCUGAGAAAAGGGCCAA	121.17	108.29	82.88	100.53
hsa-miR-4252	MIMAT0016886	GGCCACUGAGUCAGCACCA	89.70	83.49	101.60	112.83
hsa-miR-4253	MIMAT0016882	AGGGCAUGUCCAGGGGGU	63.86	50.97	96.80	80.17
hsa-miR-425-3p	MIMAT0001343	AUCGGGAAUGUCGUGUCCGCCC	85.25	77.76	97.34	77.81
hsa-miR-4254	MIMAT0016884	GCCUGGAGCUACUCCACCAUCUC	88.79	68.32	81.87	81.24
hsa-miR-4255	MIMAT0016885	CAGUGUUCAGAGAUGGA	81.51	84.88	110.13	101.49
hsa-miR-425-5p	MIMAT0003393	AAUGACACGAUCACUCCCGUUGA	50.61	74.16	92.31	101.25
hsa-miR-4256	MIMAT0016877	AUCUGACCUGAUGAAGGU	67.19	73.75	90.24	80.42
hsa-miR-4257	MIMAT0016878	CCAGAGGUGGGGACUGAG	114.60	102.85	103.96	87.77
hsa-miR-4258	MIMAT0016879	CCCCGCCACCGCCUUGG	92.18	86.47	87.87	102.51
hsa-miR-4259	MIMAT0016880	CAGUUGGGUCUAGGGGUCAGGA	88.45	67.99	122.59	119.47
hsa-miR-4260	MIMAT0016881	CUUGGGGCAUGGAGUCCCA	115.22	83.30	100.62	100.53
hsa-miR-4261	MIMAT0016890	AGGAAACAGGGACCCA	132.67	140.53	147.55	152.13

hsa-miR-4262	MIMAT0016894	GACAUUCAGACUACCUG	102.38	119.01	90.08	92.77
hsa-miR-4263	MIMAT0016898	AUUCUAAGUGCCUUGGCC	100.80	119.86	94.67	89.41
hsa-miR-4264	MIMAT0016899	ACUCAGUCAUGGUCAUU	78.10	80.70	129.08	103.92
hsa-miR-4265	MIMAT0016891	CUGUGGGCUCAGCUCUGGG	51.79	67.17	116.49	117.54
hsa-miR-4266	MIMAT0016892	CUAGGAGGCCUUGGCC	86.83	71.63	108.65	96.14
hsa-miR-4267	MIMAT0016893	UCCAGCUCGGUGGCAC	93.69	89.44	69.41	76.29
hsa-miR-4268	MIMAT0016896	GGCUCCUCCUCUCAGGAUGUG	109.22	95.01	82.76	85.15
hsa-miR-4269	MIMAT0016897	GCAGGCACAGACAGCCCUGGC	99.78	102.06	84.61	105.09
hsa-miR-4270	MIMAT0016900	UCAGGGAGUCAGGGGAGGGC	89.70	92.53	115.34	103.92
hsa-miR-4271	MIMAT0016901	GGGGGAAGAAAAGGUGGGG	84.61	63.63	70.70	65.87
hsa-miR-4272	MIMAT0016902	CAUUCAACUAGUGAUUGU	108.92	68.53	94.99	75.29
hsa-miR-4273	MIMAT0016903	GUGUUCUCUGAUGGACAG	100.35	95.60	113.92	106.07
hsa-miR-4274	MIMAT0016906	CAGCAGUCCCUCCCCCUG	103.59	104.29	80.69	70.84
hsa-miR-4275	MIMAT0016905	CCAAUUACCACUUCUUU	94.31	100.27	97.76	108.31
hsa-miR-4276	MIMAT0016904	CUCAGUGACUCAUGUGC	63.86	66.15	79.73	75.88
hsa-miR-4277	MIMAT0016908	GCAGUUCUGAGCACAGUACAC	87.95	84.97	88.38	98.17
hsa-miR-4278	MIMAT0016910	CUAGGGGGUUUGCCCUUG	80.76	68.86	89.34	94.96
hsa-miR-4279	MIMAT0016909	CUCUCCUCCCGGCUUC	145.42	133.13	129.58	146.11
hsa-miR-4280	MIMAT0016911	GAGUGUAGUUCUGAGCAGAGC	111.97	119.13	102.27	114.43
hsa-miR-4281	MIMAT0016907	GGGUCCCGGGGAGGGGGG	109.68	108.00	115.02	107.81
hsa-miR-4282	MIMAT0016912	UAAAAUUUGCAUCCAGGA	100.64	100.32	78.00	96.12
hsa-miR-4283	MIMAT0016914	UGGGGCUCAGCGAGUUU	109.27	113.05	90.08	106.86
hsa-miR-4284	MIMAT0016915	GGGCUCACAUCACCCCAU	116.91	115.06	111.99	132.55
hsa-miR-4285	MIMAT0016913	GCGGCGAGUCCGACUCAU	104.07	100.75	117.25	113.19
hsa-miR-4286	MIMAT0016916	ACCCCACUCCUGGUACC	145.18	148.60	124.41	100.02
hsa-miR-4287	MIMAT0016917	UCUCCCUUGAGGGCACUUU	93.46	104.11	99.47	101.62
hsa-miR-4288	MIMAT0016918	UUGUCUGCUGAGUUUCC	72.19	80.86	73.85	82.87
hsa-miR-4289	MIMAT0016920	GCAUUGUGCAGGGCUAUCA	116.75	111.36	95.52	109.93
hsa-miR-429	MIMAT0001536	UAAUACUGUCUGGUAAAACCGU	55.60	50.10	77.25	54.10
hsa-miR-4290	MIMAT0016921	UGCCCUCCUUUCUUCCCUC	66.51	91.83	103.97	117.29
hsa-miR-4291	MIMAT0016922	UUCAGCAGGAACAGCU	78.45	92.41	84.12	106.26
hsa-miR-4292	MIMAT0016919	CCCCUGGGCCGGCCUUGG	130.76	155.15	110.90	126.01
hsa-miR-4293	MIMAT0016848	CAGCCUGACAGGAACAG	67.87	68.18	69.39	75.56
hsa-miR-4294	MIMAT0016849	GGGAGUCUACAGCAGGG	125.63	113.84	118.91	87.86
hsa-miR-4295	MIMAT0016844	CAGUGCAAUGUUUUCCUU	137.45	114.79	123.87	97.44
hsa-miR-4296	MIMAT0016845	AUGUGGGCUCAGGCUCA	75.55	66.15	105.33	109.67
hsa-miR-4297	MIMAT0016846	UGCCUUCCUGUCUGUG	121.08	86.27	108.24	137.95
hsa-miR-4298	MIMAT0016852	CUGGGACAGGAGGAGGAGGCAG	126.37	110.67	131.11	123.09
hsa-miR-4299	MIMAT0016851	GCUGGUGACAUGAGAGGC	121.17	129.71	93.41	112.01
hsa-miR-4300	MIMAT0016853	UGGGAGCUGGACUACUUC	110.02	102.74	102.83	89.05
hsa-miR-4301	MIMAT0016850	UCCCACUACUUCACUUGUGA	104.07	115.83	112.81	98.95

hsa-miR-4302	MIMAT0016855	CCAGUGUGGCUCAGCGAG	105.21	123.41	66.19	84.93
hsa-miR-4303	MIMAT0016856	UUCUGAGCUGAGGACAG	80.28	89.25	31.88	36.02
hsa-miR-4304	MIMAT0016854	CCGGCAUGUCCAGGGCA	104.02	94.62	105.87	115.26
hsa-miR-4305	MIMAT0016857	CCUAGACACCUCCAGUUC	69.24	86.27	99.71	82.85
hsa-miR-4306	MIMAT0016858	UGGAGAGAAAGGCAGUA	81.00	62.90	92.53	100.55
hsa-miR-4307	MIMAT0016860	AAUGUUUUUUCCUGUUUCC	99.34	79.64	99.25	90.75
hsa-miR-4308	MIMAT0016861	UCCCUGGAGUUUCUUCUU	88.96	94.77	111.49	90.36
hsa-miR-4309	MIMAT0016859	CUGGAGUCUAGGAUUCCA	86.23	83.70	101.73	94.59
hsa-miR-4310	MIMAT0016862	GCAGCAUUCAUGUCCC	107.48	82.42	111.73	99.48
hsa-miR-4311	MIMAT0016863	GAAAGAGAGCUGAGUGUG	105.13	105.57	111.09	111.17
hsa-miR-4312	MIMAT0016864	GGCCUUGUUCCUGUCCCCA	66.28	62.63	104.02	92.29
hsa-miR-4313	MIMAT0016865	AGCCCCCUGGCCCCAAACCC	122.86	124.31	115.82	109.18
hsa-miR-431-3p	MIMAT0004757	CAGGUCGUCUUGCAGGGCUUCU	79.00	65.89	112.33	117.31
hsa-miR-4314	MIMAT0016868	CUCUGGGAAAUGGGACAG	102.07	105.03	74.31	75.51
hsa-miR-4315	MIMAT0016866	CCGCUUUCUGAGCUGGAC	93.75	95.28	117.61	85.53
hsa-miR-431-5p	MIMAT0001625	UGUCUUGCAGGCCGUCAUGCA	74.01	53.57	98.76	84.88
hsa-miR-4316	MIMAT0016867	GGUGAGGCUAGCUGGUG	29.73	76.16	93.96	81.14
hsa-miR-4317	MIMAT0016872	ACAUUGCCAGGGAGUUU	95.23	112.38	100.09	95.03
hsa-miR-4318	MIMAT0016869	CACUGUGGGUACAUGCU	114.25	120.62	84.61	93.80
hsa-miR-4319	MIMAT0016870	UCCCUGAGCAAAGCCAC	89.02	84.88	96.87	97.03
hsa-miR-4320	MIMAT0016871	GGGAUUCUGUAGCUUCCU	60.75	79.16	82.40	84.46
hsa-miR-4321	MIMAT0016874	UUAGCGGUGGACCGCCCUGCG	116.14	112.44	103.96	132.55
hsa-miR-4322	MIMAT0016873	CUGUGGGCUCAGCGCGUGGGG	103.84	95.01	109.07	99.86
hsa-miR-4323	MIMAT0016875	CAGCCCCACAGCCUCAGA	93.46	80.25	120.27	118.78
hsa-miR-432-3p	MIMAT0002815	CUGGAUGGCUCCUCCAUGUCU	119.74	129.95	127.80	98.41
hsa-miR-4324	MIMAT0016876	CCCUGAGACCCUAACCUUAA	119.99	121.16	102.49	100.51
hsa-miR-4325	MIMAT0016887	UUGCACUUGUCUCAGUGA	81.50	93.15	96.38	112.87
hsa-miR-432-5p	MIMAT0002814	UCUUGGAGUAGGUCAUUGGGUGG	137.46	138.43	101.47	88.82
hsa-miR-4326	MIMAT0016888	UGUUCCUCUGUCUCCCAGAC	42.06	47.72	92.00	84.46
hsa-miR-4327	MIMAT0016889	GGCUUGCAUGGGGGACUGG	130.76	110.70	94.46	79.92
hsa-miR-4328	MIMAT0016926	CCAGUUUUCCCAGGAUU	95.66	98.78	72.21	87.93
hsa-miR-4329	MIMAT0016923	CCUGAGACCCUAGUUCCAC	127.11	119.40	78.44	87.47
hsa-miR-433	MIMAT0001627	AUCAUGAUGGGCUCCUCGGUGU	113.56	98.09	89.06	98.09
hsa-miR-4330	MIMAT0016924	CCUCAGAUCAGAGCCUUGC	81.77	60.69	91.75	103.70
hsa-miR-448	MIMAT0001532	UUGCAUAUGUAGGAUGUCCCAU	99.73	111.45	114.07	110.13
hsa-miR-449a	MIMAT0001541	UGGCAGUGUAUUGUUAGCUGGU	21.82	27.24	45.59	62.93
hsa-miR-449b-3p	MIMAT0009203	CAGCCACAACUACCCUGCCACU	103.77	94.79	131.84	107.89
hsa-miR-449b-5p	MIMAT0003327	AGGCAGUGUAUUGUUAGCUGGC	36.17	26.71	65.57	60.88
hsa-miR-449c-3p	MIMAT0013771	UUGCUAGUUGCACUCCUCUCUGU	145.70	142.40	101.73	105.67
hsa-miR-449c-5p	MIMAT0010251	UAGGCAGUGUAUUGCUAGCGGCUGU	91.29	66.05	94.37	84.42
hsa-miR-450a-5p	MIMAT0001545	UUUUGCGAUGUGUUCCUAAUAU	169.53	177.24	156.37	178.96

hsa-miR-450b-3p	MIMAT0004910	UUGGGAUCAUUUUGCAUCCAUA	95.41	92.40	98.49	107.28
hsa-miR-450b-5p	MIMAT0004909	UUUUGCAAUAUGUUCCUGAAUA	36.30	34.18	86.13	94.62
hsa-miR-451a	MIMAT0001631	AAACCGUUACCAUUACUGAGUU	80.71	89.15	96.72	133.15
hsa-miR-452-3p	MIMAT0001636	CUCAUCUGCAAAGAAGUAAGUG	86.91	92.39	101.40	84.50
hsa-miR-452-5p	MIMAT0001635	AACUGUUUGCAGAGGAAACUGA	136.07	110.20	85.24	87.48
hsa-miR-453	MIMAT0001630	AGGUUGUCCGUGGUGAGUUCGCA	102.07	99.83	116.00	128.83
hsa-miR-454-3p	MIMAT0003885	UAGUGCAAUAUUGCUUAUAGGGU	133.80	134.07	102.83	106.86
hsa-miR-454-5p	MIMAT0003884	ACCCUAUCAAUAUUGUCUCUGC	129.91	129.36	143.18	158.56
hsa-miR-455-3p	MIMAT0004784	GCAGUCCAUGGGCAUAUACAC	129.22	126.39	86.78	115.88
hsa-miR-455-5p	MIMAT0003150	UAUGUGCCUUUGGACUACAUCG	94.45	75.34	126.30	112.87
hsa-miR-466	MIMAT0015002	AUACACAUACACGCAACACACAU	102.26	111.18	87.04	88.59
hsa-miR-483-3p	MIMAT0002173	UCACUCCUCUCCUCCCGUCUU	128.29	126.11	96.63	95.38
hsa-miR-483-5p	MIMAT0004761	AAGACGGGAGGAAAGAAGGGAG	87.08	90.20	119.87	107.38
hsa-miR-484	MIMAT0002174	UCAGGCUCAGUCCCCUCCCGAU	150.67	155.24	100.29	108.68
hsa-miR-485-3p	MIMAT0002176	GUCAUACACGGCUCUCCUCUCU	117.43	134.25	94.49	131.44
hsa-miR-485-5p	MIMAT0002175	AGAGGCUGGCCGUGAUGAAUUC	68.03	76.05	111.06	125.66
hsa-miR-486-3p	MIMAT0004762	CGGGGCAGCUCAGUACAGGAU	96.58	87.82	111.05	115.17
hsa-miR-486-5p	MIMAT0002177	UCCUGUACUGAGCUGCCCCGAG	157.32	116.04	109.60	104.30
hsa-miR-487a	MIMAT0002178	AAUCAUACAGGGACAUCCAGUU	101.88	117.04	95.51	92.10
hsa-miR-487b	MIMAT0003180	AAUCGUACAGGGUCAUCCACUU	137.84	139.42	114.89	120.07
hsa-miR-488-3p	MIMAT0004763	UUGAAAGGCUAUUUCUUGGUC	109.93	95.32	102.79	110.63
hsa-miR-488-5p	MIMAT0002804	CCCAGAUAAUGGCACUCUCAA	86.25	100.83	108.17	88.82
hsa-miR-489	MIMAT0002805	GUGACAUCACAUAUACGGCAGC	115.96	87.66	107.82	93.01
hsa-miR-490-3p	MIMAT0002806	CAACCUGGAGGACUCCAUGCUG	120.76	107.21	106.88	117.51
hsa-miR-490-5p	MIMAT0004764	CCAUGGAUCUCCAGGUGGGU	57.96	61.77	101.37	97.88
hsa-miR-491-3p	MIMAT0004765	CUUAUGCAAGAUUCCCUUCUAC	159.71	132.00	88.08	101.90
hsa-miR-491-5p	MIMAT0002807	AGUGGGGAACCCUUCCAUGAGG	117.07	88.40	102.32	99.78
hsa-miR-492	MIMAT0002812	AGGACCUGCGGGACAAGAUUCUU	92.16	105.77	95.89	109.37
hsa-miR-493-3p	MIMAT0003161	UGAAGGUCUACUGUGUGCCAGG	153.63	201.98	117.11	123.28
hsa-miR-493-5p	MIMAT0002813	UUGUACAUGGUAGGCUUUCAUU	101.74	92.99	109.45	100.99
hsa-miR-494	MIMAT0002816	UGAAACAUACACGGGAAACCUC	82.37	70.77	137.80	136.08
hsa-miR-495-3p	MIMAT0002817	AAACAAACAUGGUGCACUUCUU	92.70	86.00	102.44	81.54
hsa-miR-496	MIMAT0002818	UGAGUAUUACAUGGCCAAUCUC	105.48	110.99	120.69	107.03
hsa-miR-497-3p	MIMAT0004768	CAAACCACACUGUGGUGUUAGA	91.66	85.42	112.75	72.08
hsa-miR-497-5p	MIMAT0002820	CAGCAGCACACUGUGGUUUGU	63.98	64.95	70.39	75.90
hsa-miR-498	MIMAT0002824	UUUCAAGCCAGGGGGCGUUUUUC	62.30	70.60	96.28	109.01
hsa-miR-499a-3p	MIMAT0004772	AACAUCACAGCAAGUCUGUGCU	89.25	69.32	117.05	97.13
hsa-miR-499a-5p	MIMAT0002870	UUAAGACUUGCAGUGAUGUUU	119.13	111.85	85.46	88.41
hsa-miR-500a-3p	MIMAT0002871	AUGCACCUGGGCAAGGAUUCUG	95.38	101.98	98.84	119.47
hsa-miR-500a-5p	MIMAT0004773	UAAUCCUUGCUACCUGGGUGAGA	177.47	179.25	120.91	117.15
hsa-miR-500b	MIMAT0016925	AAUCCUUGCUACCUGGGU	90.69	104.32	97.29	99.74

hsa-miR-501-3p	MIMAT0004774	AAUGCACCCGGGCAAGGAUUCU	160.99	168.08	89.47	97.61
hsa-miR-501-5p	MIMAT0002872	AAUCCUUUGUCCCUGGGUGAGA	113.18	101.18	86.50	92.84
hsa-miR-502-3p	MIMAT0004775	AAUGCACCUGGGCAAGGAUUCA	85.83	87.74	120.13	121.16
hsa-miR-502-5p	MIMAT0002873	AUCCUUGCUAUCUGGGUGCUA	82.88	82.25	77.36	80.68
hsa-miR-503-5p	MIMAT0002874	UAGCAGCGGGAACAGUUCUGCAG	57.78	71.44	67.78	77.43
hsa-miR-504	MIMAT0002875	AGACCCUGGUCUGCACUCUAUC	83.53	86.10	105.04	95.19
hsa-miR-505-3p	MIMAT0002876	CGUCAACACUUGCUGGUUUCCU	108.66	99.59	115.06	121.02
hsa-miR-505-5p	MIMAT0004776	GGGAGCCAGGAAGUAUUGAUGU	89.48	77.48	111.52	103.18
hsa-miR-506-3p	MIMAT0002878	UAAGGCACCCUUCUGAGUAGA	133.97	129.18	143.52	148.45
hsa-miR-507	MIMAT0002879	UUUUGCACCUUUUGGAGUGAA	58.04	68.11	78.87	76.11
hsa-miR-508-3p	MIMAT0002880	UGAUUGUAGCCUUUUGGAGUAGA	102.10	144.70	109.86	74.53
hsa-miR-508-5p	MIMAT0004778	UACUCCAGAGGGCGUCACUCAUG	32.03	46.16	75.53	59.94
hsa-miR-509-3-5p	MIMAT0004975	UACUGCAGACGUGGCAAUCAUG	71.69	63.10	134.57	107.57
hsa-miR-509-3p	MIMAT0002881	UGAUUGGUACGUCUGUGGGUAG	111.32	105.74	114.21	114.76
hsa-miR-509-5p	MIMAT0004779	UACUGCAGACAGUGGCAAUCA	35.04	32.29	129.14	130.42
hsa-miR-510	MIMAT0002882	UACUCAGGAGAGUGGCAAUCAC	36.15	43.58	87.51	115.88
hsa-miR-511	MIMAT0002808	GUGUCUUUUGCUCUGCAGUCA	139.11	125.92	116.77	97.31
hsa-miR-512-3p	MIMAT0002823	AAGUGCUGUCAUAGCUGAGGUC	156.83	167.92	95.63	99.30
hsa-miR-512-5p	MIMAT0002822	CACUCAGCCUUGAGGGCACUUUC	116.50	85.27	76.32	59.02
hsa-miR-513a-3p	MIMAT0004777	UAAAUUUCACCUUUCUGAGAAGG	121.07	100.43	107.66	69.81
hsa-miR-513a-5p	MIMAT0002877	UUCACAGGGAGGUGUCAU	73.99	79.16	85.07	83.39
hsa-miR-513b	MIMAT0005788	UUCACAAGGAGGUGUCAUUUAU	82.38	104.36	75.16	98.36
hsa-miR-513c-5p	MIMAT0005789	UUCUCAAGGAGGUGUCGUUUAU	118.20	108.17	129.61	129.02
hsa-miR-514a-3p	MIMAT0002883	AUUGACACUUCUGUGAGUAGA	71.42	66.15	87.06	76.14
hsa-miR-514b-3p	MIMAT0015088	AUUGACACCUCUGUGAGUGGA	89.12	99.83	82.65	87.18
hsa-miR-514b-5p	MIMAT0015087	UUCUCAAGAGGGAGGCAAUCAU	136.03	112.65	109.49	110.03
hsa-miR-515-3p	MIMAT0002827	GAGUGCCUUCUUUUGGAGCGUU	174.45	173.51	119.73	118.78
hsa-miR-515-5p	MIMAT0002826	UUCUCCAAAAGAAAGCACUUUCUG	120.57	88.57	70.82	104.45
hsa-miR-516a-3p	MIMAT0002860	UGCUUCCUUUCAGAGGGU	98.63	99.52	94.91	113.53
hsa-miR-516a-5p	MIMAT0004770	UUCUCGAGGAAAGAAGCACUUUC	121.17	102.34	112.26	97.36
hsa-miR-516b-5p	MIMAT0002859	AUCUGGAGGUAAGAAGCACUUU	118.46	94.27	75.95	67.73
hsa-miR-517-5p	MIMAT0002851	CCUCUAGAUGGAAGCACUGUCU	122.36	98.88	103.33	107.69
hsa-miR-517a-3p	MIMAT0002852	AUCGUGCAUCCCUUUAGAGUGU	76.44	99.01	61.60	42.38
hsa-miR-517b	MIMAT0002857	UCGUGCAUCCCUUUAGAGUGUU	150.89	144.26	107.34	122.39
hsa-miR-517c-3p	MIMAT0002866	AUCGUGCAUCCUUUUAGAGUGU	87.25	85.64	128.09	105.51
hsa-miR-518a-3p	MIMAT0002863	GAAAGCGCUUCCCUUUGCUGGA	99.79	85.50	104.04	90.63
hsa-miR-518a-5p	MIMAT0005457	CUGCAAAGGGAAGCCCUUUC	22.37	17.67	59.23	41.25
hsa-miR-518b	MIMAT0002844	CAAAGCGCUCCCCUUUAGAGGU	109.27	89.65	85.65	92.22
hsa-miR-518c-3p	MIMAT0002848	CAAAGCGCUUCUCUUUAGAGUGU	101.10	133.28	102.83	108.05
hsa-miR-518c-5p	MIMAT0002847	UCUCUGGAGGGAAGCACUUUCUG	107.03	91.11	95.78	97.19
hsa-miR-518d-3p	MIMAT0002864	CAAAGCGCUUCCCUUUGGAGC	84.89	113.87	98.93	67.30

hsa-miR-518d-5p	MIMAT0005456	CUCUAGAGGGAAGCACUUUCUG	144.45	145.19	120.18	127.57
hsa-miR-518e-3p	MIMAT0002861	AAAGCGCUUCCCUUCAGAGUG	116.49	98.12	118.83	117.92
hsa-miR-518e-5p	MIMAT0005450	CUCUAGAGGGAAGCGCUUUCUG	92.67	76.46	82.22	112.60
hsa-miR-518f-3p	MIMAT0002842	GAAAGCGCUUCUCUUUAGAGG	100.65	89.88	80.88	113.61
hsa-miR-518f-5p	MIMAT0002841	CUCUAGAGGGAAGCACUUUCUC	106.81	113.45	85.45	87.29
hsa-miR-519a-3p	MIMAT0002869	AAAGUGCAUCCUUUUAGAGUGU	125.97	131.43	137.97	161.83
hsa-miR-519b-3p	MIMAT0002837	AAAGUGCAUCCUUUUAGAGGUU	134.15	120.33	115.46	124.00
hsa-miR-519c-3p	MIMAT0002832	AAAGUGCAUCUUUUUAGAGGAU	113.20	121.58	114.13	116.31
hsa-miR-519d	MIMAT0002853	CAAAGUGCCUCCCUUUAGAGUG	80.76	86.29	124.42	114.57
hsa-miR-519e-3p	MIMAT0002829	AAGUGCCUCCUUUUAGAGUGUU	99.19	122.33	82.26	76.10
hsa-miR-519e-5p	MIMAT0002828	UUCUCCAAAAGGGAGCACUUUC	108.53	106.16	68.25	73.52
hsa-miR-520a-3p	MIMAT0002834	AAAGUGCUUCCCUUUGGACUGU	137.73	136.43	138.20	151.71
hsa-miR-520a-5p	MIMAT0002833	CUCCAGAGGGAAGUACUUUCU	37.98	45.06	83.22	78.56
hsa-miR-520b	MIMAT0002843	AAAGUGCUUCCUUUUAGAGGG	97.08	118.93	112.27	134.03
hsa-miR-520c-3p	MIMAT0002846	AAAGUGCUUCCUUUUAGAGGGU	106.85	119.01	122.03	103.16
hsa-miR-520d-3p	MIMAT0002856	AAAGUGCUUCUCUUUGGUGGGU	166.82	203.57	132.14	136.45
hsa-miR-520d-5p	MIMAT0002855	CUACAAAGGGAAGCCCUUUC	162.63	147.31	104.33	87.81
hsa-miR-520e	MIMAT0002825	AAAGUGCUUCCUUUUUGAGGG	100.08	101.57	94.08	123.82
hsa-miR-520f	MIMAT0002830	AAGUGCUUCCUUUUAGAGGGUU	165.34	152.49	164.35	140.85
hsa-miR-520g	MIMAT0002858	ACAAAGUGCUUCCCUUUAGAGUGU	125.60	107.31	114.13	118.77
hsa-miR-520h	MIMAT0002867	ACAAAGUGCUUCCCUUUAGAGU	78.13	84.50	89.97	88.43
hsa-miR-521	MIMAT0002854	AACGCACUUCCCUUUAGAGUGU	101.53	90.65	78.01	102.80
hsa-miR-522-3p	MIMAT0002868	AAAAUGGUUCCCUUUAGAGUGU	46.90	31.45	117.11	110.56
hsa-miR-523-3p	MIMAT0002840	GAACGCGCUUCCCUAUAGAGGGU	101.58	100.63	115.05	107.89
hsa-miR-524-3p	MIMAT0002850	GAAGGCGCUUCCCUUUGGAGU	62.30	63.63	81.67	92.34
hsa-miR-524-5p	MIMAT0002849	CUACAAAGGGAAGCACUUUCUC	127.02	106.37	119.06	109.29
hsa-miR-525-3p	MIMAT0002839	GAAGGCGCUUCCCUUUAGAGCG	93.83	117.46	116.10	141.40
hsa-miR-525-5p	MIMAT0002838	CUCCAGAGGGAUGCACUUUCU	111.66	129.42	107.17	95.06
hsa-miR-526b-3p	MIMAT0002836	GAAAGUGCUUCCUUUUAGAGGC	176.36	184.28	86.13	110.03
hsa-miR-526b-5p	MIMAT0002835	CUCUUGAGGGAAGCACUUUCUGU	63.86	92.18	109.07	100.01
hsa-miR-532-3p	MIMAT0004780	CCUCCCACACCCAAGGCUUGCA	112.93	92.18	77.60	75.35
hsa-miR-532-5p	MIMAT0002888	CAUGCCUUGAGUGUAGGACCGU	137.87	163.67	120.42	117.54
hsa-miR-539-5p	MIMAT0003163	GGAGAAAUUAUCCUUGGUGUGU	140.97	167.01	109.60	117.17
hsa-miR-541-3p	MIMAT0004920	UGGUGGGCACAGAAUCUGGACU	64.74	57.94	120.81	124.53
hsa-miR-541-5p	MIMAT0004919	AAAGGAUUCUGCUGUCGGUCCCACU	116.50	97.56	111.06	108.53
hsa-miR-542-3p	MIMAT0003389	UGUGACAGAUUGAUAACUGAAA	58.49	67.39	81.94	95.48
hsa-miR-542-5p	MIMAT0003340	UCGGGGAUCAUCAUGUCACGAGA	94.13	105.24	103.09	106.62
hsa-miR-543	MIMAT0004954	AAACAUUCGCGGUGCACUUCUU	88.46	128.52	103.39	101.72
hsa-miR-544a	MIMAT0003164	AUUCUGCAUUUUUAGCAAGUUC	51.10	50.43	107.64	87.16
hsa-miR-544b	MIMAT0015004	ACCUGAGGUUGUGCAUUUCUAA	92.43	62.61	92.60	80.02
hsa-miR-545-3p	MIMAT0003165	UCAGCAAACAUUUAUUGUGUGC	141.67	113.87	97.44	91.55

hsa-miR-545-5p	MIMAT0004785	UCAGUAAAUGUUUAUUAGAUGA	87.58	97.14	68.67	75.43
hsa-miR-548a-3p	MIMAT0003251	CAAAACUGGCAAUUACUUUUGC	118.09	97.04	134.19	110.36
hsa-miR-548a-5p	MIMAT0004803	AAAAGUAAUUGCGAGUUUUACC	101.58	103.71	108.79	104.72
hsa-miR-548aa	MIMAT0018447	AAAAACCACAAUUACUUUUGCACCA	63.78	75.14	102.08	104.73
hsa-miR-548b-3p	MIMAT0003254	CAAGAACCUCAGUUGCUUUUGU	105.89	84.06	92.80	107.03
hsa-miR-548b-5p	MIMAT0004798	AAAAGUAAUUGUGGUUUUGGCC	45.67	53.01	115.05	102.82
hsa-miR-548c-3p	MIMAT0003285	CAAAAAUCUCAAUUACUUUUGC	97.03	74.89	99.24	83.90
hsa-miR-548c-5p	MIMAT0004806	AAAAGUAAUUGCGGUUUUUGCC	68.64	69.55	113.10	123.73
hsa-miR-548d-3p	MIMAT0003323	CAAAAACCACAGUUUCUUUUGC	116.12	111.10	96.20	115.00
hsa-miR-548d-5p	MIMAT0004812	AAAAGUAAUUGUGGUUUUUGCC	113.16	113.11	77.21	86.91
hsa-miR-548e	MIMAT0005874	AAAAACUGAGACUACUUUUGCA	145.78	153.49	110.18	119.82
hsa-miR-548f	MIMAT0005895	AAAAACUGUAAUUACUUUU	124.25	108.41	106.76	94.04
hsa-miR-548g-3p	MIMAT0005912	AAAACUGUAAUUACUUUUGUAC	105.04	105.26	74.24	91.18
hsa-miR-548h-5p	MIMAT0005928	AAAAGUAAUCGCGGUUUUUGUC	94.87	111.85	97.00	96.21
hsa-miR-548i	MIMAT0005935	AAAAGUAAUUGCGGAUUUUGCC	124.92	105.03	110.61	107.81
hsa-miR-548j	MIMAT0005875	AAAAGUAAUUGCGGUCUUUGGU	86.87	84.89	83.43	76.72
hsa-miR-548k	MIMAT0005882	AAAAGUACUUGCGGAUUUUGCU	87.44	83.31	121.86	127.96
hsa-miR-548l	MIMAT0005889	AAAAGUAUUUGCGGGUUUUGUC	89.33	97.14	100.34	116.48
hsa-miR-548m	MIMAT0005917	CAAAGGUAUUUGUGGUUUUUG	138.79	137.26	117.45	105.50
hsa-miR-548n	MIMAT0005916	CAAAAGUAAUUGUGGAUUUUGU	69.21	64.79	114.78	124.00
hsa-miR-548o-3p	MIMAT0005919	CCAAAACUGCAGUUACUUUUGC	115.90	131.30	108.92	119.18
hsa-miR-548p	MIMAT0005934	UAGCAAAAACUGCAGUUACUUU	53.83	46.66	111.03	92.61
hsa-miR-548q	MIMAT0011163	GCUGGUGCAAAAGUAAUGGCGG	107.40	119.13	116.49	121.05
hsa-miR-548s	MIMAT0014987	AUGGCCAAAACUGCAGUUAUUUU	88.97	92.06	92.33	103.68
hsa-miR-548t-5p	MIMAT0015009	CAAAAGUGAUCGUGGUUUUUG	129.26	134.97	74.13	100.27
hsa-miR-548u	MIMAT0015013	CAAAGACUGCAAUUACUUUUGCG	37.60	43.57	92.51	111.74
hsa-miR-548v	MIMAT0015020	AGCUACAGUUACUUUUGCACCA	114.94	119.66	105.87	112.02
hsa-miR-548w	MIMAT0015060	AAAAGUAACUGCGGUUUUUGCCU	126.02	110.08	104.02	116.45
hsa-miR-548x	MIMAT0015081	UAAAAACUGCAAUUACUUUCA	91.77	90.27	85.77	104.51
hsa-miR-548y	MIMAT0018354	AAAAGUAAUCACUGUUUUUGCC	126.17	116.38	136.58	121.78
hsa-miR-548z	MIMAT0018446	CAAAAACCGCAAUUACUUUUGCA	88.46	93.25	109.52	99.01
hsa-miR-549a	MIMAT0003333	UGACAACUAUGGAUGAGCUCU	93.20	119.07	75.75	82.51
hsa-miR-550a-3p	MIMAT0003257	UGUCUUACUCCCUCAGGCACAU	85.49	106.31	115.03	104.09
hsa-miR-550a-5p	MIMAT0004800	AGUGCCUGAGGGAGUAAGAGCCC	93.78	106.37	81.72	85.83
hsa-miR-550b-3p	MIMAT0018445	UCUUACUCCCUCAGGCACUG	116.06	77.66	98.69	71.76
hsa-miR-551a	MIMAT0003214	GCGACCCACUCUUGGUUUCCA	134.06	119.13	126.79	127.27
hsa-miR-551b-3p	MIMAT0003233	GCGACCCAUACUUGGUUUCAG	120.80	124.02	125.47	120.36
hsa-miR-551b-5p	MIMAT0004794	GAAAUCAAGCGUGGGUGAGACC	110.44	119.87	89.52	80.18
hsa-miR-552	MIMAT0003215	AACAGGUGACUGGUUAGACAA	54.23	63.07	68.29	80.92
hsa-miR-553	MIMAT0003216	AAAACGGUGAGAUUUUGUUUU	145.63	121.75	117.24	96.63
hsa-miR-554	MIMAT0003217	GCUAGUCCUGACUCAGCCAGU	71.42	99.95	88.43	97.82

hsa-miR-555	MIMAT0003219	AGGGUAAGCUGAACCUCUGAU	98.26	117.65	114.53	119.88
hsa-miR-556-3p	MIMAT0004793	AUAUUACCAUUAGCUCAUCUUU	166.97	214.59	139.64	151.56
hsa-miR-556-5p	MIMAT0003220	GAUGAGCUCAUUGUAAUAUGAG	71.76	83.73	105.94	112.97
hsa-miR-557	MIMAT0003221	GUUUGCACGGGUGGGCCUUGUCU	81.77	80.92	107.82	92.22
hsa-miR-558	MIMAT0003222	UGAGCUGCUGUACCAAAAU	94.20	83.14	94.27	91.42
hsa-miR-559	MIMAT0003223	UAAAGUAAAUAUGCACCAAAA	105.62	90.38	132.43	114.57
hsa-miR-561-3p	MIMAT0003225	CAAAGUUUAAGAUCCUUGAAGU	122.98	103.15	117.76	116.60
hsa-miR-562	MIMAT0003226	AAAGUAGCUGUACCAUUUGC	102.21	78.40	115.49	129.94
hsa-miR-563	MIMAT0003227	AGGUUGACAUACGUUUCCC	106.18	95.35	143.68	126.54
hsa-miR-564	MIMAT0003228	AGGCACGGUGUCAGCAGGC	100.47	118.21	103.73	101.09
hsa-miR-566	MIMAT0003230	GGGCGCCUGUGAUCCCAAC	113.13	108.91	104.51	92.30
hsa-miR-567	MIMAT0003231	AGUAUGUUCUUCCAGGACAGAAC	117.48	89.11	121.89	106.66
hsa-miR-568	MIMAT0003232	AUGUAUAAAUGUAUACACAC	94.24	94.35	99.47	114.49
hsa-miR-569	MIMAT0003234	AGUUAAUGAAUCCUGGAAAGU	81.25	81.01	67.65	99.76
hsa-miR-570-3p	MIMAT0003235	CGAAAACAGCAAUUACCUUUGC	49.76	66.94	71.24	77.34
hsa-miR-571	MIMAT0003236	UGAGUUGGCCAUCUGAGUGAG	107.67	78.40	118.41	120.47
hsa-miR-572	MIMAT0003237	GUCCGCUCGGCGGUGGCCCA	88.36	77.57	103.25	103.53
hsa-miR-573	MIMAT0003238	CUGAAGUGAUGUGUAACUGAUCAG	136.29	125.80	125.60	133.26
hsa-miR-574-3p	MIMAT0003239	CACGCUCAUGCACACACCCACA	113.10	125.65	94.22	100.65
hsa-miR-574-5p	MIMAT0004795	UGAGUGUGUGUGUGUGAGUGUGU	103.66	110.34	119.31	99.30
hsa-miR-575	MIMAT0003240	GAGCCAGUUGGACAGGAGC	101.05	119.30	104.48	125.53
hsa-miR-576-3p	MIMAT0004796	AAGAUGUGGAAAAAUUGGAAUC	99.52	77.80	108.49	113.36
hsa-miR-576-5p	MIMAT0003241	AUUCUAAUUUCUCCACGUCUUU	121.62	139.37	98.94	119.51
hsa-miR-577	MIMAT0003242	UAGAUAAAAUAUUGGUACCUG	129.01	118.23	118.17	106.30
hsa-miR-578	MIMAT0003243	CUUCUUGUGCUCUAGGAUUGU	36.24	25.42	92.55	96.92
hsa-miR-579	MIMAT0003244	UUCAUUUGGUAUAAACCGCGAUU	119.91	101.82	88.83	78.31
hsa-miR-580	MIMAT0003245	UUGAGAAUGAUGAAUCAUUAGG	84.13	110.29	83.34	77.92
hsa-miR-581	MIMAT0003246	UCUUGUGUUCUCUAGAUCAGU	119.23	95.48	112.77	117.65
hsa-miR-582-3p	MIMAT0004797	UAACUGGUUGAACAACUGAACC	99.16	114.48	91.38	110.64
hsa-miR-582-5p	MIMAT0003247	UUACAGUUGUUCAACCAGUUACU	189.17	216.00	73.30	89.05
hsa-miR-583	MIMAT0003248	CAAAGAGGAAGGUCCCAUUAC	114.94	109.23	91.18	92.58
hsa-miR-584-5p	MIMAT0003249	UUAUGGUUUGCCUGGGACUGAG	69.99	68.86	79.47	76.33
hsa-miR-585	MIMAT0003250	UGGGCGUAUCUGUAUGCUA	98.12	86.08	97.84	127.84
hsa-miR-586	MIMAT0003252	UAUGCAUUGUAUUUUUAGGUCC	134.76	154.64	101.47	113.44
hsa-miR-587	MIMAT0003253	UUUCCAUAGGUGAUGAGUCAC	106.78	108.51	89.13	67.39
hsa-miR-588	MIMAT0003255	UUGGCCACAAUGGGUUAGAAC	110.85	123.14	93.08	84.88
hsa-miR-589-3p	MIMAT0003256	UCAGAACAAAUGCCGGUUCCCAGA	112.30	105.47	103.22	109.01
hsa-miR-589-5p	MIMAT0004799	UGAGAACCACGUCUGCUCUGAG	96.49	83.97	91.42	97.85
hsa-miR-590-3p	MIMAT0004801	UAAUUUUAUGUAUAAGCUAGU	98.12	98.37	103.94	107.26
hsa-miR-590-5p	MIMAT0003258	GAGCUUAUUCAUAAAAGUGCAG	132.32	132.09	110.04	96.57
hsa-miR-591	MIMAT0003259	AGACCAUGGGUUCUCAUUGU	81.30	78.96	86.34	92.05

hsa-miR-592	MIMAT0003260	UUGUGUCAAUAUGCGAUGAUGU	119.44	112.16	111.90	120.11
hsa-miR-593-3p	MIMAT0004802	UGUCUCUGCUGGGGUUUCU	87.86	115.09	109.29	96.21
hsa-miR-593-5p	MIMAT0003261	AGGCACCAGCCAGGCAUUGCUCAGC	49.59	52.20	123.81	118.36
hsa-miR-595	MIMAT0003263	GAAGUGUGCCGUGGUGUGUCU	131.07	136.90	100.66	100.55
hsa-miR-596	MIMAT0003264	AAGCCUGCCCGGCUCCUCGGG	130.00	95.35	58.61	61.93
hsa-miR-597	MIMAT0003265	UGUGUCACUCGAUGACCACUGU	178.63	153.83	66.09	85.66
hsa-miR-598	MIMAT0003266	UACGUCAUCGUUGUCAUCGUCA	117.10	111.45	99.30	80.76
hsa-miR-599	MIMAT0003267	GUUGUGUCAGUUUAUCAAAC	112.18	124.25	89.86	88.60
hsa-miR-600	MIMAT0003268	ACUUACAGACAAGAGCCUUGCUC	134.72	149.58	103.97	120.53
hsa-miR-601	MIMAT0003269	UGGUCUAGGAUUGUUGGAGGAG	92.80	94.52	86.00	106.79
hsa-miR-602	MIMAT0003270	GACACGGGCGACAGCUGCGGCCC	120.85	119.45	134.62	145.56
hsa-miR-603	MIMAT0003271	CACACACUGCAAUUACUUUUGC	120.21	87.86	99.24	99.90
hsa-miR-604	MIMAT0003272	AGGCUGCGGAAUUCAGGAC	133.97	119.08	137.67	124.68
hsa-miR-605	MIMAT0003273	UAAAUCCCAUGGUGCCUUCUCCU	137.68	135.98	80.57	86.13
hsa-miR-606	MIMAT0003274	AAACUACUGAAAAUCAAAGAU	111.50	105.12	91.75	95.78
hsa-miR-607	MIMAT0003275	GUUCAAAUCCAGAUCUAUAAC	90.25	97.94	94.76	111.40
hsa-miR-608	MIMAT0003276	AGGGGUGGUGUUGGGACAGCUCCGU	41.01	42.25	125.30	122.49
hsa-miR-609	MIMAT0003277	AGGGUGUUUCUCUCAUCUCU	81.87	88.57	100.80	124.03
hsa-miR-610	MIMAT0003278	UGAGCUAAAUGUGUGCUGGGA	167.45	190.86	124.00	131.65
hsa-miR-611	MIMAT0003279	GCGAGGACCCCUCGGGGUCUGAC	116.54	120.62	115.02	95.75
hsa-miR-612	MIMAT0003280	GCUGGGCAGGGCUUCUGAGCUCCUU	70.35	70.08	88.83	94.42
hsa-miR-613	MIMAT0003281	AGGAAUGUUCCUUCUUUGCC	134.37	109.55	91.69	78.40
hsa-miR-614	MIMAT0003282	GAACGCCUGUUCUUGCCAGGUGG	89.95	112.65	119.47	113.59
hsa-miR-615-3p	MIMAT0003283	UCCGAGCCUGGGUCUCCCUCUU	54.98	47.66	115.99	129.44
hsa-miR-615-5p	MIMAT0004804	GGGGGUCCCCGGUGCUCGGAUC	120.93	121.01	99.79	99.48
hsa-miR-616-3p	MIMAT0004805	AGUCAUUGGAGGGUUUGAGCAG	132.32	115.43	123.90	115.96
hsa-miR-616-5p	MIMAT0003284	ACUCAAAACCCUUCAGUGACUU	118.77	112.40	117.82	94.15
hsa-miR-617	MIMAT0003286	AGACUUCCCAUUUGAAGGUGGC	45.95	78.08	83.47	80.71
hsa-miR-618	MIMAT0003287	AAACUCUACUUGUCCUUCUGAGU	97.03	108.61	85.83	105.64
hsa-miR-619	MIMAT0003288	GACCUGGACAUGUUUGUGCCCAGU	118.19	125.35	83.43	123.09
hsa-miR-620	MIMAT0003289	AUGGAGAUAGAUAUAGAAAU	121.70	89.85	96.16	92.90
hsa-miR-621	MIMAT0003290	GGCUAGCAACAGCGCUUACCU	79.47	112.01	113.45	127.01
hsa-miR-622	MIMAT0003291	ACAGUCUGCUGAGGUUGGAGC	94.45	90.18	90.50	101.59
hsa-miR-623	MIMAT0003292	AUCCCUUGCAGGGGCUGUUGGGU	73.99	108.45	101.60	81.24
hsa-miR-624-3p	MIMAT0004807	CACAAGGUAUUGGUAUUACCU	84.73	97.65	104.54	118.42
hsa-miR-624-5p	MIMAT0003293	UAGUACCAGUACCUUGUGUUCA	92.82	83.62	134.99	126.51
hsa-miR-625-3p	MIMAT0004808	GACUAUAGAACUUUCCCCCUCA	110.23	109.75	102.98	112.64
hsa-miR-625-5p	MIMAT0003294	AGGGGGAAAGUUCUAUAGUCC	113.73	87.27	90.08	77.57
hsa-miR-626	MIMAT0003295	AGCUGUCUGAAAAUGUCUU	87.14	87.23	95.28	90.54
hsa-miR-627	MIMAT0003296	GUGAGUCUCUAAGAAAAGAGGA	117.60	124.17	99.75	101.53
hsa-miR-628-3p	MIMAT0003297	UCUAGUAAGAGUGGCAGUCGA	94.97	91.77	100.55	96.31

hsa-miR-628-5p	MIMAT0004809	AUGCUGACAUAUUUACUAGAGG	36.17	37.29	87.81	76.46
hsa-miR-629-3p	MIMAT0003298	GUUCUCCCAACGUAAGCCCAGC	85.17	98.23	75.40	72.00
hsa-miR-629-5p	MIMAT0004810	UGGGUUUACGUUGGGAGAACU	69.99	70.60	87.15	89.40
hsa-miR-630	MIMAT0003299	AGUAUUCUGUACCAGGGAAGGU	168.45	149.05	107.61	75.35
hsa-miR-631	MIMAT0003300	AGACCUGGCCCAGACCUCAGC	113.81	123.69	87.93	94.95
hsa-miR-632	MIMAT0003302	GUGUCUGCUUCCUGUGGGA	107.81	106.51	76.14	107.27
hsa-miR-633	MIMAT0003303	CUAAUAGUAUCUACCACAAUAAA	86.25	85.91	92.53	82.26
hsa-miR-634	MIMAT0003304	AACCAGCACCCCAACUUUGGAC	40.19	67.38	149.97	150.99
hsa-miR-635	MIMAT0003305	ACUUGGGCACUGAAACAAUGUCC	77.40	91.11	83.02	94.48
hsa-miR-636	MIMAT0003306	UGUGCUUGCUCGUCCCGCCCGCA	123.78	106.25	103.68	92.53
hsa-miR-637	MIMAT0003307	ACUGGGGGCUUUCGGGCUCUGCGU	85.38	84.55	95.55	114.24
hsa-miR-638	MIMAT0003308	AGGGAUCGCGGGCGGGUGGCGGCCU	81.94	90.45	108.17	99.90
hsa-miR-639	MIMAT0003309	AUCGCUGCGGUUGCGAGCGCUGU	83.07	69.73	100.30	85.15
hsa-miR-640	MIMAT0003310	AUGAUCCAGGAACCUGCCUCU	91.75	77.92	98.29	80.02
hsa-miR-641	MIMAT0003311	AAAGACAUAGGAUAGAGUCACCUC	87.68	114.19	87.51	99.53
hsa-miR-642a-5p	MIMAT0003312	GUCCCUCUCCAAAUGUGUCUUG	101.22	92.81	121.46	126.07
hsa-miR-642b-3p	MIMAT0018444	AGACACAUUUGGAGAGGGACCC	69.80	85.48	93.46	86.51
hsa-miR-643	MIMAT0003313	ACUUGUAUGCUAGCUCAGGUAG	137.30	103.77	89.86	103.74
hsa-miR-644a	MIMAT0003314	AGUGUGGCUUUCUUAGAGC	63.07	61.89	79.84	77.96
hsa-miR-645	MIMAT0003315	UCUAGGCUGGUACUGCUGA	121.16	103.71	113.91	110.43
hsa-miR-646	MIMAT0003316	AAGCAGCUGCCUCUGAGGC	65.35	84.36	69.58	90.23
hsa-miR-647	MIMAT0003317	GUGGCUGCACUCACUUCCUUC	97.61	97.30	82.19	98.82
hsa-miR-648	MIMAT0003318	AAGUGUGCAGGGCACUGGU	94.90	112.36	105.58	116.13
hsa-miR-649	MIMAT0003319	AAACCUGUGUUGUUCAAGAGUC	71.95	116.54	85.70	107.17
hsa-miR-650	MIMAT0003320	AGGAGGCAGCGCUCUCAGGAC	94.28	81.19	119.84	123.73
hsa-miR-651	MIMAT0003321	UUUAGGAUAAGCUUGACUUUUG	72.23	77.48	85.09	81.03
hsa-miR-652-3p	MIMAT0003322	AAUGGCGCCACUAGGGUUGUG	73.10	105.15	117.80	112.21
hsa-miR-653	MIMAT0003328	GUGUUGAAACAAUCUCUACUG	35.41	45.32	84.29	95.20
hsa-miR-654-3p	MIMAT0004814	UAUGUCUGCUGACCAUCACCUU	165.29	171.83	87.06	82.51
hsa-miR-654-5p	MIMAT0003330	UGGUGGGCCGCAGAACAUGUGC	96.31	70.55	102.90	116.48
hsa-miR-655	MIMAT0003331	AUAAUACAUGGUUAACCUCUUU	37.88	35.70	76.69	62.99
hsa-miR-656	MIMAT0003332	AAUAUUAUACAGUCAACCUCU	109.51	101.46	88.58	81.86
hsa-miR-657	MIMAT0003335	GGCAGGUUCUCACCCUCUCUAGG	103.21	113.15	103.41	131.52
hsa-miR-658	MIMAT0003336	GGCGGAGGGAAGUAGGUCCGUUGGU	98.26	102.80	115.02	104.70
hsa-miR-659-3p	MIMAT0003337	CUUGGUUCAGGGAGGGUCCCCA	91.72	89.94	93.27	91.50
hsa-miR-660-5p	MIMAT0003338	UACCCAUUGCAUAUCGGAGUUG	99.82	110.59	88.72	109.69
hsa-miR-661	MIMAT0003324	UGCCUGGGUCUCUGGCCUGCGCGU	85.17	81.37	77.64	77.33
hsa-miR-662	MIMAT0003325	UCCCACGUUGUGGCCCAGCAG	142.02	148.84	104.51	120.61
hsa-miR-663a	MIMAT0003326	AGGCGGGGCGCCGCGGGACCGC	94.28	108.69	93.54	115.28
hsa-miR-663b	MIMAT0005867	GGUGGCCCGGCCGUGCCUGAGG	109.04	117.53	120.75	105.99
hsa-miR-664a-3p	MIMAT0005949	UAUUCAUUUAUCCCCAGCCUACA	126.57	128.25	137.13	116.48

hsa-miR-664a-5p	MIMAT0005948	ACUGGCUAGGGAAAAUGAUUGGAU	94.37	92.67	90.59	92.90
hsa-miR-665	MIMAT0004952	ACCAGGAGGCUGAGGCCCCU	78.74	78.19	97.22	108.24
hsa-miR-668	MIMAT0003881	UGUCACUCGGCUCGGCCCACUAC	165.38	150.52	102.71	103.56
hsa-miR-670	MIMAT0010357	GUCCCUGAGUGUAUGUGGUG	68.55	62.72	76.27	93.80
hsa-miR-671-3p	MIMAT0004819	UCCGGUUCUCAGGGCUCCACC	105.48	90.56	93.37	70.21
hsa-miR-671-5p	MIMAT0003880	AGGAAGCCCUGGAGGGGCUGGAG	109.95	112.56	103.46	84.10
hsa-miR-675-3p	MIMAT0006790	CUGUAUGCCCUCACCGCUCA	88.75	121.94	77.66	69.22
hsa-miR-675-5p	MIMAT0004284	UGGUGCGGAGAGGGCCCACAGUG	90.28	89.87	94.29	98.64
hsa-miR-676-3p	MIMAT0018204	CUGUCCUAAGGUUGUUGAGUU	101.25	92.18	118.13	121.46
hsa-miR-676-5p	MIMAT0018203	UCUUCAACCUCAGGACUUGCA	112.15	134.47	74.40	74.27
hsa-miR-708-3p	MIMAT0004927	CAACUAGACUGUGAGCUUCUAG	93.41	103.93	114.80	98.90
hsa-miR-708-5p	MIMAT0004926	AAGGAGCUUACAAUCUAGCUGGG	61.24	74.31	49.38	50.05
hsa-miR-711	MIMAT0012734	GGGACCCAGGGAGAGACGUAAG	83.84	97.62	122.59	105.74
hsa-miR-7-1-3p	MIMAT0004553	CAACAAAUCACAGUCUGCCAUA	94.57	117.88	99.91	111.71
hsa-miR-718	MIMAT0012735	CUUCCGCCCCGCCGGGCGUCG	63.19	74.97	86.20	102.11
hsa-miR-720	MIMAT0005954	UCUCGCUGGGGCCUCCA	141.27	151.30	92.18	113.06
hsa-miR-7-2-3p	MIMAT0004554	CAACAAAUCCCAGUCUACCUAA	54.23	63.78	86.13	58.65
hsa-miR-744-3p	MIMAT0004946	CUGUUGCCACUAACCUCAACCU	57.82	61.09	84.11	75.93
hsa-miR-744-5p	MIMAT0004945	UGCGGGGCUAGGGCUAACAGCA	107.81	122.87	105.94	90.05
hsa-miR-758-3p	MIMAT0003879	UUUGUGACCUGGUCCACUAACC	105.83	99.86	80.04	80.50
hsa-miR-759	MIMAT0010497	GCAGAGUGCAAACAAUUUUGAC	82.42	86.01	81.71	81.63
hsa-miR-7-5p	MIMAT0000252	UGGAAGACUAGUGAUUUUGUUGU	110.83	135.51	134.86	155.41
hsa-miR-760	MIMAT0004957	CGGCUCUGGGUCUGUGGGGA	93.26	88.51	94.77	90.46
hsa-miR-761	MIMAT0010364	GCAGCAGGGUGAAACUGACACA	94.41	114.64	106.16	95.78
hsa-miR-762	MIMAT0010313	GGGGCUGGGGCCGGGGCCGAGC	83.84	88.91	109.07	101.17
hsa-miR-764	MIMAT0010367	GCAGGUGCUCACUUGUCCUCCU	39.51	32.29	89.52	98.47
hsa-miR-765	MIMAT0003945	UGGAGGAGAAGGAAGGUGAUG	96.26	110.60	70.10	86.99
hsa-miR-766-3p	MIMAT0003888	ACUCCAGCCCCACAGCCUCAGC	80.97	81.03	57.97	61.65
hsa-miR-767-3p	MIMAT0003883	UCUGCUCAUACCCCAUGGUUUCU	101.25	103.97	97.73	107.04
hsa-miR-767-5p	MIMAT0003882	UGCACCAUGGUUGUCUGAGCAUG	68.52	44.52	101.30	94.95
hsa-miR-768-3p	MIMAT0003947	UCACAAUGCUGACACUCAAACUGCUGAC	71.40	69.62	106.92	122.69
hsa-miR-768-5p	MIMAT0003946	GUUGGAGGAUGAAAGUACGGAGUGAU	98.34	87.37	102.16	91.09
hsa-miR-769-3p	MIMAT0003887	CUGGGAUCUCCGGGGUCUUGGUU	105.62	91.73	97.34	95.00
hsa-miR-769-5p	MIMAT0003886	UGAGACCUCUGGGUUCUGAGCU	75.82	55.14	106.71	96.57
hsa-miR-770-5p	MIMAT0003948	UCCAGUACCACGUGUCAGGGCCA	87.59	97.61	99.33	100.81
hsa-miR-801	MIMAT0004209	GAUUGCUCUGCGUGCGGAAUCGAC	88.20	95.78	94.28	81.16
hsa-miR-802	MIMAT0004185	CAGUAACAAAGAUUCAUCCUUGU	94.61	76.71	89.70	90.71
hsa-miR-873-5p	MIMAT0004953	GCAGGAACUUGUGAGUCUCCU	80.07	95.50	70.08	81.67
hsa-miR-874	MIMAT0004911	CUGCCCUGGCCCGAGGGACCGA	137.68	91.52	79.84	102.48
hsa-miR-875-3p	MIMAT0004923	CCUGGAAACACUGAGGUUGUG	122.38	125.22	115.55	95.51
hsa-miR-875-5p	MIMAT0004922	UAUACCUCAGUUUUAUCAGGUG	37.73	43.79	101.95	83.14

hsa-miR-876-3p	MIMAT0004925	UGGUGGUUUACAAAGUAAUUCA	73.85	78.13	87.32	116.72
hsa-miR-876-5p	MIMAT0004924	UGGAUUUCUUUGUGAAUCACCA	97.12	113.45	60.51	65.37
hsa-miR-877-3p	MIMAT0004950	UCCUCUUCUCCCUCCUCCCAG	117.51	100.18	102.96	99.90
hsa-miR-877-5p	MIMAT0004949	GUAGAGGAGAUGGCGCAGGG	73.02	73.64	108.42	67.22
hsa-miR-885-3p	MIMAT0004948	AGGCAGCGGGGUGUAGUGGAUA	57.89	61.98	86.57	84.46
hsa-miR-885-5p	MIMAT0004947	UCCAUUACACUACCCUGCCUCU	106.79	86.32	89.21	109.80
hsa-miR-886-3p	MIMAT0004906	CGCGGGUGCUUACUGACCCUU	100.35	81.40	109.54	122.87
hsa-miR-887	MIMAT0004951	GUGAACGGGCGCCAUCCCGAGG	62.30	76.71	106.15	98.88
hsa-miR-888-3p	MIMAT0004917	GACUGACACCUCUUUGGGUGAA	108.17	115.69	92.25	78.37
hsa-miR-888-5p	MIMAT0004916	UACUCAAAAAGCUGUCAGUCA	102.82	112.93	92.87	101.02
hsa-miR-889	MIMAT0004921	UUAAUAUCGGACAACCAUUGU	72.63	67.97	93.21	87.02
hsa-miR-890	MIMAT0004912	UACUUGGAAAGGCAUCAGUUG	65.22	88.07	81.41	98.38
hsa-miR-891a	MIMAT0004902	UGCAACGAACCUGAGCCACUGA	92.67	101.13	89.47	94.62
hsa-miR-891b	MIMAT0004913	UGCAACUUACCUGAGUCAUUGA	50.33	53.01	62.65	55.22
hsa-miR-892a	MIMAT0004907	CACUGUGUCCUUUCUGCGUAG	104.75	95.86	82.90	82.43
hsa-miR-892b	MIMAT0004918	CACUGGCUCCUUUCUGGGUAGA	83.79	71.63	110.61	114.82
hsa-miR-920	MIMAT0004970	GGGGAGCUGUGGAAGCAGUA	93.07	123.77	88.24	77.31
hsa-miR-921	MIMAT0004971	CUAGUGAGGGACAGAACCAGGAUUC	91.53	101.11	101.03	115.22
hsa-miR-922	MIMAT0004972	GCAGCAGAGAAUAGGACUACGUC	93.52	97.60	129.61	105.75
hsa-miR-923	MIMAT0004973	GUCAGCGGAGGAAAAGAAACU	118.77	89.12	128.60	112.90
hsa-miR-924	MIMAT0004974	AGAGUCUUGUGAUGUCUUGC	93.20	96.02	92.84	106.62
hsa-miR-92a-1-5p	MIMAT0004507	AGGUUGGGAUCGGUUGCAAUGCU	108.45	114.19	97.01	109.34
hsa-miR-92a-2-5p	MIMAT0004508	GGGUGGGGAUUUGUUGCAUUAC	67.38	85.27	66.47	75.69
hsa-miR-92a-3p	MIMAT0000092	UAUUGCACUUGUCCCGGCCUGU	48.52	38.78	93.53	93.68
hsa-miR-92b-3p	MIMAT0003218	UAUUGCACUCGUCCCGGCCUCC	57.25	59.50	103.66	84.91
hsa-miR-92b-5p	MIMAT0004792	AGGGACGGGACGCGGUGCAGUG	111.46	105.36	99.51	116.88
hsa-miR-933	MIMAT0004976	UGUGCGCAGGGAGACCUCUCCC	65.24	77.59	101.95	123.13
hsa-miR-93-3p	MIMAT0004509	ACUGCUGAGCUAGCACUUCCCG	124.65	82.27	142.11	122.47
hsa-miR-934	MIMAT0004977	UGUCUACUACUGGAGACACUGG	132.53	142.14	108.65	109.76
hsa-miR-935	MIMAT0004978	CCAGUUACCGCUUCCGCUACCGC	187.10	169.72	144.41	140.35
hsa-miR-93-5p	MIMAT0000093	CAAAGUGCUGUUCGUGCAGGUAG	86.70	83.41	116.88	130.70
hsa-miR-936	MIMAT0004979	ACAGUAGAGGGAGGAAUCGCAG	116.68	118.78	103.33	111.71
hsa-miR-937-3p	MIMAT0004980	AUCCGCGCUCUGACUCUCUGCC	119.13	105.37	112.64	111.38
hsa-miR-938	MIMAT0004981	UGCCCUUAAAGGUGAACCCAGU	123.02	111.39	121.89	126.93
hsa-miR-939-5p	MIMAT0004982	UGGGGAGCUGAGGCUCUGGGGGUG	110.59	108.45	112.27	130.58
hsa-miR-9-3p	MIMAT0000442	AUAAAGCUAGAUAACCGAAAGU	107.51	105.23	100.13	105.42
hsa-miR-940	MIMAT0004983	AAGGCAGGGCCCCCGCUCCCC	77.40	57.21	96.75	119.53
hsa-miR-941	MIMAT0004984	CACCCGGCUGUGUGCACAUGUGC	136.56	146.05	119.31	108.48
hsa-miR-942	MIMAT0004985	UCUUCUCUGUUUUGGCCAUGUG	111.97	110.97	90.50	78.62
hsa-miR-943	MIMAT0004986	CUGACUGUUGCCGUCCUCCAG	78.15	118.04	92.26	112.17
hsa-miR-944	MIMAT0004987	AAAUUAUUGUACAUCGGAUGAG	128.29	122.80	110.05	94.11

hsa-miR-95	MIMAT0000094	UUCAACGGGUAUUUAUUGAGCA	136.14	127.26	109.80	86.46
hsa-miR-9-5p	MIMAT0000441	UCUUUGGUUAUCUAGCUGUAUGA	55.86	71.68	75.75	83.11
hsa-miR-96-3p	MIMAT0004510	AAUCAUGUGCAGUGCCAAUAUG	100.66	77.09	116.55	104.68
hsa-miR-96-5p	MIMAT0000095	UUUGGCACUAGCACAUUUUUGCU	114.35	112.02	120.88	109.32
hsa-miR-98-5p	MIMAT0000096	UGAGGUAGUAAGUUGUAUUGUU	22.99	38.54	68.25	57.76
hsa-miR-99a-3p	MIMAT0004511	CAAGCUCGCUUCUAUGGGUCUG	122.63	101.32	88.53	92.63
hsa-miR-99a-5p	MIMAT0000097	AACCCGUAGAUCCGAUCUUGUG	102.71	82.64	104.46	91.93
hsa-miR-99b-3p	MIMAT0004678	CAAGCUCGUGUCUGUGGGUCCG	140.73	160.55	117.33	98.37
hsa-miR-99b-5p	MIMAT0000689	CACCCGUAGAACCGACCUUGCG	116.91	134.23	93.36	101.82
