# Supplementary material for: Casz1 and Znf101/Zfp961 differentially regulate apolipoproteins A1 and B, alter plasma lipoproteins, and reduce atherosclerosis
Source: JCI Insight. 2025 Jan 9;10(1):e182260. doi: 10.1172/jci.insight.182260 (PMC11721306; doi:10.1172/jci.insight.182260)
Supplement: Unedited blot and gel images [file jciinsight-10-182260-s139.pdf]

Main fig 5C

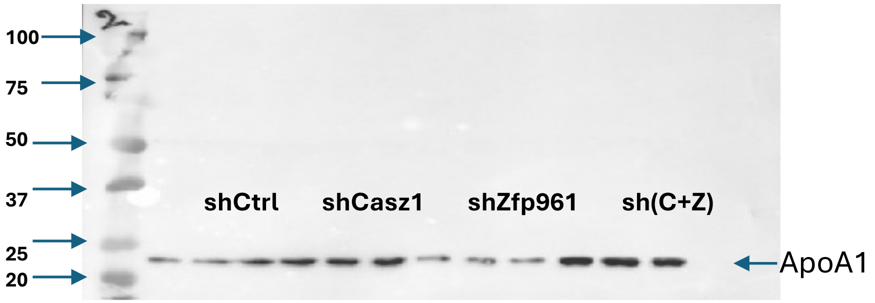

Supplementery fig 8 (B)

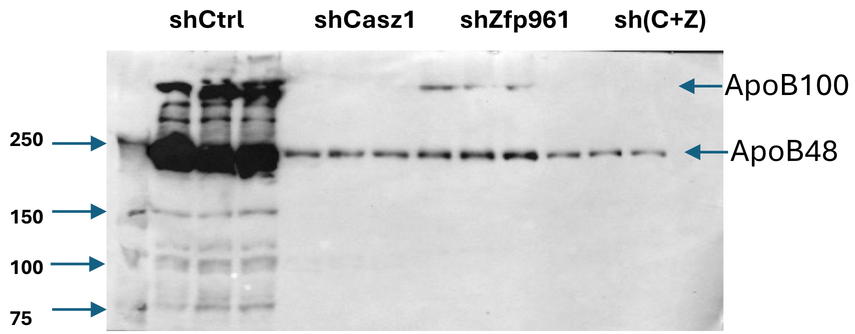

Supplementery fig 8 (C)

**Apolipoprotein A-I/ApoA1 Antibody**	**NOVUS**	**NBP2-15429**	**1:1000 dilution**
**Apolipoprotein B (APOB), Polyclonal Antibody**	MyBiosource	MBS2006107	1:1000 dilution

Supplementary Fig 2

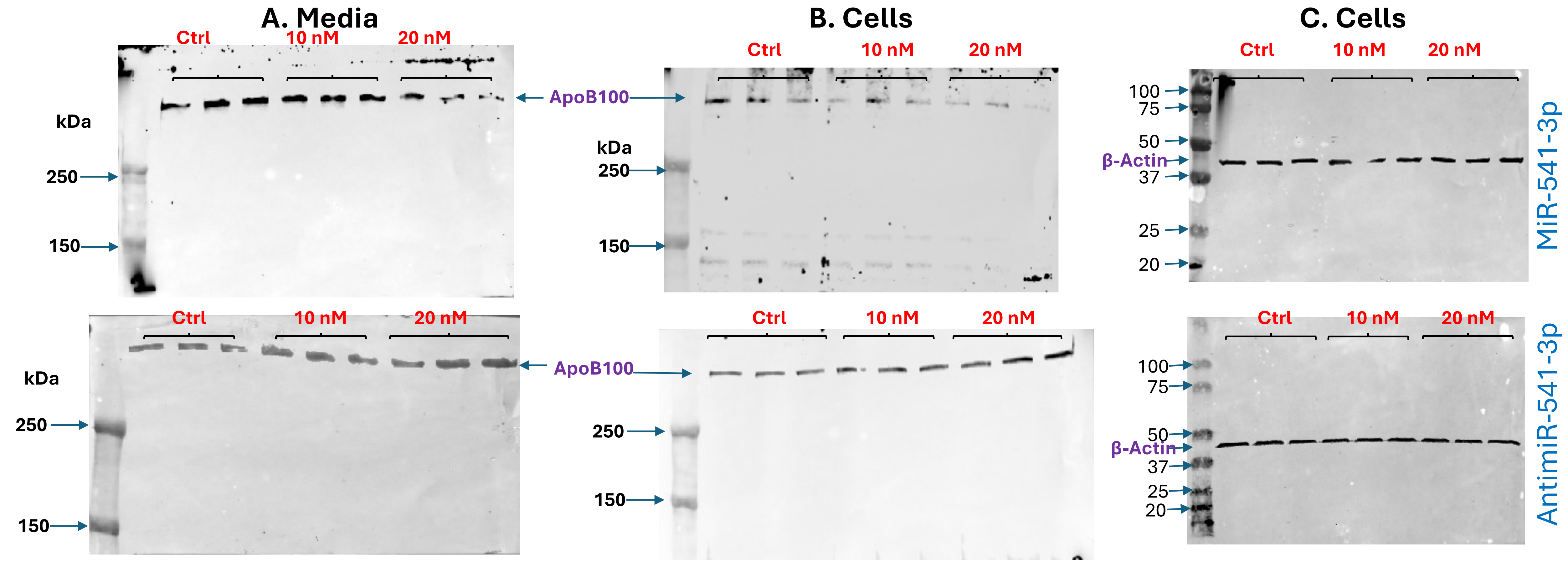

Apolipoprotein B (APOB), monoclonal Antibody 1D1	MBS465020	My BioSource	1:1000 dilution

Supplementary Fig 2

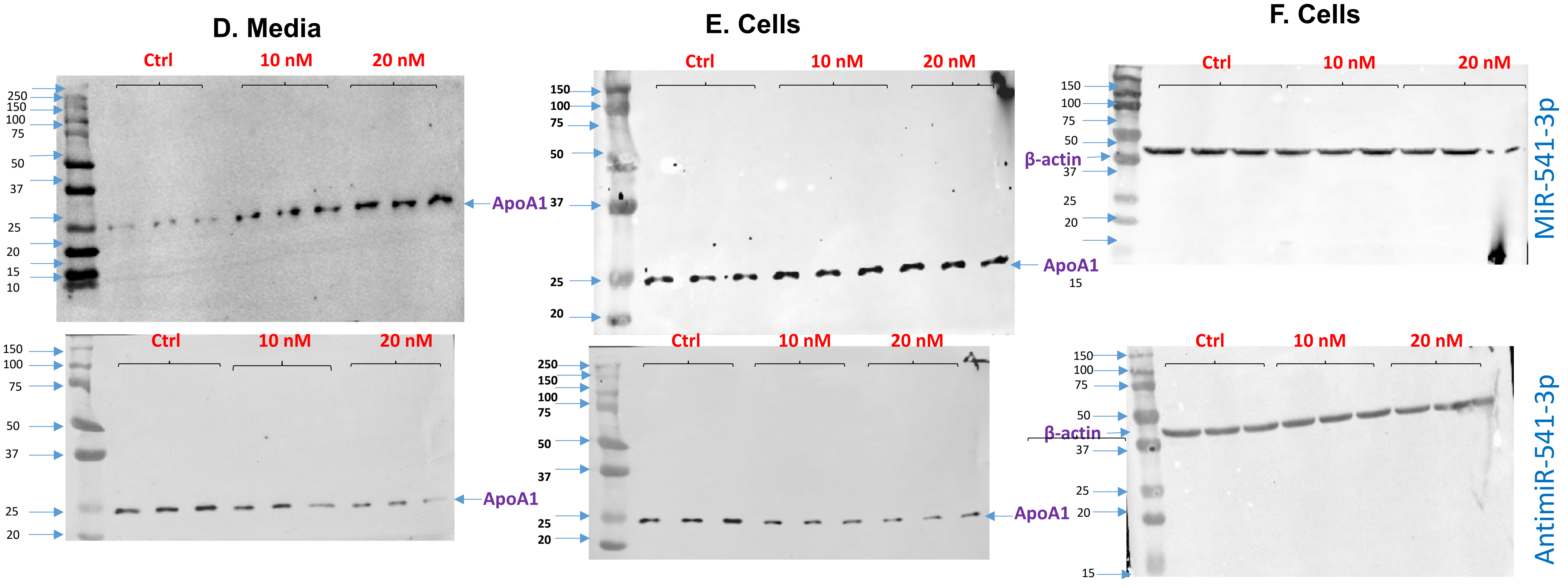

Apolipoprotein A-I/ApoA1 Antibody	NOVUS	NBP2-15429	1:1000 dilution

Supplementary fig 7

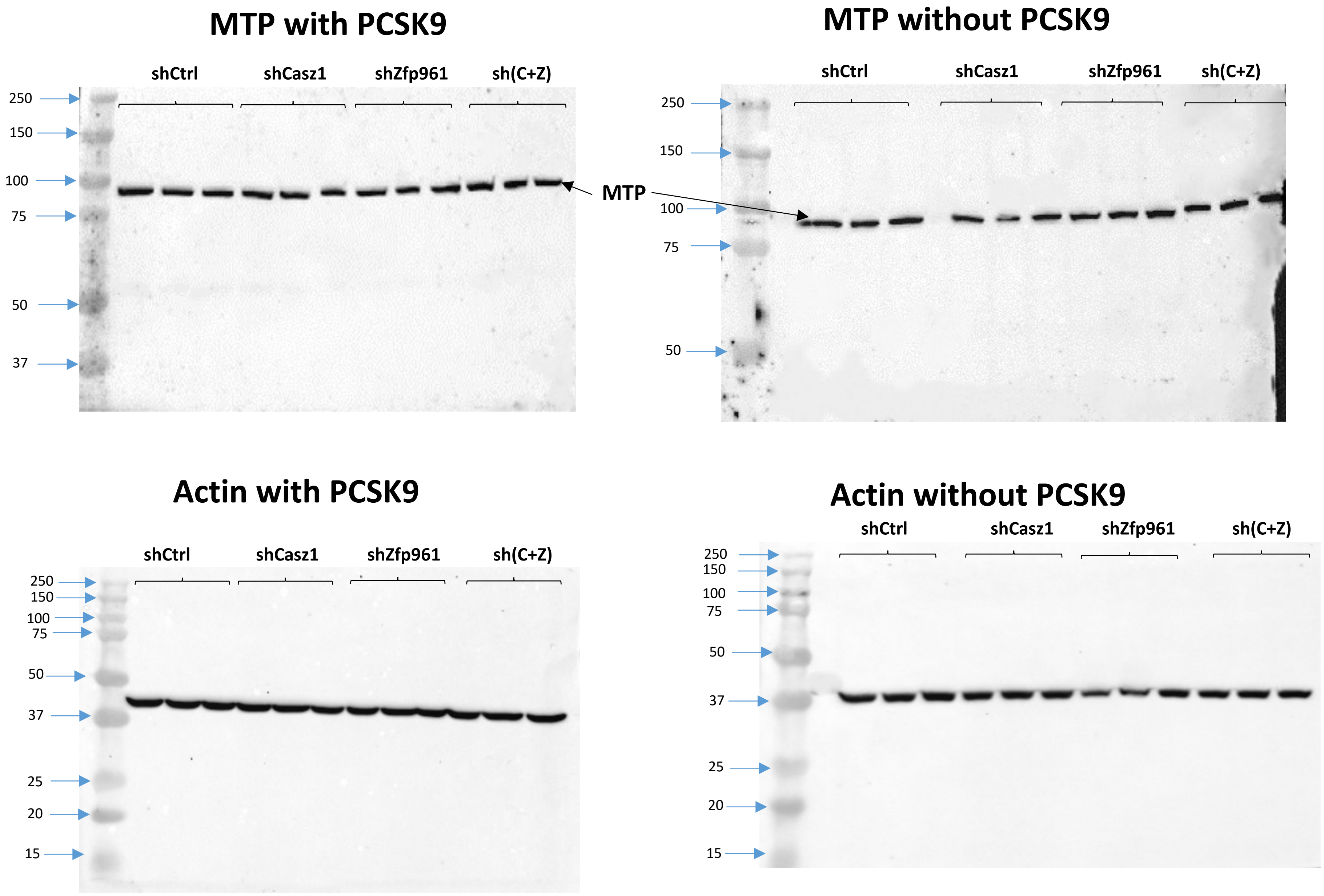

Mouse anti-human MTP	612022	BD Biosciences	1:1000 dilution

Supplementary fig 7

ABCA1 with PCSK9

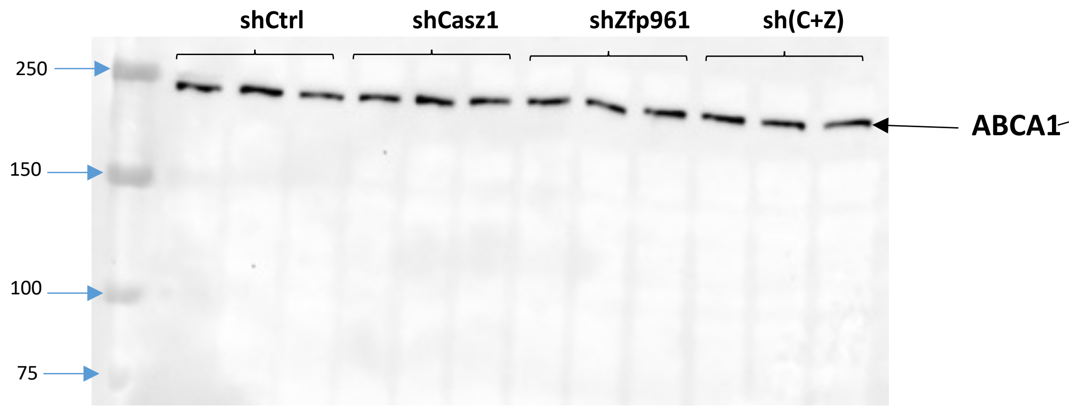

ABCA1 without PCSK9

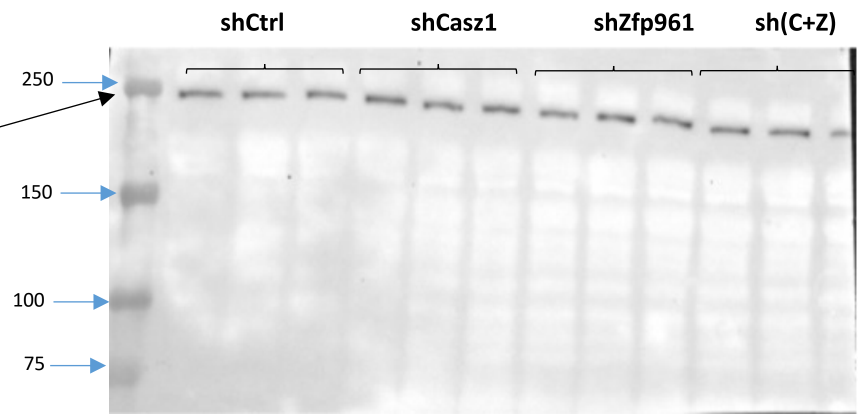

Actin with PCSK9

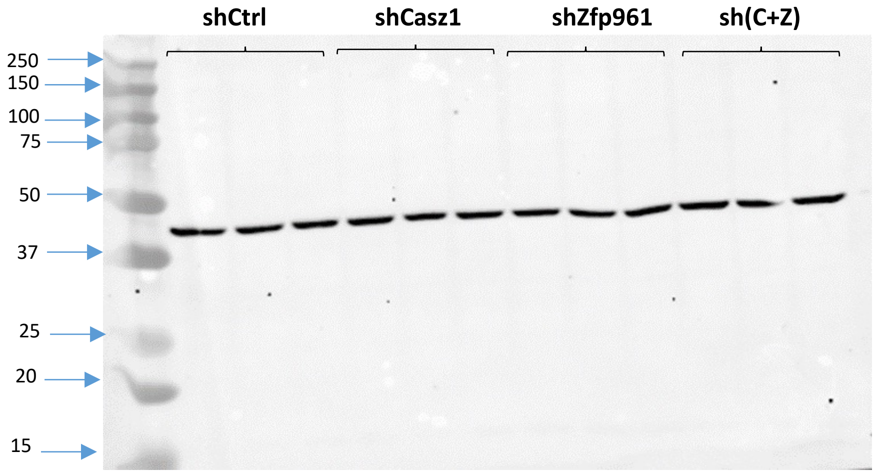

Actin without PCSK9

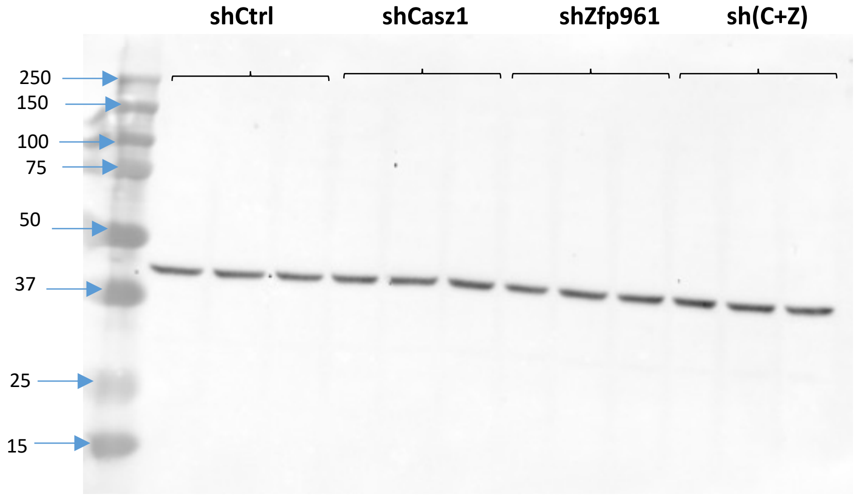

Mouse ABCA1	NB400-105	Novus Biologicals	1:1000 dilution
